# Supplementary material for: Post-transcriptional regulation of several biological processes involved in latex production in Hevea brasiliensis
Source: PeerJ. 2020 Apr 29;8:e8932. doi: 10.7717/peerj.8932 (PMC7195832; doi:10.7717/peerj.8932)
Supplement: Table S1 [file peerj-08-8932-s001.docx]

| **Degradome library** | **Bark** | **Leaf** | **Reproductive tissues** | **Embryo** | **Latex** | **Root** |
| --- | --- | --- | --- | --- | --- | --- |
| **Initial Number of Reads** | 94,348 | 165,538 | 194,205 | 18,160 | 300,090 | 121,924 |
| **After Cutadapt** | 43,534 | 62,242 | 55,561 | 9,283 | 134,611 | 68,571 |
| **%GC** | 43 | 42 | 43 | 44 | 41 | 45 |
