# Supplementary material for: Post-transcriptional regulation of several biological processes involved in latex production in Hevea brasiliensis
Source: PeerJ. 2020 Apr 29;8:e8932. doi: 10.7717/peerj.8932 (PMC7195832; doi:10.7717/peerj.8932)
Supplement: Table S2 — Small RNA from the latex of healthy and TPD-affected trees, from juvenile plants and from mature and young leaves. Filtered_reads: t he reads which were not mapped to mRNA, rRNA, tRNA and known miRNA. Cluster_number: the number of reads cluster which was meant to get candidate regions for miRNA. Cluster_reads: all the reads number which is the sum number of each cluster. Extracted_seq: the number of sequences which was used to create precursor and mature sequence and gff file for candidate clusters. Extracted_seq_after_filter: the number of sequences which was filtered based on candidate miRNA (hairpin) sequence and structure attribute values. Extracted_seq_unique: the number of sequences which was non-redundant hairpin according to the genome position. Candidate_attribute_filter: the number of sequences which was filtered by attribute values such as default MEF (minimal folding free energy) and AMFE (adjusted mfe) with non-redundant sequence. [file peerj-08-8932-s002.docx]

| Sample  Statistics | **Latex**  **(healthy trees)** | **Latex**  **(TPD trees)** | **Young plants (root, stem, bark)** | **Mature leaves** | **Young leaves** |
| --- | --- | --- | --- | --- | --- |
| **Filtered_reads** | 582,097 | 473,723 | 238,692 | 6,579,219 | 6,909,337 |
| **Cluster_number** | 378,894 | 340,712 | 236,364 | 4,165,732 | 4,478,914 |
| **Cluster_reads** | 2,437,187 | 1,932,630 | 977,459 | 38,441,622 | 36,705,385 |
| **Extracted_seq** | 13,414 | 10,777 | 5,417 | 117,092 | 178,082 |
| **Extracted_seq_after_filter** | 507 | 445 | 215 | 5,216 | 8,152 |
| **Extracted_seq_unique** | 485 | 421 | 206 | 5,068 | 7,913 |
| **Candidate_attribute_filter** | 86 | 104 | 61 | 605 | 983 |
| **Total number of non-redundant hairpins** | 1042 | | | | |
