# Supplementary material for: Post-transcriptional regulation of several biological processes involved in latex production in Hevea brasiliensis
Source: PeerJ. 2020 Apr 29;8:e8932. doi: 10.7717/peerj.8932 (PMC7195832; doi:10.7717/peerj.8932)
Supplement: Figure S1 [file peerj-08-8932-s023.pdf]

>Arabidopsis-thaliana_AT5G21030.1_ARATH

-----------------------MDTT---------------------------------

---------LPPPQ--------------------HMER----------------------
-----EPLK------------------------SKSSLLPMTRRGNGSK-GQK-IL-LLT
NHFRVNFRKPNS-HNFFHYSVTI-------------------------------------
----TYE-DGSPLLAKGFGRKILEKVQQTC----QADLGCKHFAYDGDKNLYTVGP-LPR
SSLDFSVVLETAP------------------------SRRNADKRLKLPHQSKKFNVAIL
FAPPEIPMEAIANALQGKKTK---HLLDAIRVMDCILSQNAARQ-GCLLVRQSFFHNDAK
-----YFANIGEGVDCCK--------------------------G-------FHSS-----
-----------FRTTQGGLSLNIDV-STAMIVKPGPVVDFLIA---------NQGVNDPF
SINWKKAKNTLKNLRVKVLPSNQE---YKITGLSGLHCKDQT-----------------F
TWKKRNQNREFEE---VEITVSDYFTRIREIELRYSGGLPC--INVGKP-NRPTYFPIEL
CELVSLQRYTKALTKFQRSNLIKESRQN-PQQRIGVLTR------------------ALK
TSNYNDDPMLQ-ECGVRIGSDFTQVEGRVLPTPKLK----AGKE--QDIYPI---NGSWN
FKNK----PATVTRW--AVVNFSARCD------PQKIIDDLTRCGKMKGINVDS-PYHVV
--------FEENPQFKDATGSVRVDKMFQHLQSILGE-------VPPKFLLCILEKKNSD
V-Y---EKSCSM---WNCECIVPPQNL------N--D----QYLTNLLLKINAK------
----LGGLNSVLDMELSGTMP--LVMR---VPTIIIGMDVSHG----SPGQSDHIPSIAA
VVSSREWPLISKYRACVRTQSPKVEMIDSLFKPVSDKDD----QGIMRELLLDFHSSSGK
K-PNHIIIFR---DGVSESQFNQVLNIELDQM---------------------MQINHHT
KFFQTES----------PNNVLPGTIIDSNICHQHNNDFYLCAHA---------GKIGTT
RPTHYHVLYDEIGFDTDQLQELVHSLSYVYQRSTTAISLVAPICYAHLAAAQMATAMKFE
-------------------D-MSETSSSHGGITTAGAVPVPPMPKLNTNV----------
------------------------AS----------------------------------

-------------SMF-------------------------------FC-----------

>Arabidopsis-thaliana_AT5G21150.1_ARATH

-----------------------MDSDEPNG------------------SG---------

---------LPPPP----------PFVPANLVP---------------------------
-----------------------EV-EPV----KKNILLPMARPRGSGSKGQK-IP-LLT

NHFGVKFNKPS--GYFFHYSVAI-------------------------------------
----NYE-DGRPVEAKGIGRKILDKVQETY----QSDLGAKYFAYDGEKTLFTVGA-LPS
NKLDFSVVLEEIPSSRNHA---------------GNDTNDADRKRSRRPNQTKKFMVEIS
YA-AKIPMQAIASALQGKETE---NLQDALRVLDIILRQSAARQ-GCLLVRQSFFHNDVK
-----NFVPIGGGVSGCR-------------------------G-------FHSS-----
-----------FRTTQGGLSLNIDT-STTMIVQPGPVVDFLLA---------NQNKKDPY
GMDWNKARRVLKNLRVQITLSNRE---YKISGLSEHSCKDQL-----------------F
TWRKPNDKGEFEE---VEITVLNYYKER-NIEVRYSGDFPC--INVGKP-KRPTYFPIEF
CNLVSLQRYTKSLTNFQRAALVEKSRQK-PPERMASLTK------------------GLK
DSNYNADPVLQ-DSGVSIITNFTQVEGRILPTPMLK----VGKG--ENLSPI---KGKWN
FMRKTLAEPTTVTRW--AVVNFSARCD------TNTLIRDLIKCGREKGINVEP-PFKDV
--------INENPQFRNAPATVRVENMFEQIKSKLP--------KPPLFLLCILAERKNS
DVYGPWKKKNLVDLGIVTQCI-APTRL------N--D----QYLTNVLLKINAK------
----LGGLNSLLAMERSPAMP--KVTQ---VPTIIVGMDVSHG----SPGQ-SDIPSIAA
VVSSRQWPLISKYKACVRTQSRKMEMIDNLFKPVNGKD-----EGMFRELLLDFYYSSEN
RKPEHIIIFR---DGVSESQFNQVLNIELDQMMQACKFL--DDTWHPKFTVIVAQKNHHT
KFFQSRG----------PDNVPPGTIIDSQICHPRNFDFYLCAHA---------GMIGTT
RPTHYHVLYDEIGFATDDLQELVHSLSYVYQRSTTAISVVAPVCYAHLAAAQMGTVMKYE
-------------------E-LSETSSSHGGITTPGAVPVPPMPQLHNNV----------
------------------------ST----------------------------------

-------------SMF-------------------------------FC-----------

>Arabidopsis-thaliana_AT2G27040.1_ARATH

-----------------------MDSTNGNGADLESANGANGSGVT---EA---------

---------LPPPP----------PVIPPNVEPVRVKT----------------------
-----ELAEK-----------------------KGPVRVPMARKGFGTR-GQK-IP-LLT
NHFKVDV--ANLQGHFFHYSVAL-------------------------------------
----FYD-DGRPVEQKGVGRKILDKVHQTY----HSDLDGKEFAYDGEKTLFTYGA-LPS
NKMDFSVVLEEVSATRANG-------N--GSPNGNESPSDGDRKRLRRPNRSKNFRVEIS
YA-AKIPLQALANAMRGQESE---NSQEAIRVLDIILRQHAARQ-GCLLVRQSFFHNDPT
-----NCEPVGGNILGCR-------------------------G-------FHSS-----
-----------FRTTQGGMSLNMDV-TTTMIIKPGPVVDFLIA---------NQNARDPY
SIDWSKAKRTLKNLRVKVSPSGQE---FKITGLSDKPCREQT-----------------F
ELKKRNPNENGEFET-TEVTVADYFRDTRHIDLQYSADLPC--INVGKP-KRPTYIPLEL

CALVPLQRYTKALTTFQRSALVEKSRQK-PQERMTVLSK------------------ALK
VSNYDAEPLLR-SCGISISSNFTQVEGRVLPAPKLK----MGCG--SETFPR---NGRWN
FNNKEFVEPTKIQRW--VVVNFSARCN------VRQVVDDLIKIGGSKGIEIAS-PF-QV
--------FEEGNQFRRAPPMIRVENMFKDIQSKLP--------GVPQFILCVLPDKKNS
DLYGPWKKKNLTEFGIVTQCM-APTRQP-----N--D----QYLTNLLLKINAK------
----LGGLNSMLSVERTPAFT--VISK---VPTIILGMDVSHG----SPGQ-SDVPSIAA
VVSSREWPLISKYRASVRTQPSKAEMIESLVK-----KNGTEDDGIIKELLVDFYTSSNK
RKPEHIIIFR---DGVSESQFNQVLNIELDQIIEACKLL--DANWNPKFLLLVAQKNHHT
KFFQPTS----------PENVPPGTIIDNKICHPKNNDFYLCAHA---------GMIGTT
RPTHYHVLYDEIGFSADELQELVHSLSYVYQRSTSAISVVAPICYAHLAAAQLGTFMKFE
-------------------D-QSETSSSHGGITAPGPISVAQLPRLKDNV----------
------------------------AN----------------------------------

-------------SMF-------------------------------FC-----------

>Populus-trichocarpa__POPTR_0008s01100_POPTR

-----------------------MESSDSR-------------------KD---------

---------LPPPP----------AIIPADVVKTELGP----------------------
-----TCE---------------QT-KKA----ATPKRVPMARRGYGAK-GQR-IQ-LLT
NHFKVAV--PKSNDHFYQYSVAL-------------------------------------
----FYE-DGHPTDGKGIGRKVMDKVQETY----DSELEGKQLAYDGEKTLFTTGS-LPH
NKLEFTVVLEDVSLTRGGD-------N--DSSRGNGSPSESDQKRRKRPYHSKTIKVQIS
YA-TKIPVQAIAAVLQGQESE---HFQEAVRVLDIVLRQNAARQ-GCLLVRQSFFHNNPR
-----NFVELGGGVMGCR------------------------G-------FHSS-----
-----------FRAAQDGLSLNIDV-STTMIVKPGPVVDFLIM---------NQNVRDPY
HIDWTKAKRMLKNLRIKTNHSNTE---YKITGLTEKSCREQT-----------------F
SLNQRSGRDGDGEVQTIEVTVYDYFVNHRNMGLQYSADFPC--INVGKP-KRPSYFPLEL
CNLVSLQRYTKALSSLQRASLVEKSRQK-PQERMRSLTD------------------ALR
SSNYDADPMLR-SSGISISAQFTQVEGRVLSAPRLK----VGNG--EDFFPR---NGRWN
FNNKKLVDPVKIEKW--AIVNFSARCD------IRYLCNNLIKCGDMKGISISN-PF-EV
--------FEESPQFRRESAPVRVERMFEAIKSKLP--------GPPQFLLCILPERKNS
DIYGPWKRKNLSDLGIVTQCI-APTKV------N--D----QYLTNVLLKINAKVEFCNV
IPFELGGMNSLLSIEHAPSIP--LVSK---LPTLILGMDVSHG----SPGH-SDVPSIAA
VVSSRHWPLISRYRASVRTQSQKVEMIANLFKPVAGTRE---DQGIIRESLLDFYSSSGK
RKPDQIIIFR---DGVSESQFIQVLNIELEQIIEACKFL--DENWCPKFMVIVAQKNHHT

KFFQSGS----------PDNVPPGTVIDNKVCHPRNNDFYMCAHA---------GMIGTT
RPTHYHVLHDELGFSADDLQELVHSLSYVYQRSTTAISVVAPICYAHLAASQMTQFIKFD
-------------------D-LSDTSSSHGGVTVPGAVPVPELPRLHNNV----------
------------------------SS----------------------------------

-------------SMF-------------------------------FC-----------

>Populus-trichocarpa__POPTR_0001s22710_POPTR

-----------------------MESADEQNGNGSQ-------------EA---------

---------LPPPP----------PDVPPNVVPVKAEP----------------------
-----EPVK------------------------KKPLRVPIARRGLGSK-GQK-MP-LLT
NHFKVNVTNTE--GYFFHYCVSL-------------------------------------
----AYE-DGRPVDGKGVGRKVIDRVHETY----DTEFG-KDFAYDGEKSLFTVGP-LPR
NKLEFTVVLEDVVSNRNNG-------N--ASPDGHGSPNEGDRKRLRRPYHSKTFKVEIS
FA-AKIPMQAIANALRGQESE---NSQEAFRVLDIILRQHAAKQ-GCLLVRQSFFHNDPK
-----NFVDLGGGVLGCR-------------------------G-------FHSS-----
-----------FRTSQGGLSLNIDV-STTMIIQPGPVVDFLIA---------NQNVRDPF
SLDWAKAKRMLKNLRVKASPSNQE---YKITGLSEKTCKEQM-----------------F
QLKQKNGGDGGIEA--VEITVYDYFVNHRKIDLRYSGDLPC--INVGKP-KRPTYIPLEL
CSLVSLQRYTKALSTLQRSSLVEKSRQK-PQERMTVLSS------------------ALK
SSKYDAEPMLR-SCGISINPSFTQVEGRVLPAPKLK----VGNG--EDFFPR---NGRWN
FNNKKLVEPSRIEKW--AVVNFSARCD------IRNLVQNLTKCAEMKGIPIED-PF-DV
--------FEENPQSRRAPPVVRVEKMFEQIQSRLP--------GQPKFLLCLLPERKNS
DIYGPWKRKNLAEYGIVTQCI-APQRV------N--D----QYITNVLLKINAK------
----LGGLNSMLAVEHAPSLP--LVSK---VPTLILGMDVSHG----SPGQ-SDVPSIAA
VVSSRQWPLISRYRACVRTQSPKLEMIDSLFKRVSETED----EGIIRELLLDFYVTSGK
RKPDQIIIFR---DGVSESQFNQVLNIELDQIIEACKFL--DEKWSPTFVVIVAQKNHHT
KFFQPGS----------PDNVPPGTIIDNKVCHPRNNDFYLCAHA---------GMIGTT
RPTHYHVLLDEVGFSADDLQELVHSLSYVYQRSTTAISVVAPICYAHLAATQMGQFMKFE
-------------------D-TSETSSSHGGVTSAGAVPVPQLPRLQEKV----------
------------------------CN----------------------------------

-------------SMF-------------------------------FC-----------

>Ricinus-communis_29684.t000014_RICCO

-----------------------MDSFEPDGNGLREGNGIHEGNGSQ--EG---------

---------LPPPP----------PVVPPDVVPMRAEP----------------------
-----EPVK------------------------KKVVRVPIARRGLGSK-GQK-IS-LLT
NHFKVNVNKVD--DYFFHYCVSL-------------------------------------
----SYE-DGRPVDGKGVGRKVIDRVHETY----DSEMGGKKFAYDGEKSLFTVGA-LPR
NKLEFTVVLEDVTSNRNNG-------N--ASPDGHGSPNEGDRKRMRRPYQSKTFKVEIS
FA-AKIPMQAIANALRGQESE---NSQEAIRVLDIILRQHAAKQ-GCLLVRQNFFHNDPK
-----NFADVGGGVLGCR-------------------------G-------FHSS-----
-----------FRTTQGGLSLNIDV-STTMIIQPGPVVDFLIA---------NQNVRDPF
QLDWAKAKRTLKNLRIKASPSNQE---YKITGLSEMPCKEQT-----------------F
QLNQKGRDDNDP----LELTVYDYFVNHRRIELRYSGDLPC--INVGKP-KRPTFIPIEL
CSLVSLQRYTKALNTLQRASLVEKSRQK-PQERMSTLSN------------------ALK
SSNYDAEPMLR-SCGVSISTSFVQVDGRQLQAPKLK----VGNG--EDFFPR---NGRWN
FNNKKLVDPSKIERW--AVVNFSARCD------IRNLVRDLTKCAEMKGIPIEP-PF-DV
--------FEENPQFRRAPPTVRVEKMFDSIQSKLP--------GAPKFLLCLLPERKNS
DLYGPWKKKNLSDFGIVTQCI-APQRV------N--D----QYLTNVLLKINAK------
----LGGLNSMLAVEHSPSIP--LVSK---VPTIIIGMDVSHG----SPGH-SDVPSIAA
VVSSRQWPLISRYRACVRTQSPKVEMIDSLYKPVSDTED----EGMMRELLLDFYSSSGK
RKPEQIIIFR---DGVSESQFNQVLNIELNQIIEACKHL--DEKWNPKFVVIIAQKNHHT
KFFQPGL----------PDNVPPGTVIDNKVCHPRNNDFYLCAHA---------GMIGTT
RPTHYHVLLDEVGFSADELQELVHSLSYVYQRSTTAISVVAPVCYAHLAATQMGQFMKFE
-------------------D-ASETSSSHGGVTSAGAVPVPQMPKLSDKV----------
------------------------SS----------------------------------

-------------SMF-------------------------------FC-----------

>HbAGO4_8_9

-----------------------MDSYEPEKNGSQQD-----------------------

---------LPPPP----------TVVPPDVVPLQAEP----------------------
-----EPVK------------------------KKPLRVPIARRGLASK-GQK-IP-LLT
NHFKVNVTNVD--GYFFHYSVAL-------------------------------------
----SYE-DGRPVDGKGVGRKVIDRVQETY----DSELDGKHFAYDGEKSLFTIGS-LPR
NKLEFTVVLEDISSNRNNG---------KASPDGHGSPNESDRKRMRRPYQSKTFKVEIS
FA-AKIPMQAIANALRGQESE---NSQEAIRVLDIILRQHAAKQ-GCLLVRQNFFHNDPR
-----NFTDVGGGVLGCR-------------------------G-------FHSS-----
-----------FRTTQGGLSLNIDV-STTMIIQPGPVVDFLIA---------NQNARDPF
QLDWAKAKRTLKNLRIKASPSNQE---YKITGLSEKPCREQT-----------------F
QLKQKGDGEP------LELTVYEYFVNYRHIELRYSADLPC--INVGKP-KRPTYIPIEL
CTLVSLQRYTKALNTLQRASLVEKSRQK-PQERMTTLTN------------------ALK
SSKYDAEPMLR-SCGISISTSFAQVEGRVLPTPRLK----VGNG--EDFFPR---NGRWN
FNNKRLVEPCKIERW--AVVNFSARCD------VRNFVRDLTRCAEMKGIPIEP-PF-DV
--------FEESPQFRRAPPTVRVEKMFDQIQSKLP--------GAPKFLLCLLPERKNS
DIYGPWKKKNLADFGIVTQCS-APQRV------N--D----QYLTNLLLKINAK------
----LGGLNSLLAVEHTPSIP--LVSK---VPTIILGMDVSHG----SPGH-SDVPSIAA
VVSSRNWPLISRYRASVRTQSPKVEMIDSLYKRVSDTED----EGMIRELLLDFYNSSGK
RKPEQIIIFR---DGVSESQFNQVLNIELDQIIEACKFL--DEKWNPKFVVIVAQKNHHT
KFFQPGS----------PDNVPPGKALN-------ELSILL-------------NGRGTT
RPTHYHVLLDEVGFSADDLQDLVHSLSYVYQRSTTAISVVAPICYAHLAATQMGSFVKFE
-------------------E-TSETSSSHGGLTSAGAVPVPQLPRLQDKMRLLLSLSKFR
SPTTLSKSWKFILCESLILRIWLLST----------------------------------

-------------NIL-------------------------------FSSNA--------

>Ricinus-communis_29828.t000011_RICCO

-----------------------MEPPEEA-------------------EA---------

---------LPPPP----------PVVPADVVPIQLKP----------------------
-----EPDNVPE-----------TT-EKA----IKPKRVPMSRRGNGSR-GQR-IE-LLT
NHFKVGVNCDG--GHFSHYSVAL-------------------------------------
----FYE-DGRPVDSKGIGRKVIDKVRETY----DSDLAGKDFAYDGEKSLFTVGS-LPR
NKMEFTVLLDDVSSNRING-------S--GSPVGNGSPNGSEKKRMKRVFHSKTYKVEIS
FA-AKIPMQAIKAALRGQESE---NSQEAIRVLDIVLRQHAAKQ-GCLLVRQSFFHDDSR

-----NYVDLDGGVLGCR-------------------------G-------FHSS-----
-----------FRVSQGGLSLNIDG-STTTIIQPGPLIDFLLA---------NQHVSTPF
QIDWSKAKRTLKNLRIRVSPTNQE---YRITGLSENLCKDQI-----------------F
SMKSKGLNDGNCDDGMVDITVYEYFVNHRNIDLRYSGDLPC--INVGRP-KRPTFFPIEL
CSLLPLQRYTKALSVIQRSKLVESSRQK-PQEKMKILAD------------------VMK
SNNYGADPILR-SCGITISSQFTQLEGRVLTAPRLK----VGNG--EDLIPR---NARWT
FNNKKFAEPARIENW--AVVNFSARCD------IRGLCRDLCRVGEMKGIMISP-PE-HV
--------FEENPQFRHAPPPIRVEKMFEQIQPRFPD-------NPPRFLLSIFPDRKNS
DIYGPWKRKNLAEFGIFNQCLCSPNRL------S--E----MYVTNVLMKINAK------
----LGGLNTFLAVEQSRNVP--FVSK---VPTIIFGMDVSHG----SPGQ-SDVPSIAA
VVSSRNWPLLSRYRASVHSQSPKVEMIDSLFKP-----EGKDDDGIIRELLLDFYRSSGQ
TKPAQIIIFR---DGVSESQFNQVLNIELNQIIEACKFL--DESWSPKFTVIVAQKNHHT
KFFQLRS----------AENVPPGTVVDNGVCHPQSNDFYMCAHA---------GMIGTT
RPTHYHVLLDEIGFSADDLQELIHSLSYVYQRSTSAVSVVAPVRYAHLAATQIRLFMKFE
-------------------D-MSETSSSHGGLTTSGPTPVPELPVLHQKV----------
------------------------RS----------------------------------

-------------SMF-------------------------------FC-----------

>Populus-trichocarpa__POPTR_0006s02680_POPTR

-----------------------MESTEEPEALPPPPSDALPPPPP---DA---------

---------LPPPP----------PEIPPNVVPVQLTT----------------------
-----DSVPE-------------ET-KKI----SKPKRSPIARRGFGSR-GQK-IQ-LLS
NHFKVSI--SNTGGHFFHYCVSL-------------------------------------
----SYE-DGRPIDAKGIGRRLIDKVHETY----GSDLAGKDFAYDGEKSLFTIGA-LPR
NKMEFTVLLDSFSSNRNSG-------N--GSPVGNGSPNETDKKRMRRAFQSKTFKVEMS
FA-AKIPMQAIAAALRGQESE---NSQEALRVLDIILRQHAAKQ-GCLLVRQSFFHDDPK
-----NYVDLGGGVLGCR-------------------------G-------FHSS-----
-----------FRTSQGGLSLNIDG-STTTIIQPGPLIDFLIA---------NQNVSNPF
QIDWAKAKRTLKNLRIRVSPTNQE---YRITGLSENTCKEQM-----------------F
SLKSRASDGNDVES--VDITVYHYFVNHRSIDLRYSGDLPC--INVGKP-KRPTYIPVEL
CSLLPLQRYIKALTVLQRSQLVEKSRQK-PQEKIRILTD------------------VMK
SNNYAAEQMLR-SCGITISSQFTQVQGRVLTAPKLK----AGNG--EDVIPR---NGRWN
FNHKKFFEPSKIENW--AVVNFSARCD------VRGLVRDLIRFGEMKGILISD-PV-DV
--------VEENGQFRRAPPLVRVEKMFEQIQKAFPN-------APPRFLVCLLPDRKNS

DIYGPWKRKNLAEYGIFNQCL-APTRV------N--E----QYILNVLLKINAK------
----LGGLNSLLAMEQSRNIP--FVSK---VPTIIFGMDVSHG----SPGQ-SDMPSIAA
VVSSRNWPLLSRYRASVRSQSPKVEMVDSLFTLTPDKKD---DSGIVRELLLDYYRSSGQ
TKPAQIIIFR---DGVSESQFNQVLNIELDQIIEACKFL--DESWSPKFTVIVAQKNHHT
KFFQDGS----------PDNVPPGTVIDNAVCHPQSYDFYMCAHA---------GMIGTT
RPTHYHVLLDEIGFSADDLQELIHSLSYVYQRSTTAISVVAPVRYAHLAATQISQFLKCD
-------------------D-MSETSSSHGGLTSAGQTPVPELPELHRNV----------
------------------------CS----------------------------------

-------------SMF-------------------------------FC-----------

>Populus-trichocarpa__POPTR_0016s02480_POPTR

-----------------------MESNEEP----------EALAPPP--DA---------

---------LPPPP----------PEIPPNVVPVQLTT----------------------
-----GTFPE-------------ET-KKT----SKLKRSPITRRGVGSR-GQK-IQ-LVT
NHFKVSI--SNTGGHFFHYSVSL-------------------------------------
----YYE-DGRPVDAKGIGRRLIDKVHETY----GSDLAGKDFAYDGEKSLFTIGA-LPR
NKMEFTVLLDSFSSNRNSG-------N--GSPVGNGSPNETDKKRMRRAFQSKTFKVEMS
FA-AKIPMQAIAAALRGQESE---NSQEALRVLDIILRQHAAKQ-GCLLVRQSFFHNNPK
-----NYVDLGGGVLGCR-------------------------G-------FHSS-----
-----------FRALQGGLSLNMDG-STTTIIQPGPLIDFLIA---------NQNVSNPF
QIDWAKAKRTMKNLRIKVSPTNQE---YRITGLSENSCKEQM-----------------F
SLKSRAADGNDVES--FDITVYDYFVNHRSIDLRYSGDLPC--INVGKP-KRPTYIPVEL
CSLLSLQRYTKALTVHQRSQLVEKSRQK-PQEKIRILAD------------------VMK
SNNYAAEPMLR-SCGITISSQFTQVQGRVLPAPKLK----AGNG--EDVIPR---NGRWN
FNNKKFFEPSKIENW--AVVNFSARCD------VRGLVRDLIKFGEMKGILISD-PM-DV
--------LEENAQFRRAPPPVRVDKMFEQIQTAFPD-------APPRFLVCLLPDRKNS
DIYGPWKRKNLAEYGIFNQCL-APTRV------N--D----QYILNVLLKINAK------
----LGGLNSLLAMEQSRNIP--FVSK---VPTIIFGMDVSHG----SPGQ-SDIPSIAA
VVSSRNWPLLSRYRASVRSQSPKVEMVDSLFKLTADKKD---DCGIVRELLLDYYKSSGQ
TKPAQIIIFR---DGVSESQFNQVLNIELDQIIEACKFL--DESWSPKFTVIVAQKNHHT
KFFQDGS----------PDNVPPGTVIDNAVCHPQTYDFYMCAHA---------GMIGTT
RPTHYHVLLDEIGFSADDLQELIHSLSYVYQRSTTAISLVAPVRYAHLAATQISQFLKFD
-------------------D-MSETSSSHGGLTSAGQAPVPELPELHHNV----------
------------------------RS----------------------------------

-------------SMF-------------------------------FC-----------

>HbAGO6.1

-----------------------MEEAGG-------------------------------

---------SCPKP--
-----------------------------------TKRSIIARPGFGSS-GHP-MP-LLS
NHFKVSVNVTD--VVFYQYSVSI-------------------------------------
----TAE-DNRVV---DEKRKIIDRLCQTY----SCELAGKSFAYDGEKTLYSLVR-LPQ
SWMEFTVVPKESIAKRYVNFPSTSMICVCNADGGR--SFAVTCERSKRRLRSETFKVEIV
YA-AKIPLNSLALSFHGNETN--NNTRDALRVLDTILKNRQSR--------GVLMPRTPP
-----ARTPADGILRPIS-------------------------PDANIISIFVRG-----
-----------VAHLQSRPKSNLQV-AESLMKRDAIFISVLRPELGC-------------
-----MAESVLETELVKPRHRNKE---YKIIGLSQKVCKEQY-----------------F
SMKVRSHDCANGEPQVVKITVYEYFTKHCGIELTDSACLPC--LDVGNT-KQPIYLPIEL
CTLVPLQRYAKALFPTLRASLVASSRQN-PQDRKRTVID------------------AVR
NYRCDDDSLLS-ASGVSIERQMMQVDSRVLETPTLR----VGNK--EDVFPR---NGRWN
FNYKTLFKTTCIDRW--AVVNFSAPCY------TDKIFHDLISCGRRMGIQIES-PRTPI
---------WEDPKSRGDKPLDRVENMFEMLNRAKLL-------KDTQFILCVLPEKKSD
I-YGPWKKKCLCDFGIVTQCI-YPSTI------N--D----RYLTNVLLKINSK------
----LGGINSLLAIEDSMKIP--LIKD---TPTMILGMGVSLG----FPGR-SDIPSVAA
VVGSLYWPHISRYRASVRAQSPKEEMIDALYNPLANGKD----DGMMRKLLEDFCHTSNG
NKPKHIIVFR---NGVASHSSLMNFL-----VLQAYQDHFKSEVDIPKFTVIVAQKDRHT
KLFQDIS----------PENVPSGTVVDTEIVHPRNYDFYLCAHA---------SMTGTS
RPAHYHILLDEIGFSPDELQNLIHALSYVNQRSTTAIRTVAPLRYANLAARQMGQFMKFE
-------------------DNLSEISSGQG--------PVPELPRLHKNV---------
------------------------AS----------------------------------

-------------SMF-------------------------------FC-----------

>HbAGO6.2

-----------------------MEEAGG-------------------------------

---------SCPKP--
-----------------------------------TKRSIIGRPGFGSS-GHP-MQ-LLS
NHFKVSVNVTD--VVFYQYSVSI-------------------------------------
----TAE-DNRVV---DEKRKIIDRLCQTY----LYELSGKSFAYDGEKILYSLVR-LPQ
SWMEFTVVLEESIAKRYVNFPSTSMICVLDCNADGGGSFAATCKRSKRGLRSETFKVEIV
YA-AKIPLNSIALSLQRNETN--NNTQDALRVLDTILKNRQR---GCLL------SEEAK
-----NFTDGGGGITGVR-------------------------G-------YHSS-----
-----------FRTTQGGLSLNMDV-STTMFLTPGPVIDFLKA---------NQNVQDPC
YIDWVKAKIMLKNLRIKPRHRDME---YKIIGLSQKVCKEQY-----------------F
SMKVRSHDCANVKPQVVKITVYEYFTKHRGIELTDSACLPC--LDVGNT-KQPIYLPIEL
CSLVPLQRCTKALSPTLRASLVANSRQN-PQDRKRTVID------------------AVR
NYRCDDDSLLS-ASDVSIERQMMQVDGRVLGTPMLR----VGNK--EDFLPT---NGRWN
FNYKTLFKTTCIDRW--VVVNFSSRCY------NDKICHDFISCGRRMGIQIKS-PRTPI
---------WEDPQSRGDKPLDRVENMFEMLNRAKLL-------KDIQFILCVLPEKKSD
V-YGPWKKKCLSDCGIVTQCI-YPSTI------N--D----QYLSNVLLKINSK------
----LGGINSLLAIEDSIQIP--LIKD---TPTMILGMGVSLG----SPGP-SDKPSLAA
VVGSLYWPHISRYRASVRAQSPKEVMIDALYNPLANGKD----DGMMRELLEDFCHTSNG
IKPKHIIVFR---NGVAESQFSKVLNVEVEQILKAYQDLFKSEVDVPKFIVIVAQKDHHT
KLFQDIS----------PENVPSGTVVDTKIVHPRNYDFYLCAHA---------SMIGTS
RPAHYHVLLDEIGFSPDELQNLIHALSYVSQRSTTAIRIVAPLRYANLAARQMGQFMKFE
-------------------DNLSKICSGQG--------PVPKLPRLHKNV----------
------------------------AS----------------------------------

-------------SMF-------------------------------FC-----------

>Arabidopsis-thaliana_AT2G32940.1_ARATH

-----------------------METSSS-------------------------------

---------LPLSP----------ISIEPEQP----------------------------
---------------------------------SHRDYDITTRRGVGTT-GNP-IE-LCT

NHFNVSVRQPD--VVFYQYTVSI------------------------------------
----TTE-NGDAVDGTGISRKLMDQLFKTY----SSDLDGKRLAYDGEKTLYTVGP-LPQ
NEFDFLVIVEGSFSKRDCG-------------VSDGGSSSGTCKRSKRSFLPRSYKVQIH
YA-AEIPLKTVLGTQRGAYTP-DKSAQDALRVLDIVLRQQAAER-GCLLVRQAFFHSDGH
------PMKVGGGVIGIR------------------------G-------LHSS-----
-----------FRPTHGGLSLNIDV-STTMILEPGPVIEFLKA---------NQSVETPR
QIDWIKAAKMLKHMRVKATHRNME---FKIIGLSSKPCNQQL-----------------F
SMKIKDGEREVPI---REITVYDYFKQTYTEPISSA-YFPC--LDVGKP-DRPNYLPLEF
CNLVSLQRYTKPLSGRQRVLLVESSRQK-PLERIKTLND------------------AMH
TYCYDKDPFLA-GCGISIEKEMTQVEGRVLKPPMLK----FGKN--EDFQPC---NGRWN
FNNKMLLEPRAIKSW--AIVNFSFPCD------SSHISRELISCGMRKGIEIDR-PF-AL
--------VEEDPQYKKAGPVERVEKMIATMKLKFP--------DPPHFILCILPERKTS
DIYGPWKKICLTEEGIHTQCI-CPIKI------S--D----QYLTNVLLKINSK------
----LGGINSLLGIEYSYNIP--LINK---IPTLILGMDVSHG----PPGR-ADVPSVAA
VVGSKCWPLISRYRAAVRTQSPRLEMIDSLFQPIENTEKG--DNGIMNELFVEFYRTSRA
RKPKQIIIFR---DGVSESQFEQVLKIEVDQIIKAYQRL--GESDVPKFTVIVAQKNHHT
KLFQAKG----------PENVPAGTVVDTKIVHPTNYDFYMCAHA---------GKIGTS
RPAHYHVLLDEIGFSPDDLQNLIHSLSYVNQRSTTATSIVAPVRYAHLAAAQVAQFTKFE
-------------------G-ISEDGK------------VPELPRLHENV----------
------------------------EG---------------------------------

-------------NMF------------------------------FC-----------

>Populus-trichocarpa__POPTR_0014s15760_POPTR

--------------------------------------MISRRGVGTS-GRH-IS-LLT
NHFKVSVNVPD--AVFYQYNVSI------------------------------------
----TSE-DNRAVESKGIGRKLIDRLYQTY----SSEFAGKRFAYDGEKSLYTVGP-LPQ
NKSEFTVVLEESFAKHESG-----------SPGGGESPPAA-VKRSKRSYRSKTFKVETS
YA-AKIPLKSIALALKGIEID--NSTQDALRVLDIILRQQAANR-GCLLVRQSFFHDDSR
-----NFNDVGGGVTGVK------------------------G-------FHSS-----
-----------FRTTQGGLSLNMDV-STTMILTPGPVIDFLIV---------NQNVREPR
YVDWVKARRMLKNLRVKTKHNNME---FKIIGLSEKPCNQQY-----------------F
PMKLKNRDGANVEAQIVEVTVYDYFTKHCGIQLGYSAYLPC--LDVGKP-KRPNYLPLEL

CSLISLQRYKKALSSMQRASLVEKSRQK-PQERIKTVTE------------------AMR
SYCYDEDPVLS-SCGISIEKQMTQVDGRILETPKLK----VGNS--EDCIPP---NGRWN
FNNKTLLNPTSISKW--AIVNFSARCD------ISHVSRELINCGRRKGINIER-PHTLI
--------EEDQQSRR-GSPLARVERMFELIREKLP--------GPPEFILCVLAERKNS
DIYGPWKKTSLSDFGIVTQCI-SPTKI------N--D----QYLTNVLLKINSK------
----LGGINSLLAIEHSSHIP--LIMD---TPTMILGMDVSHG----SPGR-SDMPSVAA
VVGSRCWPLISRYRASVRTQSPKVEMIDALYKPLANGND----DGIIRELLVDFFQTSKG
HKPKQIIVFR---DGVSESQFNQVLNIELEQIIKAYQHL--GEVDIPKFTVIVAQKNHHT
KLFQAGGG---------TENVPPGTVVDTKIVHPRNYDFYMCAHA---------GMIGTS
RPAHYHVLLDEIGFSPDELLNLVHSLSYVYQRSTTAVSIVAPICYAHLAAAQIGQFMKFE
-------------------D-FSETSSGQRSMTSVGSTPVPELPRLHENV----------
------------------------EG----------------------------------

-------------SMF-------------------------------FC-----------

>CHLRE1

---MSGRGRGGGRGGGGGD------RGGGGYGGGGRGGGGGGGYGGGGGGGG--------

-----------------------RGGGGGGGYGGGGGRGGGGGGGGG--GV---------

---------VSPAQ----------AAASLDAILKSAKS----------------------
-----LTAPVKVETTA-----------------DGRPLGITRRPSAGSV-GKA-VA-LLA
NYFALATT-PAFPGQAYHYDVEIRSVETPGGGGGGGRGGGRGGGRGGGRGGRGGAAPAPA
TAEESAGAPAAEAGGEDLPPRLAHRVMASA--AAAHGWPAGAWRFDGRKNLFLPGELLPR
EVREWPVTLK---------------------------PREGDKSER-----DKGFVVTTK
WA-ACVGLTQLQDYLARRQQT---APRDAMQVLDIVIRHAFAIDPRCTVVGRGFYYGGEG
------VMPLGGGAEVWS-------------------------G-------FQQV-----
----------------------EF--------PMP-------------------------
--------------------GGRARR-KALVGLSEQGADRTM-----------------F
MNEKEG----------REMSVAEYFRST-GRPLRHP-GLPC--ANVGDR-RRAVFIPVEL
CTVVAGQRRMK-LDATQSAGMITAAKQD-PAVKKEACDKQ-----------------AKR
VAEALAAGGTDRCWGLKLATGMLPVQGRVLPNPVLQYGD--PQA--FDAGP----LGSWN
LRNVKFVDPRALDSWGVAVMMNQADVDFDGDNSLWQFLEDLTGGMIRCGMRVAS-PVTAA
STDSPPVEFGGMPGGGGRGGGRGIEATMRAAADAAAARYK----KPAQLVLVVLPEKTAD
E-YREVKRVSDIELGIPSQVV-VASKARVGYRAH--KGGGPQYCANVAMKINNK------
----LGGVNVQLSGGL-RNMP--VLGGAGAVPFMVLGADVTHPTGAAARAD-SRDPSVAA
VVASLD-ASLGRWASRVLLQAGRQEVITGM-------------CGATKELLLEFYRANKQ
VKPQRLVMYR---DGVSEGQFEQVLAEEYTALRRACREL--EEGYRPAITFVVVQKRHNT

RLLPSD---RAASDQ--KGNVVPGTVVDSGITAPDGFDFYLNSHA---------GLQGTN
KPAHYHVLVDEIGFGADGIQLLTYWLCYLYQRTTKSVSYCPPAYYADRAAFRGRTLLAAS
SSASDSAS-----------E-TASRSGRGAGAAEGGASAPPTFAGIHRNL----------
------------------------TN----------------------------------

-------------VLY-------------------------------FM-----------

>Arabidopsis-thaliana_AT1G31280.1_ARATH
---MER
GGYRGGRGDGRGRGGRGYGGGGGGGEQGRDRGYGGGEQGRGRGSERGGGNRGQGRG----
---EQQDFRSQSQRGPPPGH
GGRGTTQFQ--------------QPRPQVAPQPSQAPASYAGSVGGVAGRG---------

---------AWGRK----------PQVPSDSASPSTST----------------------
-TVVSEPVRVAEVMNL----------KPSVQVATSDRKEPMKRPDRGGVVAVR-RVNLYV
NHYKVNFN-PE--SVIRHYDVEI-------------------------------------
----KGEIPTK-----KVSRFELAMVRDKVFTDNPDEFPLAMTAYDGQKNIFSAVE-LPT
GSYKVEY-------------------------------------PKTEEMRGRSYTFTIK
QV-NVLKLGDLKEYMTGRSSF---NPRDVLQGMDVVMKEHPSK--CMITVGKSFFTRETE
-----PDEDFRFGVIAAK-------------------------G-------YRHT-----
-----------LKPTAQGLSLCLDY-SVLAFRKAMSVIEYLKLYFNW---------SDMR
QFRRRDVEEELIGLKVTVNHRKNKQK-LTIVGLSMQNTKDIK-----------------F
DLIDQEGNEPP-----RKTSIVEYFRIKYGRHIVHK-DIPC--LDLGKN-GRQNFVPMEF
CDLVEGQIYPKDNLDKDSALWLKKLSLVNPQQRQRNIDK------------------MIK
ARNGPSGGEIIGNFGLKVDTNMTPVEGRVLKAPSLKLAE-RGRV--VREEPNPRQNNQWN
LMKKGVTRGSIVKHW--AVLDFTASER--FNKMPNDFVDNLIDRCWRLGMQMEA-PIVYK
--------TSRMETL---SNGNAIEELLRSVIDEASRKHG---GARPTLVLCAMSRKDDG
--YKTLKWIAETKLGLVTQCF-LTGPATKG---G--D----QYRANLALKMNAK------
----VGGSNVELMDTFSF-----FKKE---DEVMFIGADVNHP----AARD-KMSPSIVA
VVGTLNWPEANRYAARVIAQPHRKEEIQGF-------------GDACLELVKAHVQATGK
R-PNKIVIFR---DGVSDAQFDMVLNVELLDVKLTFEKN----GYNPKITVIVAQKRHQT
RFFPATNNDGSD-----KGNVPSGTVVDTKVIHPYEYDFYLCSHH---------GGIGTS
KPTHYYTLWDELGFTSDQVQKLIFEMCFTFTRCTKPVSLVPPVYYADMVAFRGRMYHEAS
-SREKNFKQPR--------G-ASTSAASLASSLSSLTIEDKAIFKLHAEL----------
------------------------EN----------------------------------

-------------VMF-------------------------------FV-----------

>Arabidopsis-thaliana_AT1G31290.1_ARATH
--------------------------------------MDRGGYRGGRGDGRGRGGGGDR
GRGYSGRGDGRGRGGDGDRGYSGRGDGHGRGGGGDRGRGYSGRGDGRGRGGGGDRGRGYS
GRGDGHGRGGGGDRGRGYSGRGRGFVQDRDGGWVNPGQSSGGHVRGRGTQLQQPPPQEVP
PSSSQAQVS--------------QGVAPGDVGQGGVGDVGRDGVGDVGRDGVGDVGQGGV
GDVGQVGVGDVGQGGVGDVGQGGVGDVGRDGVGDVGRDGVGDVGRGGVGDRGQSQSGLSS
GHFGRGTQLQQPQP----------QAVSQSSSQGQVSQSFATGGVGLGAWARKPQLFSDS
TVLPSSSSSNVVASHTASGSQVMTP-KPS----SSDKKEPVKRPDKGGNIKVKGVINLSV
NHFRVSFS-TE--SVIRHYDVDI-------------------------------------
----KGENSSK-----KISRFELAMVKEKL-FKDNNDFPNAMTAYDGQKNIFSAVE-LPT
GSFKVDFSETE------------------------------------EIMRGRSYTFIIK
QV-KELKLLDLQAYIDGRSTF---IPRDVLQGMDVVMKEHPSK--RMITVGKRFFSTRLE
-------IDFGYGVGAAK-------------------------G-------FHHT-----
-----------LKPTVQGLSLCLNS-SLLAFRKAISVIEYLKLYFG------WRNIRQFK
NCRPDDVVQELIGLKVTVDHRKTKQK-FIIMGLSKDDTKDIK-----------------F
DFIDHAGNQPP-----RKISIVEYFKEKYGRDIDHK-DIPC--LNLGKK-GRENFVPMEF
CNLVEGQIFPKEKLYRDSAAWLKELSLVTPQQRLENINK------------------MIK
SSDGPRGGDIIGNFGLRVDPNMTTVEGRVLEAPTLKLTDRRGNPIHEKLMSE---SNQWN
LTTKGVTKGSIIKHW--AVLDFTASES-LKKKMPGYFVNKLIERCKGLGMQMEA-PIVCK
--------TSSMETL---YDGNALEELLRSVIDEASHNHG---GACPTLVLCAMTGKHDG
--YKTLKWIAETKLGLVTQCF-LTISAIKGETVS--D----QYLANLALKINAK------
----VGGTNVELVDNIFSF----FKKE---DKVMFIGADVNHP----AAHD-NMSPSIVA
VVGTLNWPEANRYAARVKAQSHRKEEIQGF-------------GETCWELIEAHSQAPEK
R-PNKIVIFR---DGVSDGQFDMVLNVELQNVKDVFAKV----GYNPQITVIVAQKRHQT
RFFPATTSKDGRA----KGNVPSGTVVDTTIIHPFEYDFYLCSQH---------GAIGTS
KPTHYYVLSDEIGFNSNQIQKLIFDLCFTFTRCTKPVALVPPVSYADKAASRGRVYYEAS
-LMKKNSKQSR--------G-ASSSSASVASSSSSVTMEDKEIFKVHAGI----------
------------------------EN----------------------------------

-------------FMF------------------------------FV------------

>HbAGO2_3.1
MVMELKEQASREKFSCLDAVTAEL----------------HHWTMELITSKDRGLQLSDL
GIGMASGEASKENRKRKREISRKVWEMKRAEEMQTPRHEPGGIQEGSSQPSGPEVVEMVR
SPPRREEPPPPPPVVPG--TEGGPSQQQREGYSHRGRGDRGRGGSGGVGKGQQWIPTTQG

RGGAQGRGGAQGRGGSAWLTR--GASSSGGGGGGGERGGSTSTRGRG--PT---------

---------TPAPP----------HTVPRRSDVPESEP----------------------
-----TLSGTSIRPVNISEHLASPS-SPSD---TTTRVVPVKRPDKGGTLAIR-TASLRV
NHFPVRFN-PE--SIIRHYDIDV-------------------------------------
----KPVVPPKNGRAVKISKSDLTVIRNKLFTDDSSQFPLSMTAYDGEKNIFSAVP-LPT
GKIKVEFS-----------------------------EGED--------MKDRAYTFNIK
LV-NELKLCKLKDYLTGKLFS---IPRDILQGMDVVMKENPAR--HMVSVGRSFHSTEAY
------KEDLGFGITASR-------------------------G-------FQHS-----
-----------LKPTFQGLAMCLDY-SVLAFRKRLPVIDFLREHV-------PGFNLNDF
RSCGRDVENALKGLKVTVTHRITKQK-YTIAGLTKDNARYLS-----------------F
FSEDPNSKAPP-----RKVILVEYFRQKYQ-DIQFK-DIPC--LDLGTN-NRKNYVPMEF
CVLVEGQIYPKENLNRDVGLYLKKISLATPRDRQRIICD------------------MVA
DRDGPCGGNIIQNFGMEVDMDMTSVLGRVIGPPELKLSASNGRV--IKIAVD-KEKCHWN
LVGKGVVEGKPIDRW--VVIDFSSSERGRYKLRPEQFIPKLRARCENLGISMKE-PLFCH
--------PANMHKF---SSADLLRDLLEVVNDRAYKICR----GQLQIIICVMSRKDSG
--YKYLKWVSETRVGVVTQCC-LSDHVNNG---N--D----QYFANLALKINAK------
----LGGSNVELIDRLPF-----FEGE---GHVMFIGADVNHP----GARN-KTSPSIAA
VVATVNWPAANRYAARVRPQYHRKEKILNF-------------GDMCFELVETYVRLNRV
K-PGNIVIFR---DGVSEGQFDMVLNEELTDLRRAFKSS----NYAPTITLIVAQKRHQT
RLFPGNRSSGSS-----NGNVSPGTVVDTKIVHPFEFDFYLCSHYGSLGTSNHYGSLGTS
KPTHYHVLWDEHGFSSDQLQKLIYNMCYTFARCTKPVSLVPPVYYADLVAYRGRLYYEAI
-M--DGQSPAS--------A-SSSTSSAATSSLSSAASLDDRFYKLHAEL----------
------------------------EN----------------------------------

-------------IMF-------------------------------FV-----------

>Populus-trichocarpa__POPTR_0012s11920_POPTR

---------MPKGL----------ASSSSD------------------------------
---------------------------------NANRVSPVQRPDTGGKLAVR-TPRLLV
NHFPVKFS-PK--SIIRHYDVDI-------------------------------------
----KQEVPPKHGRPGKISKSILTMIRDKLFTDDPSRFPLGKTAYDREKNIFSAVP-LPT
GTFRVEVS-----------------------------EAEDAK--------PRSYLFTIK
LV-NELQLRKLKDYLDGTLRS---VPRDILQGMDVVVKEHPAR--TMISVGRGFHSVRAH

------QDYLGYGIIASK-------------------------G-------CQHS-----
-----------LKPTSQGLALCLDY-SVLSFHEPVSVIDFLTKHI-------CGFNLNNF
RRCRGDVEIALKGLKVRVTHRVTKQK-YVIVGLTRDDTRDIT-----------------F
SQEDPDGKAS------QNVRLVDYFRQKYGRDIVHQ-DIPC--LEM-KS-NMRNYVPMEY
CVLVEGQVFPKEHLQRDEAQMLKDISLAKAKDRQKTICS------------------MVR
DGDGPFGGEIIRNFGMEVSVDMTPVVGRVIGPPELKLGAPNGRV--MKV-PV-DEKCQWN
LVGKGVVEGKPIERW--AVLDFSSDDY-QCPLNADHFIPKLIARCLKLGIRMEE-PLFYE
--------PTSMRLF-SNSNVDRLRELLERVNGRARKISR----GQLQFLLCVMSKKDPG
--YKYLKWICETKVGIVTQCC-LSRSANKV---N--D----QYLANIGLKINAK------
----LGGSNAELSDRLPY-----FGDE---NHIMFIGADVNHP----AARN-TTSPSIAA
VVGTTNWPAANRYAARVRPQDHRCEKILNF-------------GDMCLELVEFYSRLNKA
K-PEKIVIFR---DGVSEGQFDMVLNDELMDIKRAFRSI----MYTPTITLIVAQKRHQT
RLFLEDGGR--------IGNVSPGTVVDTKIVHPFEYDFYLCSHY---------GSLGTS
KPTHYHVLWDEHGLSSDQLQKLIYDMCFTFARCTKPVSLVPPVYYADLVAYRGRLYHEAV
-------------------MEGQSPSSVSSSSSSRTSSSLSVGASLEERFSS--------
------------------------SS----------------------------------

-------------TVH-------------------------------SS-----------

>Populus-trichocarpa__POPTR_0015s15780_POPTR

-----------------------MKSGQLQIEQLDAEYRSIPRFNNF--LW---------

---------QQLDT----------LNLSYVGNINGISS----------------------
-----LFFAGPNVPK--------GL-ASV----TANRISPVLRPDKGGKLAVR-TPRLLV
NHFLVKFN-PK--SIIRHYDVNI-------------------------------------
----KQEVLPKHGRPGKISKSNLAMIRDKLFADDPSSFPRAMTAYDGEKNIFSAVS-LPT
GTFKVQVSE----------------------------AEDA---------KPRSYLFTIK
LV-NELELHKLKDYLDGKLRS---TPRDILQGMDVVVKEHPAR--TMISVSRSFHSVRDH
------QIHLGHGIIASR-------------------------G-------CQHS-----
-----------LKPTSQGIALCLDY-SVLSFHEPLSVIEFLTKHI-------SGFNLNNF
RSFRRAVEGVLKGLKVRVTHRVTKQK-YVITGLTRDDAQYIT-----------------F
PQEDPDGKAS------QNVRLVEYFRQKYHRDIVHQ-DIPC--LEM-KS-KMKNYVPMEF
CVLVEGQVFPKEYLKETEAKMLKKFSLANPKDRQKTICR------------------MVQ
DGDGPCSGEIIRNFGIEVSKNMTSLIGRAL----------DGRV--IKIPVD-KEKCQWN
LVGKGV----------------------------------LIARCQSLGIRMEE-PL---
--------FYQPTSMRKFSNVDVLREQLETVNERAHKSCG----GQLQFLLCVMSRKDPG

--YKYLKWISETKVGIVTQCC-LSTPANEG---K--D----QYLANLGLKINAK------
----LGGSNAELSGRLPY-----FGNE---NRVMFIGADVNHP----GAQN-KTSPSIAA
VVGTINWPAANRYAARVRPQYHRKEQILNF-------------GDMCLELVECYSRLNKA
K-PEKIVIFR---DGVSEGQFDMVLNEELTDIMKAFKSI----NYTPTITLIVAQKRHQT
RLFPGDEGS--------TGNVSPGTVVDTTIVHPFEYDFYLCSHY---------GSLGTS
KPTHYYVLWDEHGLSSDDLQRLIYNLCFTFARCTKPVSLVPPVYYADLVAYRGRLYHE--
---------------------AVMEGQSPSSSSSRTSSSLSTAASLEEX-----------

>Ricinus-communis_30093.t000002_RICCO
--MESGGGGFMGGK
AATGGGRAAAGRGRGRGMNNDNSGCTGGGRGGYQYRTHYHQQQHGQSGVSSQ--------
---DNSSYGGRGGAGGYGCR
GGDGAGR----------------GGGGAGGRFSGSSGRGGRGRRGSSRGSA---------

---------SSQDI----------AAINPEHTVQQMQP----------------------
-------------------------------------LNLSRQDNSSGKNNIR-TISLRV
NHFLLSFD-PE--SIIRHYDFSI-------------------------------------
----KPDVPARNSLPMKVPKTILSMIRNKLFSDDPTRFPLSMTVYDGEKNIFSTVS-LPT
GKFKVELS-----------------------------KNEGIR--------IRSFMVELQ
LV-NELKCDKLNDYLRGRVVS---VPREVLQALDVVMKENPMR--QMIYAGRIFHPIMPY
-----PGDDLRRGITASR-------------------------G-------IKHT-----
-----------LKPTSQGLALCLDY-SVLPLLKQMPVIDFLKEHI-------RGFNLNNF
RAFRREVERVLKELKVTVNHRTTGQK-FKIAGLTHDDTQDIS-----------------F
EVDRISE---------RKVWLVDYFKEKYNKNITHR-NIPC--LDLGKK-NRTNYVPMEF
CSIAKGQRFAMEDLDRNQSEKLRRISLASPKSREGMICD------------------MIQ
SSDGPCGGDISQNFGIGTDLNMTKVTGRVLAPPELKLGNSGGRP--TAVDRD---KCHWN
LFKKSVVHSKPIRLW--GVLNFGSNDL-------EKFIPELISNSEKLGIHMDE-PLFCL
--------HHPMNLL---HNVDNLQQLLESVNNECYKRNG---GEYLQILVCVLPKEDPG
--YSNLKWICETKVGIVTQCC-LSENAFRP---K--A----QFLANLALKINAK------
----LGGSNVELFKQPQC-----LQSK---GHVMFIGADVNHP----SSYN-STSPSIAA
VVATMNWPAANQYGALICPQDHRAEKILKF-------------GDMCLELVNAYARLNQV
R-PENIVVFR---DGVSESQFDMVLNEELKDIKAAFESL----EYFPTITLIVAQKRHTT
RLFLDSDE---------DENVPPGTVVDTVITSPSGSDIYLCSHF---------GQIGTS
KPAHYQVLQNEIEFTPNELQEFIYSICFTSAQCTKPVSLVPPVIYADRAAFRGRLYYNAM
-------------------E-LHQPSAPSTSSSSLASFDEQPF-RLHPNL----------
------------------------EN----------------------------------

-------------SMF------------------------------YI----------

>HbAGO2_3.2
--------------MSDQSFNVVVDRARQIEISYTVDNSGRAKKNRAEGSSGVPYMATM
DSGGQSHYRGRANKKGGFKHKFRGFRPGYGSNSGHSSGYSSSGSGSRSSLAPCTQCGRGH
SGPCMMGSGRYSAGRGRGTGGGRSGEDGGRGRGRGRGRGRGGDHQHRSRHQQQQILVP
GQGGRTQTQWQSSPGQGGRGSGLVGPARGGHGGIGGAGGGRGDWGPAGGRF---------

---------QGPSS----------SAVPVHSRIEQQES----------------------
-----SCEKLSAMQSI-------KI-STSSPLEVAGKLVPIKRPDHGGKNDIG-GRRLCV
NHFLLNYN-SG--GIIRHYDVDV-------------------------------------
----KPDLPPKNVQAMKVPKAVLSLIRNKLFSDDPDKFPMSMTAYDGEKNIFSAVP-LPT
GTFKVELS-----------------------------NEEGMN--------IRHFTVAVQ
LV-NELKCTKLDDYISGKCLS---IPRDVLQALDVVMKENPAR--QMIYACRSFHPTRPD
-----PRDDLGHGITVSR-------------------------G-------IKYS-----
-----------LKPTAQGLALCLDY-SVFPFCKQIPVIDFLKEQI-------PEFNPNNF
TRFRKQVETVLKGLKVTVTHRTTNQK-YKIAGLSDENTQDIS-----------------F
DIENPNDQTPL-----RKVSVVSYYKEKYNKDIMHK-NIPS--LDLGKKSNRKNYVPMEF
CMIAGGQRYAKELLDKKLSENLRKISLASPKVRENKIYD------------------MVH
DRDGPCSGDITQNFGIGVDVQMTRVTGRVIEPPELKLRTSNGRW--TMATLD-RVKCDWN
LRRNSVISSKPIRLW--GVLDFGSFS-------IEKAIPELISRSERLGIHMGQ-PLFYK
--------RLQMNLLY---DVDNLHQLLESINSESYKIGG----THLQILVCVMPREDPG
--YNNLKRISETKVGILTQCC-LTKNCNRA---NK-D----QFLANIAIKINAK------
----LGGSNVELSKQPQC-----LQSK---GHVMFVGADVNHP----GSYN-LTSPSIAA
VVGTMNWPAANQYIARICPQYHRVEKILKF-------------GGMCLELVNTYNRLNQA
R-PEKIVLFR---DGVSDGQFDMVLNEELMDLKMTFEAL----NYFPTITVVVAQKRHMT
RLFVVGDM---------DENVPPGIVVDTKITHPFGFDFYLCSHY---------GHIGTS
KPAHYHVLWDENGFTSDELQELIYSMCFTCAQCTKPVSLVPPVWYADRAAYRGRLYHDSI
EWYQP--------------S-ASPSSSSSPPSRSSTTSFDEQLYKLHPNL----------
------------------------EN---------------------------------

-------------SMF------------------------------FI----------

>Arabidopsis-thaliana_AT1G69440.1_ARATH
--MEEKTH
HHHHSTNKHIPSSKSRTPLLHKPYHHHVQTNPPPFLLHPSSHQNLNLVASNLPSSYYYYY
Y--CYFYSQFHNSLPPPPPP
HLLP-------------------LSPPLPPLLPLPPPHSMTRFHKSL-------------

-----------PVS----------QVVERKQQHQQKKK----------------------
-----IQVSNNKVS---------GS-IAI----EEAALVVAKRPDFGGQDGSV-IY-LLA
NHFLVKFD-SS--QRIYHYNVEI-------------------------------------
----SPQ-PSK-----EIARMIKQKLVETD----RNSFSGVVPAFDGRQNIYSPVE-FQG
DRLEFFVNLPIPSCKAVMN------------------YGDL-REKQPQKKIEKLFRVNMK
LV-SKFDGKEQRK--EGE--DWAPLPPEYIHALDVILRENPME--KCTSIGRSFYSSSMG
-----GSKEIGGGAVGLR--------------------------G-------FFQS-----
-----------LRHTQQGLALNMDL-SITAFHESIGVIAYLQKRLEFLTDLPRNKGRELS
LEEKREVEKALKNIRVFVCHRETVQR-YRVYGLTEEITENIW-----------------F
PDREG-----------KYLRLMSYFKDHYGYEIQFK-NLPC--LQISR--ARPCYLPMEL
CMICEGQKFLGKLSDDQAAKIMKMGCQK-PNERKAIIDK------------------VMT
GSVGPSSGNQTREFNLEVSREMTLLKGRILQPPKLK----LDRP--------------RN
LKESKVFKGTRIERW--ALMSIGGSSD--QKSTIPKFINELTQKCEHLGVFLSKNTLSST
--------FF-EPSHI-LNNISLLESKLKEIQRAAS--------NNLQLIICVMEKKHKG
--YGDLKRISETRIGVVTQCC-LYPNITKL---S--S----QFVSNLALKINAK------
----IGGSMTELYNSIPSHIPRLLRPD---EPVIFMGADVTHP----HPFD-DCSPSVAA
VVGSINWPEANRYVSRMRSQTHRQEIIQDL-------------DLMVKELLDDFYKAVKK
L-PNRIIFFR---DGVSETQFKKVLQEELQSIKTACSKF---QDYNPSITFAVVQKRHHT
RLFRCDPD---------HENIPPGTVVDTVITHPKEFDFYLCSHL---------GVKGTS
RPTHYHILWDENEFTSDELQRLVYNLCYTFVRCTKPISIVPPAYYAHLAAYRGRLYIERS
-S-----------------E-SNGGSMNPSSVSRVGPPKTIPLPKLSDNV----------
------------------------KN----------------------------------

-------------LMF-------------------------------YC-----------

>Populus-trichocarpa__POPTR_0010s17100_POPTR
---MEETEESSG
NKKCTTKTRTFRGRTNTHKHHYHQYQYQYQYHYHYQHQLLQYSNQYGFFNHN--------
---QYPGYYPALLPLPPPIP
LQLA-------------------LTPPLPQNHSFISKTQLQKPLCKLNNPP---------

---------PPPPS----------PAPPTSSDTKGPAV----------------------
-----TTSPAFEGLQQQKNRPLKGDGGRKVMSATTQSLVVARRPDSGGVEGSV-IT-LLA

NHFPVQFD-SS--QRIFHYNVEI------------------------------------
----SPN-PSR-----EVARMIKQKLVKEN----SAVLSGALPAYDGRKSLYSPVE-FQK
DRLEFYVSLPIPTTKSSL-------------------PFGEFNFLQEKHQQLKLFRINIK
LV-SKLDGKELSRYLSKEGDDWIPLPQDYLHALDVVLRESPME--RCLPVGRSLYSSSMG
-----GTKEIGGGAVALR-------------------------G-------FFQS-----
-----------LRPTQQGLALNVDF-SVTAFHESIGVIPYLQKRLEFLRDLPQRKKRSLV
GEERKEVEKALKNIRIFVCHRETVQR-YRVFGLTEEATENLW-----------------F
SDRDG-----------KNLRLLNYFKDHYNYDIQFR-NLPC--LQISR--SKPCYLPMEL
CMICEGQKFLGKLSDDQTARILKMGCQR-PKERKAIIDG------------------VMR
GSVGPTSGSQGREFKLHISREMTRLSGRILQPPKLRLGD-GGHV--RDLIPS-RHDCQWN
LLDSHVFEGTRIQRW--ALISFGGTLD--QKSSIPKFINQLSQRCEQLGIFLNKNTMIKP
--------QY-EPTQV-LNNVSLLESKLKKIHSAAS--------NNLQLLICVMEKKHKG
--YADLKRIAETSVGVVTQCC-LYLNLGKL---S--S----QFLANLALKINAK------
----VGGCTVALYNSLPSQIPRLLRSN---EPVIFMGADVTHP----HPLD-DISPSVAA
VVGSMNWPAANKYVSRMRSQTHRQEIIQDL-------------GEMVKELLDDFYQELNE
L-PKRIIFFR---DGVSETQFYKVLKEELQAIREACSRF---PGYRPPITFAVVQKRHHT
RLFPNETDPSSTQNQFSDENIPPGTVVDTVITHPREFDFYLCSHW---------GVKGTS
RPTHYHVLWDENQFTSDELQKLVYNLCYTFVRCTKPVSLVPPAYYAHLAAYRGRLYLERS
-------------------E-CMASIRNASTISRAAPPKAAPLPKLSENL----------
------------------------KK----------------------------------

-------------LMF-------------------------------YC-----------

>Ricinus-communis_29813.t000096_RICCO
--MEET
RESNANKKCTSKPRTLRGRTNTHKHQYQYQYQYQYQHHFLQYSNQFGFFNHSSN------
---LYPSYYPALLPLPPPIP
LQLA-------------------LNPPFPQNHSFGSKTHFQKPSCKLN------------

---------NPPRP----------TSSATPEPLLSISS---------------------
-----APERLQPRKSLPLKRNDRRKGVGS----TTQALVVARRPDSGGVEGPV-IT-LLA
NHFLVQFN-PS--QKIFHYNVEI------------------------------------
----SPN-PSR-----EVARMIKQKLVDEN----SAVLSGAFPAYDGRKNLYSPVE-FQN
DRFEVYISLPIPTSKSSL-------------------PLGELNDFQEKHQQLKLFRLNIK
LV-SKLDGKELASYLSKESDDWIPLPQDYLHALDVVLRESPME--KCIPVGRSFYSSSMG
-----GTKEIGGGAVGLR-------------------------G-------FFQS-----
-----------LRPTQQGLALNVDF-SVTAFHESIGVIAYLQKRLDFLWDLPQNKRRSLI
GEERKEVEKALKNIRVFVCHRETVQR-YRVYGLTEQATENLW-----------------F
ADRDG-----------KNLRLLSYFKDHYNYDIKFR-NLPC--LQISR--SKPCYLPMEL

CMICEGQKFLGKLSDDQTARILKMGCQR-PKERKAIINE------------------VMR
GSVGPTSGNKDREFKLHVSREMTKLKGRILQPPKLRLGN-GGSK--RDLIPS-RHDRQWN
LLDSHVLEGTRIERW--ALMSFGGTPE--QKSNIPKFINQLSQRCEQLGIFLNKNTIISP
--------QY-EPTQV-LNNVSLLESKLKKIHKAAS--------NNLQLLICIMEKRHKG
--YADLKRIAETSVGVVSQCC-LFPNLGKL---S--S----QFLANLALKINAK------
----VGGCTVALFNSLPSQIPRLLHSD---DPVIFMGADVTHP----HPLD-DFSPSVAA
VVGSMNWPAANKYASRMRSQTHRQEIIQDL-------------GAMVKELLDDFFQEVGK
L-PKRIIFFR---DGVSETQFHKVLQEELQAIREACSRF---PGYRPPITFAVVQKRHHT
RLFPCETDLASIQNQFYDENIPPGTVVDTVITHPKEFDFYLCSHW---------GVKGTS
RPTHYHVLWDENQFTSDELQKLVYNLCYTFVRCTKPVSLVPPAYYAHLAAYRGRLYLERS
-------------------E-SMTSARNASAVSRAAPPKATPLPKLSENV----------
------------------------KN----------------------------------

-------------LMF-------------------------------YC-----------

>HbAGO7
---MEDSE
ESNAGKKCTTKTRTFRGRTNSTHKHQ----YQYQYQHHLFRYSNQFGFFNHN--------
---QYPTYYPALLPLPPPIP
LQLA-------------------LTPPFPQNHSSRSKTHLQKPSCKLNNHP---------

---------PPPCP----------NSSVSQGPVVTISS----------------------
-----APEGLQRRKSTPVKGNDGKKAMGS----PAQSLVAARRPDSGGVEGPV-IT-LLA
NHFLVQFN-SS--QRIFHYNVEI-------------------------------------
----SPN-PSK-----QVARLIKQKLVEDN----SAVLSGAFPAYDGRKNFYSPVE-FQN
DRFEFYISLPIPTSKSSL-------------------PFREPKDSQERHQQLKLFRINIK
LV-SKLDGKELSRYLSKEGDDWIPLPQDYLHALDVVLRESPME--KCIPVGRSFYSSLMG
-----GTTEIGGGAVGLR-------------------------G-------FFQS-----
-----------LRPTQQGLALNVDF-SMTAFHESIGVIPYLQKRLKFLRDLPQNKARGLI
NEEMKDVDKALKNIRVFVCHRETVQK-YRVYGLTEETTENLW-----------------F
ADRDG-----------KNLRLVSYFKDHYNYDIKFR-NLPC--LQISR--SKPCYLPMEL
CMICEGQKFLGKLSDDQTARILKMGCQR-PKERKNIIHE------------------VMR
GSVGPTSGNQSREFKLNVSREMTRLNGRILQPPKLRLGD-GGLI--RDLVPS-RYDCQWN
LLDSHVFEGKRIERW--ALISFGGTSD--QKSNIPKFINQLSQRCEHLGIFLSKSTIISP
--------QY-EPTQV-LNNVALLESKLKKIHKAAS--------NNLQLLICIMEKRHKG
--YADLKRIAETNVGVVSQCC-LFANLGKL---S--S----QFLANLSLKINAK------
----VGGCTVALYNSLPSQIPRLLHSD---EPAIFMGADVTHP----HPLD-DFSPSVAA
VVGSMNWPAANKYASRMRSQTHRQEIIQDL-------------GAMVKELLDDFYQEVGK
L-PKRIIFFR---DGVSETQFYKVLHEELQAIREACSSV---PGYRPLITFAVVQKRHHT

RLFPCETDISFIQNQFYNENIPPGTVVDTVITHPKEFDFYLCSHW---------GVKGTS
RPTHYHILWDESQFTSDELQKLVYNLCYTFVRCTKPVSLVPPAYYAHLAAYRGRLYIERS
-------------------E-SMASKRNVCTISRAAPPKATPLPKLTTTFPPFPNHQRLT
SNPPHCQPPPQDTGKRVETPSYAQRD----------------------------------

-------------FLT-------------------------------F------------

>Ricinus-communis_29807.t000009_RICCO

--MPLMQMKDFEES
YMVVSMQS---------------LQTCKNLEIPFSNGDDNSETAKES--LG---------

---------RKRAN----------GRRSRGGKGLKVES----------------------
-----KKILFQDYKLDSSE----ES-SPS----SCKSLMFHRRPGHGQL-GTK-CI-VKA
NHFLAQM--PD--SDLSHYSVEI-------------------------------------
----KPEVTSR-----KLSKAIMTQLVKMH---RETDLGTRLPVYDGGRNLYTARS-LPF
TSKDFTITLVH--------------------------EDEA-TGNIK----KRDFEVTIK
FE-ALAGMLQLRELLSGKPVD---TPQEAITVIDIVLRELAAQ--RYVSIGRSFYSPDIK
-----KPQQLEGGLESWR-------------------------G-------FYQS-----
-----------IRPTQMGLSLNIDM-SATAFIEPLLVIEFVAQILNK-----DVYSRPLS
DADRVKVKKALRGVKVEVTHRRNVRRKYRISGLTTQPTRELI-----------------F
PLDEHM-----------NMKSVVEYFQEMYDTIQYP-HLPC--LQVGNQ-RKVNYLPMEA
CKIVRGQRYTKGLNEKQITSLLKVSCQR-PRDQEMDILQ------------------TIH
QNGYEHDPYAK-EFGISIDSKLASIDARVLPAPWLKYSD-TGKV--KEYLPQ---VGQWN
MMNKKVINGSIVRYW--ACINFSRSVQ---ETTARSFCQQLVQMCRISGMDFNGEPVIPI
--------YAARPDQ--------VKKALKYVYHAAAKKLE---GKELELLIAILPDSNGS
L-YGDLKRICETDLGLISQCC-LTKHVFKI---N--R----QYLANVSLKINVK------
----MGGRNTVLLDAISWRIP--LVSD---IPTIIFGADVTHP----ESGE-DISPSIAA
VVASQDWPEVTKYAGLVCAQPHRQELIQDLFKTWQDPQQGTVAGGMIRELLLSFKKATGQ
K-PLRIIFYR---DGVSEGQFYQVLLYELDAIRKACASL--EPSYQPPVTFVIVQKRHHT
RLFASNHNDRSSIDR--SGNILPGTVVDTKICHPTEFDFYLCSHA---------GIQGTS
RPAHYHVLWDENNFTADEIQSLTNNLCYTYARCTRSVSVVPPAYYAHLAAYRARFYMEPD
---------------------ASENPKICRTLTANGSC-VRPLPALKEKV----------
------------------------KN----------------------------------

-------------VMF-------------------------------YC-----------

>Arabidopsis-thaliana_AT5G43810.1_ARATH

---------MPIRQMKDSSETHLVIKTQPLKHHNPKTVQNGKIPPPSPSPVTVTTP----
---ATVTQSQASSPSPPSKN
RSRRRNRGG--------------RKSDQGDVCMRPSSRPR--------------------

---------KPPPP----------SQTTSSAVSVATAG----------------------
-----EIVAVNHQMQMG----------------VRKNSNFAPRPGFGTL-GTK-CI-VKA
NHFLADL--PT--KDLNQYDVTI-------------------------------------
----TPEVSSK-----SVNRAIIAELVRLY---KESDLGRRLPAYDGRKSLYTAGE-LPF
TWKEFSVKIVD--------------------------EDDGIINGPKR---ERSYKVAIK
FV-ARANMHHLGEFLAGKRAD---CPQEAVQILDIVLRELSVK--RFCPVGRSFFSPDIK
-----TPQRLGEGLESWC-------------------------G-------FYQS-----
-----------IRPTQMGLSLNIDM-ASAAFIEPLPVIEFVAQLLGK-----DVLSKPLS
DSDRVKIKKGLRGVKVEVTHRANVRRKYRVAGLTTQPTRELM-----------------F
PVDENC----------TMKSVIEYFQEMYGFTIQHT-HLPC--LQVGNQ-KKASYLPMEA
CKIVEGQRYTKRLNEKQITALLKVTCQR-PRDRENDILR------------------TVQ
HNAYDQDPYAK-EFGMNISEKLASVEARILPAPWLKYHE-NGKE--KDCLPQ---VGQWN
MMNKKMINGMTVSRW--ACVNFSRSVQ---ENVARGFCNELGQMCEVSGMEFNPEPVIPI
--------YSARPDQ--------VEKALKHVYHTSMNKTK---GKELELLLAILPDNNGS
L-YGDLKRICETELGLISQCC-LTKHVFKI---S--K----QYLANVSLKINVK------
----MGGRNTVLVDAISCRIP--LVSD---IPTIIFGADVTHP----ENGE-ESSPSIAA
VVASQDWPEVTKYAGLVCAQAHRQELIQDLYKTWQDPVRGTVSGGMIRDLLISFRKATGQ
K-PLRIIFYR---DGVSEGQFYQVLLYELDAIRKACASL--EPNYQPPVTFIVVQKRHHT
RLFANNHRDKNSTDR--SGNILPGTVVDTKICHPTEFDFYLCSHA---------GIQGTS
RPAHYHVLWDENNFTADGIQSLTNNLCYTYARCTRSVSIVPPAYYAHLAAFRARFYLEPE
IMQD-----------------NGSPGKKNTKTTTVGDVGVKPLPALKENV----------
------------------------KR----------------------------------

-------------VMF-------------------------------YC-----------

>HbAGO10
------------------MVLIPSVVRTDTSHRNTLELERIELETAPEKKKSEKTEKEGK
SVEQSSSGPTGKRKNHGGHNRGGKKSSRGRYSGQKPPLSGQQSTRSSYPPHQCETCGR--
---NHGGVCYKAMGACYNCG

GIGHFA-----------------KDCTSTRRVGPLPATAEGSVQSPVTRGS---------

---------QPPSRGIGRGRGNPKDTLPKTAPVLAELD----------------------
--HFLLRLKGQFRALSPEVHNHLAEVLPI----LIKSLSYAPRPSYGQL-GTK-CI-VKA
NHFFAEL--PD--KDLNQYDVTI-------------------------------------
----TPEVASR-----TTNRVIMAELVRLY---KESDLGMRLPAYDGRKSLYTAGE-LPF
AWKEFTIKHVD--------------------------EEDGINGPKR----VREYKVVIK
FV-ARANMHHLGQFLAGKRAD---APQEALQILDIVLRELSTK--RYCPVGRSFFSPDIR
-----VPQRLGDGLESWC-------------------------G-------FYQS-----
-----------IRPTQMGLSLNIDM-ASAAFIEPLPVIGFVAQLVGK-----DALLRPLS
DSDRIKIKKALRGVKVEVTHRGNVRRKYRVSGLTSQPTRELV-----------------F
PVDDNS----------TMKSVVEYFQEMYGFTIQHT-HLPC--LQVGNQ-KKANYLPMEA
CKIVEGQRYTKRLNERQITALLKVTCQR-PRDRENDILQ------------------TVQ
HNAYDQDPYAK-EFGIKISEKLASVEARILPAPWLKYHE-TGKE--KDCLPQ---VGQWN
MMNKKMINGMTVSRW--ACINFSRSVQ---ESVARGFCNELAQMCQVSGMEFNPEPVIPI
--------YNARPDQ--------VEKALKHVYHASMNKTK---GKELELLLAILPDNNGS
L-YGDLKRICETDLGLISQCC-LTKHVFKT---SKHK----QYLANVSLKINVK------
----MGGRNTVLLDAISCRIP--LVSD---IPTIIFGADVTHP----ENGE-DSSPSIAA
VVASQDWPEVTKYAGLVCAQAHRQELIQDLYKTWQDPVRGTVSGGMIRDLLVSFRKATGQ
K-PLRIIFYR---DGVSEGQFYQVLLYELDAIRKACASL--EPNYQPPVTFIVVQKRHHT
RLFANNHRDRSSTDK--SGNILPGTVVDSKICHPTEFDFFLCSHA---------GIQGTS
RPAHYHVLWDENNFTADGIQSLTNNLCYTYARCTRSVSVVPPAYYAHLAAFRARFYMEPE
-MQENGS------------T-GGGSVHGTKGTR-AGESGVRPLPALKENV----------
------------------------KR----------------------------------

-------------VMF-------------------------------YC-----------

>Ricinus-communis_29844.t000058_RICCO

---------MPIRQMKESSEQHLVLKTHLQNTMNQPQKHHKIAQNGKGPPQSQ-------
---ETHNSKPQNQTSPPTKN
RGRRRGRGG--------------RKSDQGDVFTRPSSRPCTVVHKPVNQAGGL-------

---------LANAP----------NGNSGNICEMEMGL----------------------
-------------------------GFPT----SSKSLTYARRPGYGQL-GTK-CI-VKA
NHFFAEL--LD--KDLNQYDVTI-------------------------------------
----TPEVASR-----TTNRAIMAELVRLY---KESDLGMRLPAYDGRKSLYTSGE-LPF
AWKEFIIKLVD--------------------------EDDGVNGPKR----EREYKVVIK
FV-ARANMHHLGQFLAGKRAD---APQEALQILDIVLRELSTR--RYCPVGRSFFSPDIR

-----APQRLGDGLESWC-------------------------G-------FYQS-----

-----------IRPTQMGLSLNIDM-ASAAFIEPLPVIELVAQLLGK-----DVLSRPLS

DADRIKIKKALRGVKVEVTHRGNVRRKYRVSGLTSQPTRELV-----------------F

PVDDNS----------TMKSVVEYFQEMYGFTIQHT-HLPC--LQVGNQ-KKANYLPMEA

CKIVEGQRYTKRLNERQITALLKVTCQR-PRDRENDILQ------------------TVQ

HNAYDQDPYAK-EFGIKISEKLASVEARILPAPWLKYHD-TGKE--KDCLPQ---VGQWN

MMNKKMINGMTVSRW--ACINFSRSVQ---ESVARGFCSELAQMCQVSGMEFNPEPVIPI

--------YSARPEQ--------VEKALKHVYHASMNKTK---GKELELLLAILPDNNGT

L-YGDLKRICETDLGLISQCC-LTKHVFKI---S--K----QYLANVSLKINVK------

----MGGRNTVLLDAISCRIP--LVSD---IPTIIFGADVTHP----ENGE-DSSPSIAA

VVASQDWPEVTKYAGLVCAQAHRQELIQDLYKTWQDPVRGTVSGGMIRDLLVSFRKATGQ

K-PLRIIFYR---DGVSEGQFYQVLLYELDAIRKACASL--EPNYQPPVTFIVVQKRHHT

RLFANNHRDRSSTDK--SGNILPGTVVDSKICHPTEFDFYLCSHA---------GIQGTS

RPAHYHVLWDENNFTADGIQSLTNNLCYTYARCTRSVSVVPPAYYAHLAAFRARFYMEPE

-------------------M-QDNGSTGTRGTRAAGETGVRPLPALKENV----------

------------------------KR----------------------------------

-------------VMF-------------------------------YC-----------

>Populus-trichocarpa__POPTR_0008s15860_POPTR

---------MPVRQMKESSEQHLVIKTHMQNSMNQPQKHHKTAQNGKGPPQPLQ------

---ESSNTKPQNQASPPAKN

RGRRRGRGG--------------RKSDQGDVCTRPSSRPCTVAHKPV--LN---------

---------PTGDL----------LANASNGHIENSKN----------------------

-----VCEMEMGLG------------FPT----SSKSLSLAPRPGYGQV-GTK-CI-VKA

NHFLAEL--PD--KDLNQYDVTI-------------------------------------

----TPEVASR-----TMNRDIMAELVRLY---KDSDLGMRLPAYDGRKSLYTAGE-LPF

AWKEFIIKLID--------------------------EEDGINGPKR----GREYKVVIK

FV-ARANMYHLGQFLAGKRAD---APQEALQILDIVLRELSSK--RYCPVGRSFFSPDIR

-----APQRLGDGLESWC-------------------------G-------FYQS-----

-----------IRPTQMGLSLNIDM-ASAAFIEPLPVIEFVAQLLGK-----DILSRPLS

DSDRVKIKKGLRGVKVEVTHRGSVRRKYRVSGLTSQPTRELV-----------------F

PVDDNS----------TMKSVVEYFQEMYGFTIQHT-HLPC--LQVGNQ-KKANYLPMEA

CKIVEGQRYTKRLNERQITALLRVTCQR-PRDRENDILQ------------------TVQ

HNAYDQDPYAK-EFGIKISEKLASVEARILPAPWLKYHE-TGKE--KDCLPQ---VGQWN

MMNKKMINGMTVSRW--ACINFSRSVQ---ESVARGFCNELAQMCQVSGMEFNSEPVIPI

--------YNARPEH--------VEKALKHVYHASTNRTK---GKELELLLAILPDNNGS

L-YGDLKRICETDLGLITQCC-LSKHVFKI---S--K----QYLANLSLKINVK------
----MGGRNTVLLDAISCRIP--LVSD---IPTIIFGADVTHP----ENGE-DSSPSIAA
VVASQDWPEVTKYAGLVCAQAHRQELIQDLYKTWQDPVRGTVSGGMIRDLLISFRKATGQ
K-PLRIIFYR---DGVSEGQFYQVLLYELDAIRKACASL--EPNYQPPVTFIVVQKRHHT
RLFANNHRDRNSTDK--SGNILPGTVVDSKICHPTEFDFYLCSHA---------GIQGTS
RPAHYHVLWDENNFTADGIQSLTNNLCYTYARCTRSVSVVPPAYYAHLAAFRARFYTEPV
-MQENGS------------A-GSGACHGAKGTR-TGESGVRPLPALKENV----------
------------------------KR----------------------------------

-------------VMF-------------------------------YC-----------

>Populus-trichocarpa__POPTR_0010s09150_POPTR

-----------------MNRAIMAELVRLY---KESDLGMRLPAYDGRKSLYTAGK-LPF
AWKEFAIKLVD--------------------------AQDGISGPKR----EREYKVVIK
FV-ARANMYHLSQFLAGKHAD---APQEALQILDIVLRELSTK--RYCPVGRSFFSPDIR
-----APQRLGDGLESWC-------------------------G-------FYQS-----
-----------IRPTQMGLSLNIDM-ASAAFIEPLPVIEFVAQLLGK-----DVLSRPLS
DSDRVKIKKGLRGVKVEVTHRGSVRRKYRVSGLTSQPTRELV-----------------F
PVDDNS----------TMKSVVEYFQEMYGFTIQHA-HLPC--LQVGNQ-KKANYLPMEA
CKIVEGQRYTKRLNERQITALLKVTCQR-PRDRENDILQ------------------TVQ
NNAYDQDPYAK-EFGIKISEKLASVEARILPAPWLKYHE-TGKE--KDCLPQ---VGQWN
MMNKKMINGMTVSRW--ACINFSRSVQ---ESVARGFCNELAQMCQVSGMEFNSEPVIPI
--------YNARPEH--------VEKALKHVYHASTNRTK---GKELELLLAILPDNNGS
L-YGDLKRICETDLGLLTQCC-LSKHVFKI---S--K----QYLANVSLKINVK------
----MGGRNTVLLDAISCRIP--LVSD---IPTIIFGADVTHP----ENGE-DSSPSIAA
VVASQDWPEVTKYAGLVCAQAHRQELIQDLYKTWQDPVRGTVSGGMIRDLLISFRKATGQ
K-PLRIIFYRRVLDGVSEGQFYQVLLYELDAIRKACASL--EPNYQPPVTFIVVQKRHHT
RLFANNHRDRTSTDK--SGNILPGTVVDSKICHPTEFDFYLCSHA---------GIQGTS
RPAHYHVLWDENNFTADGIQSLTNNLCYTYARCTRSVSVVPPAYYAHLAAFRARFYTEPV
-MHETGS------------A-GSGAGHGAKGTR-TGESGVRPLPALKENV----------
------------------------KR----------------------------------

-------------VMF-------------------------------YC-----------

>Arabidopsis-thaliana_AT1G48410.2_ARATH
---MVRKRRTDAPSEGGEGSGS
REAGPVSGGGRGSQRGGFQQGGGQHQGGRGYTPQPQQGGRGGRGYGQPPQQQQQYG----
---GPQEYQGRGRGGPPHQG
GRGGYGGGRGG------------GPSSGPPQRQSVPELHQATSPTYQAVSS---------

---------QPTLS----------EVSPTQVPEPTVLA----------------------
-----QQFEQLSVEQGAPSQAIQP--IPS----SSKAFKFPMRPGKGQS-GKR-CI-VKA
NHFFAEL--PD--KDLHHYDVTI-------------------------------------
----TPEVTSR-----GVNRAVMKQLVDNY---RDSHLGSRLPAYDGRKSLYTAGP-LPF
NSKEFRINLLD--------------------------EEVGAGGQRR----EREFKVVIK
LV-ARADLHHLGMFLEGKQSD---APQEALQVLDIVLRELPTSRIRYIPVGRSFYSPDIG
-----KKQSLGDGLESWR-------------------------G-------FYQS-----
-----------IRPTQMGLSLNIDM-SSTAFIEANPVIQFVCDLLNR-----DISSRPLS
DADRVKIKKALRGVKVEVTHRGNMRRKYRISGLTAVATRELT-----------------F
PVDERN----------TQKSVVEYFHETYGFRIQHT-QLPC--LQVGNS-NRPNYLPMEV
CKIVEGQRYSKRLNERQITALLKVTCQR-PIDREKDILQ------------------TVQ
LNDYAKDNYAQ-EFGIKISTSLASVEARILPPPWLKYHE-SGRE--GTCLPQ---VGQWN
MMNKKMINGGTVNNW--ICINFSRQVQ---DNLARTFCQELAQMCYVSGMAFNPEPVLPP
--------VSARPEQ--------VEKVLKTRYHDATSKLS-Q-GKEIDLLIVILPDNNGS
L-YGDLKRICETELGIVSQCC-LTKHVFKM---S--K----QYMANVALKINVK------
----VGGRNTVLVDALSRRIP--LVSD---RPTIIFGADVTHP----HPGE-DSSPSIAA
VVASQDWPEITKYAGLVCAQAHRQELIQDLFKEWKDPQKGVVTGGMIKELLIAFRRSTGH
K-PLRIIFYR---DGVSEGQFYQVLLYELDAIRKACASL--EAGYQPPVTFVVVQKRHHT
RLFAQNHNDRHSVDR--SGNILPGTVVDSKICHPTEFDFYLCSHA---------GIQGTS
RPAHYHVLWDENNFTADGLQSLTNNLCYTYARCTRSVSIVPPAYYAHLAAFRARFYMEPE
-TSDSGSMASGSMAR----G-GGMAGRSTRGP--NVNAAVRPLPALKENV----------
------------------------KR----------------------------------

-------------VMF-------------------------------YC-----------

>Ricinus-communis_29677.t000007_RICCO
--MVRKRRTEAPASGGESSEPH
EAASGGSQRPYERNAPPQQGPGGPYQGGRSWGPQSQQGGRGGGGRGRSGGMSQQQQYG--
---GGPEYQGRGRGPPQQGG
RGGYGGGRSSSNRG---------GPPSVGPSRPPVPELHQATLAPYQAGVS---------

---------PQLMP----------SEGSSSSGPPEPSP----------------------
-VVVAQQMQELSIQQEVSSSQPIQAPPP-----SSKSMRFPLRPGKGST-GIR-CI-VKA
NHFFAEL--PD--KDLHQYDVTI-------------------------------------
----TPEVTSR-----GVNRAVMEQLVKLY---RESHLGKRLPAYDGRKSLYTAGP-LPF
ISKEFKITLID--------------------------EDDGSGGQRR----EREFRVVIK
LA-ARADLHHLGLFLQGRQAD---APQEALQVLDIVLRELPTT--RYCPVGRSFYSPDLG
-----RRQPLGEGLESWR-------------------------G-------FYQS-----
-----------IRPTQMGLSLNIDM-SSTAFIEPLPVIDFVNQLLNR-----DVSSRPLS
DADRVKIKKALRGVKVEVTHRGNMRRKYRISGLTSQATRELT-----------------F
PVDERG----------TMKSVVEYFYETYGFVIQHT-QWPC--LQVGNQ-QRPNYLPMEV
CKVVEGQRYSKRLNERQITALLKVTCQR-PQERERDIMQ------------------TVH
HNAYGNDPYAK-EFGIKISEKLASVEARILPAPWLKYHD-TGRE--KDCLPQ---VGQWN
MMNKKMVNGGTVNNW--ICINFSRNVQ---DSVARGFCYELAQMCYISGMAFNPEPVLPP
--------VSARPEQ--------VEKVLKTRYHDAMTKLQ-Q-GKELDLLIVILPDNNGS
L-YGELKRICETDLGLVSQCC-LTKHVFRM---N--K----QYLANVALKINVK------
----VGGRNTVLVDALSRRIP--LVSD---RPTIIFGADVTHP----HPGE-DSSPSIAA
VVASQDWPEVTKYAGLVCAQAHRQELIQDLFKEWQDPVRGRVTGGMIKELLISFRRATGQ
K-PQRIIFYR---DGVSEGQFYQVLLYELDAIRKACASL--EPNYQPPVTFVVVQKRHHT
RLFANNHNDRNAVDK--SGNILPGTVVDSKICHPTEFDFYLCSHA---------GIQGTS
RPAHYHVLWDENKFTADGLQSLTNNLCYTYARCTRSVSIVPPAYYAHLAAFRARFYMEPE
-TSDSGSMTSGPVGGRGGMG-GGAGARSTRGP--AASAAVRPLPALKENV----------
------------------------KR----------------------------------

-------------VMF-------------------------------YC-----------

>HbAGO1

-----------------------MVKQEGSSSQAIQ------------------------

---------ALPPP--
---------------------------------SSKSMRFPLRPGKGST-GIR-CI-VKA

NHFFAEL--PD--KDLHQYDVTI-------------------------------------
----TPEVSSR-----GVNRAVMEQLVKLY---KESHLGKRLPAYDGRKSLYTAGP-LPF
ISKEFKIILID--------------------------EDDGSGGPRR----EREFRVVIK
LA-ARADLHHLGLFLQGRQAD---APQEALQVLDIVLRELPTT--RYCPVGRSFYSPDLG
-----RRQPLGEGLESWR-------------------------G-------FYQS-----
-----------IRPTQMGLSLNIDM-SSTAFIEPLPVIDFVTQLLNR-----DVSSRPLS
DADRVKIKKALRGVKVEVTHRGNMRRKYRISGLTSQATRELT-----------------F
PVDERG----------TLKSVVEYFYETYGFVIQHT-QWPC--LQVGNQ-QRPNYLPMEV
CKIVEGQRYSKRLNERQITALLKVTCQR-PHERELDIMQ------------------TVH
HNAYHNDPYAK-EFGIKISEKLASVEARILPPPWLKYHE-TGRE--RDCLPQ---VGQWN
MMNKKMVNGGTVNNW--ICINFSRNVQ---DSVARGFCHELAQMCYISGMAFNPEPVLPP
--------ISGRPEQ--------VEKVLKTRYHDAMTKLQPQ-GRELDLLIVILPDNNGS
L-YGDLKRICETDLGLVSQCC-LTKHVFKM---S--K----QYLANVALKINVK------
----VGGRNTVLVDALSRRIP--LVSD---RPTIIFGADVTHP----HPGE-DSSPSIAA
VVASQDWPEITKYAGLVCAQAHRQELIQDLFKEWQDPVRGKVSGGMIKELLISFRRATGQ
K-PQRIIFYR---DGVSEGQFYQVLLYELDAIRKACNSL--EPNYQPPVTFVVVQKRHHT
RLFANDHSDRNAVDR--SGNILPGTVVDSKICHPTEFDFYLCSHA---------GIQGTS
RPAHYHVLWDENKFSADGLQSLTNNLCYTYARCTRSVSIVPPAYYAHLAAFRARFYMEPE
-TSDSGSMTSGPVSGR---G-AMGGGTGARSTRGAASAAVRPLPALKENV----------
------------------------KR----------------------------------

-------------VMF-------------------------------YC-----------

>Populus-trichocarpa__POPTR_0012s03410_POPTR
--------------------------------------MVRKRRTEIPQSGGESSESQET
DTGRGAQPPAERSGPPQQGGGGGGYQGGRGWGPQSQQGGRGGGYGGRGRGGMQQQQYG--
---GAPEYQGRGRGQPQQGG
RGYGGGRPGGGR-----------GGPSSGGFRPPAPELHQATPAPYPAVVT---------

---------TQPTP----------SEASSSMRPPEPSL----------------------
-ATVSQQLQQLSVEQEGSSSQAIQP-LPA----SSKSVRFPLRPGKGST-GIR-CI-VKA
NHFFAEL--PD--KDLHQYDVTI-------------------------------------
----TPEVTSR-----GVNRAVMEQLVKLY---RESHLGKRLPAYDGRKSLYTAGA-LPF
QAKDFKITLID--------------------------DDDGSGGPRR----EREFKVTIK
LA-ARADLHHLGLFLRGQQAD---APQEALQVLDIVLRELPTA--RYCPVGRSFYSPDLG
-----RRQSLGEGLESWR-------------------------G-------FYQS-----
-----------IRPTQMGLSLNIDM-SSTAFIEPLPVIDFVTQLLNR-----DVSSRPLS
DSDRVKIKKALRGVKVEVTHRGNMRRKYRISGLTSQATRELT-----------------F
PVDERG----------TLKSVVEYFYETYGFVIQHP-QWPC--LQVGNQ-QRPNYLPMEV

CKIVEGQRYSKRLNERQITALLKVTCQR-PQEREKDIMQ------------------TVY
HNAYHNDPYAK-EFGIKISDKLASVEARILPPPWLKYHD-TGRE--KDCLPQ---VGQWN
MMNKKMVNGGRVNNW--ICVNFSRNVQ---DSVARGFCYELAQMCQISGMDFALEPLLAP
--------VSGRPEH--------VERVLKNRYHEAMTKLRPH-SKELDLLIVILPDNNGS
L-YGDLKRICETDLGLVSQCC-LTKHVFKM---S--K----QYLANVALKINVK------
----VGGRNTVLVDAISRRIP--LVSD---RPTIIFGADVTHP----HPGE-DSSPSIAA
VVASQDWPEVTKYAGLVCAQAHRQELIQDLYKTWQDPVRGTVSGGMIKELLISFRRATGQ
K-PQRIIFYR---DGVSEGQFYQVLLYELDAIRKACASL--EPNYQPPVTFVVVQKRHHT
RLFANDHRDRNAVDR--SGNILPGTVVDSKICHPTEFDFYLCSHA---------GIQGTS
RPAHYHVLWDENKFTADGLQSLTNNLCYTYARCTRSVSIVPPAYYAHLAAFRARFYMEPE
-TSDSESIASGMAGGRG--G-AGGGPRPTRGP--GANAAVRPLPALKENV----------
------------------------KR----------------------------------

-------------VMF-------------------------------YC-----------

>Populus-trichocarpa__POPTR_0015s05550_POPTR
--------------------------------------MVRKRRTELPRSGGESSESQET
GAGRGAQPPAERSGPPQQGGGGGGYQGGR--GPQSQQVGRGGGYGGGRGRGGMQQQHYG-
---GAPEYQGRGRGQPQHGE
RGYGSGRSGGGRG----------GPPSGGPFRAPAPELHQATPAPYPAGMT---------

---------PQPMPSEARSSMPMLSEASSSMQPLEPSP----------------------
-AAVSQQMQQLSIQQEGSSSQATQP-PPA----SSKSMRFPLRPGKGST-GIR-CI-VKA
NHFFAEL--PD--KDLHQYDVSI-------------------------------------
----TPEVSSR-----GVNRAVMAQLVKLY---QESHLGKRLPAYDGRKSLYTAGA-LPF
QAKEFKIILID--------------------------EDDGTGGQRR----EREFKVVIK
FA-ARADLHHLGLFLQGKQAD---APQEALQVLDIVLRELPTA--RYCPVGRSFYSPDLG
-----RRQSLGEGLESWR-------------------------G-------FYQS-----
-----------IRPTQMGLSLNIDM-SSTAFIEPLPVIDFVTQLLNR-----DVSSRPLS
DSDRIKIKKALRGVRVEVTHRGNMRRKYRISGLTSQATRELT-----------------F
PVDERG----------TLKSVVEYFYETYGFVIQHT-QWPC--LQVGNQ-QRPNYLPMEV
CKIVEGQRYSKRLNERQITALLKVTCQR-PQERERDIMQ------------------TVY
HNAYHNDPYAK-EFGIRISEKLASVEARILPPPWLKYHD-TGRE--KDCLPQ---VGQWN
MMNKKMVNGGRVNNW--ICINFSRTVQ---DSVARGFCYELAQMCHISGMDFALEPLLPP
--------VGARPEQ--------VERVLKTRYHDAMTKLQPH-SKELDLLIVILPDNNGS
L-YGDLKRICETDLGLVSQCC-LTKHVFKM---S--K----QYLANVALKINVK------
----VGGRNTVLVDALSRRIP--LVSD---RPTIIFGADVTHP----HPGE-DSSPSIAA
VVASQDWPEVTKYAGLVCAQAHRQELIQDLYKTWQDPVRGTVSGGMIKELLISFRRATGQ
K-PQRIIFYR---DGVSEGQFYQVLLHELDAIRKACASL--EPNYQPPVTFVVVQKRHHT

RLFANDHRDRNAVDR--SGNILPGTVVDSKICHPTEFDFYLCSHA---------GIQGTS
RPAHYHVLWDENKFTADGLQSLTNNLCYTYARCTRSVSIVPPAYYAHLAAFRARFYMEPE
-TSDSGSLTSGMASGRG--G-GGAGGRATRGP--AANAAVRPLPALKENV----------
------------------------KR----------------------------------

-------------VMF-------------------------------YC-----------

>Populus-trichocarpa__POPTR_0006s12010_POPTR

----------------------MYGRGRRGGSPAPTKGGGRGRGRGAPLPSP--------

-----------------------MASSEADSISSVSQLGGEMERLSV--QT---------

---------EPPAP----------TQAPAAIPAPQQQK----------------------
-----QQQQQLV---------------------PASSVKFAQRPDHGTV-GSR-CL-IRA
NHFLVEL--AD--RDLHHYDVSI-------------------------------------
----TPEVASR-----GVNRAIMRELLASN----STHFQSRKPAYDGRKGFYTAGP-LTF
TSKDFVVTL----------------------------VDKDDQGSVRK---ERKFKVTVR
LA-SKTDLYHLKEFLQGRQRG---APHDTIQVLDVVLREPPSNKQVCTIVGRSFFTAGLG
-----GQNEIGNGIECWK-------------------------G-------FYQS-----
-----------LRPTQMGMSLNIDV-SVAAFYEPILAVDFVAKLLNLGDPI-RAATRPLS
DSDRAKLKKALRGVRVKVTHGEEKR--YKITGISASATNQLR-----------------F
AAEDG-----------KQKSVVQYFLEKYNIRLRFA-SWPA--LQSGND-SRPIFLPMEC
CKIIEGQRYSKKLNEKQVTALLREACRR-PVEREHSIEQ------------------IVH
FNDVAQDDLAK-EFGVSVKKELTCIDARVLPPPVLKYHD-LGKA--RTVRPR---VGQWN
MINAKLFNGATVNFW--MCVNFSSLGE----QMAASFCRALVGMCNNKGMVINPAPVFPI
--------RSGHPNQ--------LEKTLAEVHSMCNNER-----KQLQILIIILPDVSGS
--YGTIKRVCETELGIVSQCC-QPKQARKC---S--P----QYLENVALKINVK------
----AGGRNTVLEDALNRRIP--LLSD---TPTIIFGADVTHP----QPGE-DSSPSIAA
IVASMDWPEVTTYRGLVSAQKHRQEIIQDC-------------AGMIRELMIAFRRTTNQ
K-PSRIIFYR---DGVSEGQFSQVLLYEMDAIRKACASL--EPNYLPPVTFIVVQKRHHT
RLFATN---PNQTDK--SGNILPGTVVDTKICHPSEHDFYLCSHA---------GIQGTS
RPVHYHVLCDMNKFTADCLQMLTNNLCYTYARCTRSVSVVPPAYYAHLAAFRARYYIEGD
-------------------IASDSGGGGTGPPVRREAAPVRPLPAISPNV----------
------------------------KN----------------------------------

-------------VMF-------------------------------YC-----------

>Arabidopsis-thaliana_AT2G27880.1_ARATH
--MSNR
GGGGHGGASRGRGGGRRSDQRQDQSSGQVAWPGLQQSYGGRGGSVSAGRGRGNV------
---GRGENTGDLTATQVPVA
SAVSGGRG---------------RGNIGDPTFSVASSSKTVSVASSSKEES---------

---------KNTEV----------SETMSNLQITSTET----------------------
-----KPEMTSL--------------PPA----SSKAVTFPVRPGRGTL-GKK-VM-VRA
NHFLVQV--AD--RDLYHYDVSI-------------------------------------
----NPEVISK-----TVNRNVMKLLVKNY---KDSHLGGKSPAYDGRKSLYTAGP-LPF
DSKEFVVNLAEK-------------------------RADGSSGK------DRPFKVAVK
NV-TSTDLYQLQQFLDRKQRE---APYDTIQVLDVVLRDKPSN--DYVSVGRSFFHTSLG
KDARDGRGELGDGIEYWR-------------------------G-------YFQS-----
-----------LRLTQMGLSLNIDV-SARSFYEPIVVTDFISKFLNI-----RDLNRPLR
DSDRLKVKKVLRTLKVKLLHWNGTKS-AKISGISSLPIRELR-----------------F
TLEDK-----------SEKTVVQYFAEKYNYRVKYQ-ALPA--IQTGSD-TRPVYLPMEL
CQIDEGQRYTKRLNEKQVTALLKATCQR-PPDRENSIKN------------------LVV
KNNYNDD--LSKEFGMSVTTQLASIEARVLPPPMLKYHD-SGKE--KMVNPR---LGQWN
MIDKKMVNGAKVTSW--TCVSFSTRID---RGLPQEFCKQLIGMCVSKGMEFKPQPAIPF
--------ISCPPEH--------IEEALLDIHKRAP---------GLQLLIVILPDVTGS
--YGKIKRICETELGIVSQCC-QPRQVNKL---N--K----QYMENVALKINVK------
----TGGRNTVLNDAIRRNIP--LITD---RPTIIMGADVTHP----QPGE-DSSPSIAA
VVASMDWPEINKYRGLVSAQAHREEIIQDLYKLVQDPQRGLVHSGLIREHFIAFRRATGQ
I-PQRIIFYR---DGVSEGQFSQVLLHEMTAIRKACNSL--QENYVPRVTFVIVQKRHHT
RLFPEQHGNRDMTDK--SGNIQPGTVVDTKICHPNEFDFYLNSHA---------GIQGTS
RPAHYHVLLDENGFTADQLQMLTNNLCYTYARCTKSVSIVPPAYYAHLAAFRARYYMESE
-------------------M-SDGGSSRSRSSTTGVGQVISQLPAIKDNV----------
------------------------KE----------------------------------
-------------VMF-------------------------------YC-----------

>HbAGO5.1
--MADRDT
KHFLPGGDPNNIMVGANGAFHPRDCVVSDAPVPMNYVRDSWISQREKFLNMLPQNPSYA-
--VLPETSGAHSMQVLQPPNSS

RDEKVGRI---------------EEPSVNKEGSQLKKRQGGGAPKTP--KA---------

---------KKPRK----------PKDNSNNAVQRVKP----------------------
-----AKKSMDVVINGIDMDISGIP-IPVCSCTGTPQQCY--RWGCGGWQSAC-CT----
-------------TNVSMYPLPM-------------------------------------
----STKRRGARIAGRKMSQGAFKKVLEKL--------------AAEGYNFANPID-LRT
HWAKHATRL----------------------------LHSATESSNERFHAHRGSTNPCK
DS-LDIPIIDIAIIMIG-------CLNDGCFEIRVPVTRKARE------AGEEYASLVFE
-----GTGSPPPELHFLQCFPPRADFRSCFFNHFASIFSLPHVG-------FFEGLISWA
HLCVYKKVRLYLHQTSMGLLLTRPILSATSYFDPIMVTDFVAKYFRL-----RDMSSPLS
EQDCIKLKKALKGVRVELSHREYAES-HKITGVSDQPLNQIF-----------------F
TFDDKS----------TNVSLVQYFRERYNIGLKYT-SLPA--LQVGSC-SKPIYLPMEV
CRIVEGQRYSKKLNERQVIALLKATCQR-PHERENSIKH------------------IIW
QNDYNRDELMRNEHGIQVKEELTFIDARVLPPPM------TGVE--ACADPH---LGQWN
MINKKMVNGGRVEFW--TCVNFSLQVN---QNLPVEFCRQLIDMCVSKGMGFNPNPILPV
--------QSAHPSQ--------IERALADVHKKCTAKLSNE-KKHLQLLIIILPDVNGS
--YGKIKRVCETELGIVSQCC-QPRQAAML---S--K----QYFENVALKINVK------
----VGGRNTVLNDAIQRRIP--LVTD---VPTIIFGACVTHP----HPGQ-DTCPSIAS
VVASMDWPEVTKYRGMVSAQGHREEIIQDLYKSYHESDRGLVHSGVIRELCIAFKRVTGH
K-PSRLILYR---FGVSEGQFFQALLPEIDAIRMACSSL--EEGYLPPVTYIVVQKRHHT
RLFPVD---RRQTDR--SGNILPGTVIDTRICHPMEFDFFLKSHA---------GIQGTS
IPTRYHVLYDENHFAADGLQVLTNDLCYTYARCTRSVSIVPPVSYAHLAASRARYYIKCE
-TSD---------------G-GSSGGRSTTG---SSREEVQPLPLIKDNV----------
------------------------KD----------------------------------

-------------VMF-------------------------------YC-----------

>HbAGO5.2

----------------------M-------------------------------------
----TPEVASK-----KVK---

---RTLFSPNFG

-----PRDGLGDDIEYWR-------------------------G-------YYQS-----
-----------LRPTQMGLSFNIDV-SATSFFEPIMVTEFVAKYFSL-----KDMSRPLS
EQEHIKIKEGLKGLRVELSHRENAEC-HKITSVSDQPLNQISNFLKPCLNHRHSKIYRLF
NLDDNN----------TNMSVVQYFRERYNVELSYT-SLPALQLQARSD-SNPIYLPMEL
CRILEGQRYSRNLNLRQVIALLKATCQR-PHERENSIKR------------------IIR
QNNYSGDELVRNEFGIQ-----------------LKYHE-TGDE--ASVVPR---LGQWN
MINKKVVNGGRVEFW--TCVNFSLRVN---QNVPVEFCRQLIEMCVSKGMEFNPNPILPI
--------QSANPSQ--------IGKALADVHKQCTAKLSNG-KKRLQLLIIILPDVSGS
--YGEIKRVCETELGIVSQCC-LPRKAARL---S--K----QYFENVALKINVK------
-----------------RRIP--LVTD---VLTIIFGADITHP----PSRV-GTDPSIAA
VVASMDWPEITKYRGIVSGQAYHEEIIQDLYKSYHDPDRGLIHSGMIRELFIAFRRATGH
K-PSRIIFYR---DSVSEGKFSEVLLHEMNAIRKACSSL--EEEYLPPVTYVVVRKRHHT
RLFPVD---RGETDR--SGNILPGTVIDTKICLQKEFDFYLNSHS---------AIQGTS
RPTHYHVLYDENHFIADGLQVLTNNLCYMNARCTRSVSIVPPVYYAHLAALRARYYIDGE
---------------------TLHGGSSGGRSTTGRSGEVQPLPMIKDNV----------
------------------------KD-----------------------------------
----
-------------VIWPSLPCTAPLSSDIIRATPALTRGGGGGRGGRFCRGCVLTSTFTQ
DAPPLSSASPPISRSPAVEELRRELEQKRTTGDQVSKAGTSPALSKAIRFVPRPGFGTAG
RKCVVKANHFLVEIADRDLCRYDVTITPERISTKVNRDIISELVLKYSESHLGNRMPAYD
GRKSLYTAGPLPFEYEEFVVKLVEKNNDAEISTKVNRDIISELVLKYCESHLGNRMPAYD
GRKSLYTAGPLAFEYEEFVVKLVEKNNDAGSFGSTMRERQFNVSIKFSSKVDVHHLRQFL
SGRQMDAPQETLQALDIVLRASPSKKYYSFAVLWSILNCLLFSHYLTFHLSFCIQIFLLN
LIAEVYEC
>HbAGO5.3
---MASGEGSRKR
KKEREISRKVKEMKHSELLQTPGHEPGEIQGGPPRPSGPPIAEAVRSPPRREEPPPPPPV
--APSTEEGRSQPPARSPS
RGAQRGGGGRRGGRD--------GSGRAGRGYNPAPTFTQAAPPQASAFPPISRSAASAF

---------HPRIM----------SVAPSQMRPQALAS-------------SSRASPSQA
AASSSAAVEELRLEMEQKLTTGDQVTKAETPPATSKAVRFTPRPGFGSA-GKK-CV-IKA
NHFLVEI--AD--RDLCRYDVTM--------------------------------------
----TPEVASK-----KVKRYIISQLVSMY---RESHLGNRMPAYDGRKGLYTAGP-LPF
ESKEFVIKLVD--------------------------ENNG-AGSSASTTKERQFKVAIK
FA-SKVDIHHLRRFLSCRQMD---APQEMIQVLNIVLRASIPE--NYSSVGRTLFSPNFG
-----PRDGLGDDIEYWR-------------------------G-------YYQS-----
-----------LRPTQMGLSFNIDV-SATSFFEPIMVTEFVAKYFSL-----KDMSRPLS
EQEHIKIKEGLKGLRVELSHRENAEC-HKITSVSDQPLNQIS-------------------F
NLDDNN----------TNMSVVQYFRERYNVELSYT-SLPALQLQARSD-SNPIYLPMEL
CRILEGQRYSRNLNLRQVIALLKATCQR-PHERENSIKR------------------IIR
QNNYSGDELVRNEFGIQVKEELTFINARVLPPPMLKYHE-TGDE--ASVVPR---LGQWN
MINKKVVNGGRVEFW--TCVNFSLRVN---QNVPVEFCRQLIEMCVSKGMEFNPNPILPI
--------QSANPSQ--------IGKALADVHKQCTAKLSNG-KKRLQLLIIILPDVSGS

--YGEIKRVCETELGIVSQCC-LPRKAARL---S--K----QYFENVALKINVK------
----AGGRNTVLNDAIQRRIP--LVTD---VLTIIFGADITHP----PSRV-GTDPSIAA
VVASMDWPEITKYRGIVSGQAYHEEIIQDLYKSYHDPDRGLIHSGMIRELFIAFRRATGH
K-PSRIIFYR---DSVSEGKFSEVLLHEMNAIRKACSSL--EEEYLPPVTYVVVRKRHHT
RLFPVD---RGETDR--SGNILPGTVIDTKICLQKEFDFYLNSHS---------AIQGTS
RPTHYHVLYDENHFIADGLQVLTNNLCYMNARCTRSVSIVPPVYYAHLAALRARYYIDGE
---------------------TLHGGSSGGRSTTGRSGEVQPLPMIKDNV----------
------------------------KD----------------------------------

-------------VMF-------------------------------YC-----------

>Populus-trichocarpa__POPTR_0001s22120_POPTR

-----------------------MASS----------------------SN---------

---------APPPS----------ASSSASMEELSQEI----------------------
-----AKKLSFGSTSATG-----GS-VPV----SSKAIVPPPRPQLGRI-GRK-CT-IRA
NHFVVEV--SD--RDLFHYDVAI-------------------------------------
----TPEITSK-----KVNRDVISQLVRSY---RESHLGNRMPAYDGRKSLYTAGA-LPF
EAKEFVVKLVE--------------------------KNDPASSSSS----ERQFNVAIK
YA-SKVDMNHLKEFLSGRQKD---VPQETIQILDIVLRASPSE--KYVTVGRSFFSLDLG
-----KKGELGNGIEYWR-------------------------G-------YYQS-----
-----------LRPTQMGLSLNIDV-SARSFYEPILVTEFVAKYFNL-----RDLSRPLS
DQDRVKVKRALRGIKVEISYRDYARS-FKVTGISNLPVDKTM-----------------F
TLDDKK----------TKVSVHQYFWDRYNIGLKYT-SLPP--LQAGTD-AKPIYLPMEL
CKIAGGQRYTKKLNERQVTALLRATCQR-PSARENSIKE------------------ANN
LSSTSLNVLVRNEFGIQVKEELTSVDARVLPPPMLKYHD-TGRE--ARVDPH---LGQWN
MINKKMVNGGKIDFW--TCVNFSTRVQ---RDLPFEFCWQLMDMCNSKGMEFHPDPIIQI
--------HSADSRH--------IEKALHDVHKKCTAKLANQKGKQLQLLIIILPDFSGS
--YGKIKRICETELGIVSQCC-QPQQAKKL---S--K----QYLENVALKINVK------
----AGGRNTVLNDAIQRRIP--NVTD---LPTIIFGADVTHP----QPGE-DSSPSIAA
VVASMDWPEVTKYRGLVSAQAHREEIIQDLYKKYQDPQKGLVHSGMIRELFIAFRRSTGQ
K-PHRIIFYR---DGVSEGQFSQVLLHEMQAIREACGTL--EEGYCPPVTFVVVQKRHHT
RFFPADHSKRDLTDR--SGNILPGTVVDTKICHPTEFDFYLNSHA---------GIQGTS
RPTHYHVLFDENNFTADGLQTLTNNLCYTYARCTRSVSIVPPAYYAHLAAFRARYYIEGE
-------------------T-SDSGSTGATG----RSVEARSLPVVKENV----------
------------------------KD----------------------------------

-------------VMF-------------------------------YC-----------

>Populus-trichocarpa__POPTR_0009s00660_POPTR

MSRRGGGRRSETRRDQDSS----------APSPSFQRGGGGGGRGRGGRGVSTP------
--APSPTPVR
APTSAAPGY--------------LSATAGP------------SAKPT--PQ---------

---------AAYTA----------AAASSSSSVGELSQ----------------------
-----ETAKKLTLG---------GL-VPV----SSKAIVPPRRPDYGKI-GKK-CV-IRA
NHFVVEV--SD--RDLFHYDVAI-------------------------------------
----TPEITSK-----KVNRDVISQLVRSY---RESHLGNRMPAYDGRKSLYTAGA-LPF
EAKEFVVKLAE--------------------------RGDP-ASSSSSVKKERQFKVAIK
YA-SKVDMYHLKEFLSGRQAD---APQETIQILDIVLRASPSE--KYITVGRSFFSPDLG
-----PKGDLGDGIEYWR-------------------------G-------YYQS-----
-----------LRPTQMGLSFNIDV-SARSFYEPILVTEFVAKYFNF-----RDLSRPLS
DQERVKVKRALRGIKVQITYSDYTKS-YKVTGISNLPVNKTM-----------------F
TLDDKK----------TKVSVYQYFLERYNIGLKYT-SLPP--LQAGTD-AKPIYLPMEL
CQIAGGQRYTKKLNERQVTALLRATCQR-PSARENNIKQANNLSLTSLFPSLRILIFMVR
QNDYSKNALVRDEFGIQVKEELTLVDARVLPPPMLKYHD-TGRE--ARVDPR---FGQWN
MIDKKMVNGGRIDFW--TCLNFSTRVH---RELPSEFCWQLMDMCNNKGMEFNPEPIIPI
--------RSADSRQ--------IEKALHDVHKQCTAELANQKGKQLQLLIIILPDVTGS
--YGKIKRVCETELGIVSQCC-QPQQAKKL---S--K----QYMENVALKINVK------
----AGGRNTVLNDAFHRRIP--LLTD---VPTIVFGADVTHP----QAGE-DAGPSIAA
VVASMDWPEVTKYRGLVSAQAHREEIIEDLYKKYQDPKKGLVHGGMIRELLIAFKRSTGQ
K-PFRIIFYR---DGVSEGQFSQVLLHEMQAIRQACGSL--EEGYCPRVTFVVVQKRHHT
RFFPADHSRRDQTDK--SGNILPGTVVDTTICHPTEFDFYLNSHA---------GIQGTS
RPTHYHVLFDENNFSSDGLQTLTNNLCYTYARCTRSVSIVPPAYYAHLAAFRARYYIEGE
---------------------TSDAGSS------GGTAEFRPLPVIKENV----------
------------------------KD----------------------------------

-------------VMF-------------------------------YC-----------

>Ricinus-communis_29589.t000074_RICCO

----------MSHRGGGRRQESQRDRQSSATSPSFNRGGGGGRGGRGRGGAG--------
--SFYAQPAPPPAG
SDFP-------------------SLSRPPTTSSRAAAAPQAAPPSSSSRAP---------

---------PAPAP----------APAPPPAAAASTST----------------------
------QIEKLTHGVEQLTTTAGAP-TPS----SSKEIRFPNRPGYGSI-GMK-CV-VKA
NHFLVDV--AD--RDLRQYDVSI-------------------------------------
----TPELTSK-----KINRDVISQLIRMF---RQSHLGNRRAAYDGRKSLYTAGP-LPF
ESKEFVVKLV---------------------------ESNKNAGSSVSSKKEREFKVAIK
FA-SKPDIHHLKQFLIGRQMD---CPQETIQVLDIVLRETPSE--KYTPVGRSFFSPDLG
-----QKGELGDGIEYWR-------------------------G-------YYQS-----
-----------LRPTQMGLSLNIDV-SARSFYEPIIVTDFVSKYLKL-----RDMSRPLS
DQDRIKVKKALKSVKVQILHREYAKS-YKVTGISNKPLNQIF-----------------F
KLDDKS----------TDISVVQYFREKYNIGLKYT-SLPA--LQAGSD-AKPIYLPMEL
CKIVDGQRYSKKLNERQVTALLRATCQR-PHEREESIKQ------------------MVK
RNSYNQDVLVRDEFGIQVKEELTFVDARVLPAPMLNYHE-TGRE--SRVDPR---CGQWN
MINKKMVNGGSVNFW--TCVNFSLNIN---RDLPAEFCRQLIQMCVSKGMAFNPNPIIPI
--------SSAHPGQ--------IGKTLNDIKRQCEAKLV----KQLQLLIIILPDISGS
--YGIIKRVCETELGIVSQCC-QPRQAAKL---S--K----QYFENVALKINVK------
----VGGRNTVLNDAVQRRIP--LVTD---CPTIIFGADVTHP----PPGE-DSSPSIAA
VVASMDWPEVTKYRGIVSAQAHREEIIQDLYKSFQDPQGILKHSGMIRELFVAFRRETGM
K-PKRIIFYR---DGVSEGQFSQVLLYEMDAIRKACASL--EEGYLPPVTFVVVQKRHHT
RLFPVD---RGQTDR--SGNILPGTVIDTKICHQREFDFYLNSHA---------GIQGTS
RPTHYHVLYDENHFTADNLQVLTNNLCYTFARCTRSVSIVPPAYYAHLAAFRARYYIEGE
---------------------MSDGGSTSGKSTTGRSKEVQPLPVIKDNV----------
------------------------KD----------------------------------

-------------VMF-------------------------------YC-----------

>HbAGO5.4

--MG
-----PRGELGDGIEYWR-------------------------G-------YYQS-----
-----------LRPTQMGLSFNVDV-SARSFFEPIMVTDFVAKYFRL-----RDLSRPLS
EQDRIKVKRSLKGIKVELRHREYPKS-YKITGVSNKPMSQTFIMPNFFISSS-------F
TPDDNS----------SDVSVVQYFRARYNIGLQYT-SLPA--LQAGSD-SKPIYLPMEL
CRIVEGQRYTKKLNDRQVTALLRATCQR-PHERENSIKQ------------------MVR
RNSYSRDELVSNEFGIQ-----------------LKYHE-TGGE--SRVDPQ---YGAWN
MINKKMVNGGMVDFW--TCVNFSMQVH---RNLPHDFCYQLIQMCVSKGMGFNPNPIIPV
--------QSAHPSQ--------IERALADVHKQCTAKLANE-KKRLQLLIIILPDLSGS
--YGKIKRICETEYGIVSQCC-QPKQAAKL---S--K----QYFENVALKINVK------
----VGGRNTVLNDAIQRRIP--LVTD---LPTIIFGADVTHP----APGE-DSVPSIAA
VVASMDWPEVTKYRGLVSAQAHREEIIQDLYKSYHDPDKGLVHSGMIRELLISFRRATGF
K-PGRIIFYR---DGVSEGQFSQVLLHEMDAIRKACSSL--EEGYLPRVTFVVVQKRHHT
RLFPVD---RGQADR--SGNVLPGTVIDTKICHPKEFDFYLNSHA---------GIQGTS
RPTHYHVLYDENRFTADGLQILTNNLCYTYARCTRSVSIVPPAYYAHLAAFRARYYIEGE
---------------------TSDGGSSSGRSTTARSREFQPLPVIKDNV----------
------------------------KDMLVKAFCMHIHYGFVLFSSGKLQRKSVSLRLGGL
EMLRIKTIYVAAMPWFQAKTGPGILAPSNERTPLVLSLVKPAGRLQTYPVAQNQRSHQLL
YQTLSSLQSLHNSLMY-------------------------------HCHPHIGTCSRTG
CFEKNRENPMIEFQQCIVGLNMRSMKLLMSFQDQTKEIHDTTSRLQYSTMPKAISLSELV
QLEPMTGRNPPLESKSSRKRRKNKCNVRKYGSDDNSDRKSHCHSCFSAFAVVSLRSQCCD
IPLGSYIQDHGIKGHLKSQGQTLCINYLISLLKETMCMTEERTTLVWPIQSLAMLFYQIC
PVGKCNCRAMTITKHILKINASG-------------------------------------

>HbAGO5.5

-------------MSRGGRQDSGRDQQSSSLSPSFQRGGGGGRGGRFCRGCV--------

----------------------LTSTFTQD------------------------------

---------APPLS----------SASPPISRSPAVEE----------------------
-----LRRELEQKRTTGDQVSKAGT-SPA----LSKAIRFVPRPGFGTA-GRK-CV-VKA
NHFLVEI--AD--RDLCRYDVTI-------------------------------------
----TPEVTSK-----KVNRDIISQLVRMY---RESHLGNRMPAYDGRKSLYTAGP-LPF
ESKEFVVKLVE--------------------------RNDG-AGSSGSTTRERQFKVAIK
FA-AKADLHHLQQFLYGRQMD---APQETIQVLDIVLRASPSE--KYITVGRSFFSSDLG
-----PRGELGDGIEYWR-------------------------G-------YYQS-----
-----------LRPTQMGLSFNVDV-SARSFFEPIMVTDFVAKYFRL-----RDLSRPLS
EQDRIKVKRSLKGIKVELRHREYPKS-YKITGVSNKPMSQTF-----------------F
TPDDNS----------SDVSVVQYFRARYNIGLQYT-SLPA--LQAGSD-SKPIYLPMEL

CRIVEGQRYTKKLNDRQVTALLRATCQR-PHERENSIKQ------------------MVR
RNSYSRDELVSNEFGIQVKEELTYVDARVLPPPMLKYHE-TGGE--SRVDPQ---YGAWN
MINKKMVNGGMVDFW--TCVNFSMQVH---RNLPHDFCYQLIQMCVSKGMGFNPNPIIPV
--------QSAHPSQ--------IERALADVHKQCTAKLANE-KKRLQLLIIILPDLSGS
--YGKIKRICETEYGIVSQCC-QPKQAAKL---S--K----QYFENVALKINVK------
----VGGRNTVLNDAIQRRIP--LVTD---LPTIIFGADVTHP----APGE-DSVPSIAA
VVASMDWPEVTKYRGLVSAQAHREEIIQDLYKSYHDPDKGLVHSGMIRELLISFRRATGF
K-PGRIIFYR---DGVSEGQFSQVLLHEMDAIRKACSSL--EEGYLPRVTFVVVQKRHHT
RLFPVD---RGQADR--SGNVLPGTVIDTKICHPKEFDFYLNSHA---------GIQGTS
RPTHYHVLYDENRFTADGLQILTNNLCYTYARCTRSVSIVPPAYYAHLAAFRARYYIEGE
---------------------TSDGGSSSGRSTTARSREFQPLPVIKDNV----------
------------------------KD----------------------------------

-------------VMF-------------------------------YC-----------

