# Supplementary material for: Post-transcriptional regulation of several biological processes involved in latex production in Hevea brasiliensis
Source: PeerJ. 2020 Apr 29;8:e8932. doi: 10.7717/peerj.8932 (PMC7195832; doi:10.7717/peerj.8932)
Supplement: Figure S2 [file peerj-08-8932-s024.pdf]

>CHLRE1

--MKVPGQAL
RPLPA---LRSYQAELLQ
LAGCHDAIVYLETGTGKTRIAVELIAAAL------------PALRAAGQS--AVFLAPSV
PLVRQQAEVLASTPVLVPEPEEPGAGEERERQEQQLAAGAAGSAPCQPPEEEAGMAEPEP
EAAAGAGAAGQQERTQRPPAGRRLRLRRL-----LRVEWLGGSC-------VHEGWGLAG
WRELLGRL--------------DVLVCTPAVLLGALVHAYVRVSDVGLLVLDEAHHC--T
RQHPYAVLLREFVGRAPLLPPGPPGPCRTPLMLAGPPPGPAAVSSEPGMPAAQEQPSAQE
QPPPKQRAAPVRPRLLGLTASPLEPLALQAAMCGARLITP-----VDRSGCISAHASRPK
AK---IIWVGADAP
LPPPPARQRHQCPQTLGLPSRWGPGSCPAPQAGTGASADAAAAAAVAAAAAAGGSLRALF
EAQWQEEAQAAALAPLRDCLCRCRDLVLLAEGVVTRRDRLQRLHLLEAVGWEELGLLDPN
VPPALRPHPTSGYRLCDSLRGLRLLRTQLAALEFALSDLGLWPALALAAADLFASGREAL
DGEEQGEEQGDGDLGAAAAGGGGGGEEEGDGAEAIPEPTDLGTLPSRDMAAAFVSDYGILL
ARLDESAIPLPPGLVACPRRPSLCERVAAEVLSAVMEALITVAPPQQPGEAEAIAV-GFG
EALLLRGGLRGRGRAVLRPWAVRLLRASVSPRELQRQVLGVEPPPPSAPSPSSAPTPAAM
PAPASTAGAAAAASSSAPAQQQQQQQQEQQQERSGGHVPLQLVTHRVAWLMRQLHSRVVE
AEARAAEAGAAGGVGSGSGGGGSSWSAIVFCERKSTCEALQRLITSL-PALTAAVAAGPD
TDLAPTTSPDQKQPTTVRNQSAAGPLSGSEGTMTATMAQGPLTQQMHGDNGGKEQEGGGG
GGGGGGTGPGGWQGRQQPRRPLLRSAVFVGTAGGGTAVGRHGGRLAGHKRQDAVRRAFSA
GRLNLLLSTSVGSEGLDFRSCALVAMLDLPRQVPQFVQSGGRARAQGSEHWLLVSTPEQE
AAAL--ELLASEEAMARDALTVALTNMAELLPLTPALNDGSSGNGCSLHATSTRTGTGGN
GPHHTTPAAPPPPLPPPAVPTGSRKRGGAGGAACATTVAADPASAAAAGNATGTGSAFLP
KRHRVLLNPAPVTSAVTAVPSSAHADVTTVAGTGGAPAARLEPPPPQQQHTDRRAARRHQ
QQQRLLQRLASQRRRQLGDGLTSAAAALAAAAAAAADVEEVRREGMCGDSDDGEIRGGED
GGSSGHASAGSSDDDEGVEGWADDAGDLTAEEDTGPAEVAEVAAVAATAAEAAQPKPGAL
QEQRVADGRGGAQQPRQLVDVACGEGELQNAEGSEWDPDSVAAGSFVVPGAAVCFRRAGQ
PLLCLSAGPSCAPTLGTHGPQQHPQHLPQQQPLQQPMQLPPGVSLSFPASEQPPAALLLQ
PHHRGRPLHSVVPGRSVEVMEAEAPAAAARAEVMEAEAPAAAARAEVMEAEAPAAAARAE
VMEAEAPAAAARAELMEAGAEAVAAGPEVMGAGAEAGAAGAELMEAGAEAERGDGGCDVM
MEQGENGGGTGAGDGNGGEGGGGRSGVDEEGQQQQQQQQQQQAARAAGPAGVAAAAATAT
GGEATTGEAAAAATPARSRSGKDAAGGSGSNGNASSSVFVTDERLEALEVPATGARLPAV
WAVNLLTQLCSQLPGNDGCTVLQPLCSWEASDPPPGSLGRSGVGGSSDLLGSSRARRPLL
QLRYCRCRVFLPANLGASAVLGPACRSKDAAKKSAWLAAVRLLWLRGDLDDHLRLRFTRR
AVTQAARHAELGGLAHTIPSAVLTHQLPRALLPKALLLPAEEAAATAIAATEPAVAAAAV
APPVPDTCPPQPAVAAPEDGGAVAATAPAAPSHGGAQEPPVTAMAVAVPAVAVAVADTQP
VVSPVAPDATAMDGAGDGSSASRRSTLALPTAVASDGVPSDAGISSGSSSGSPSNLSSSI
GGSSRTEPYRLHLYGFVVRPKDPHVSLLPPPGVTADGSSSCSSSSGVTRDKEAEAALAAA
AAADPAAAAGICGMLSWPTRPADEPLEPRPPPGAAAAANPAPAATPPPSTALPPSAPTGA
VAVAALDAATHATCMTAAAVAEPMTGGTVVDAPGYAVSRAAGSTQQEACADPAACDTAQQ
PKLERPQHAPLHAAIVGEYMPPPEAGMCYSSGSSSSGRPHDHSPRHGGGESCYSRQCAAA

AVHPWGVLLHGLLPAGLP-PMQLLLPEGDVDIPIEVRLVYLGPLDCGNSSSSSSSSSGSG
NATTDGGTETPADAGPPPAAAGATLPQPQPQFPPSTAAVAGGRHLPPLAHALACLAHTVL
RLMAYPCPAAIARAVVNAVAAARGEAPGAACATAEDRAAWEGFMGFGQLQQALEELLPLQ
ASPQQRPPLAGAAAGPRGGEGCCGAEAHVATAGGACGVAAPAPAQAAAAAESASPVVTAQ
NVGHDFHEAAAGAAAAHVLHGGAQQQQQPQLELPGGGYWWMPVPLRAPPAPAANTRSQQV
QEPVAPPLAAAAAGANTAPNLARPDARSGAAGALAASAEVLDRMAAELSAAAARDTGGAA
AAAALIDWRLVAELAVGPLPLSLLLPDAAAATAENDNGGASRITTAEALSSQSAKECDTV
DAGTTVVSSSSGGPGSAAAKGGRFQALAAAAAAALAAAPPAGTYAA------TPLAAPLL
DCYRRLVEPHGLVVTLDTGRLYSLPQVGTVQEPTQEALAAAAAAAAATGGGAPPRPPRPD
APRPGDAVVSAAPSYGRGRQDWLRSQKQQKEEEAAAGGEETGKRPPPSAGGA-----GAG
AQKQQQQRQGVPAGTSASAQPGAILVPSPTGPVPAPHACQSQPSTKPQPPQPPAPPPPPH
LSRRERVKAFRAPLPLLPGRLGLYPLDAGRWRALHGLPCLAWRLEGWAAAAEALTCRLEA
AGYSAGGLPPSEAEDAVESATEEAAKEEKDVMLHTPASARAPALTPGVAASAPPAAPMTP
AAPAGGGSCSGGDGTGSREGPAATAAPPLAGPCAAATTTTTTLAAAAPRITYPPPPPPTP
APLPKATAALPGEGTPAPPTYPPSSTPPIPPPPPPLLLPPPPRFTAAALSLTAAALTCPS
CRDPGVGNYDRLEFLGDAALKLLAQVYVFLRESRLPPPPHKPNPPTTHPQTLAH---HAL
GPRLRLAACLRALPLDPGRALRQAALQPDEDAASGAPLPHRLTPLPPPLMHLFSRALDAA
EAKAAEAAVEVVGSGATAAAGAATTPSATSAPATAATPGTAAATAGAGAAAPAAAVPAAA
AAVPVAAAVMPLERPSQQPPKKGSSSSPRDKERERRRLEKRAQAEQRLAATRVLGGVRGK
RLADCVEALIGAHLLQPLEGQEEEGAAEGCVVEGREEAGVEGGVEVGAAAGGSEAEGAVA
VVPGGGGGSGKDDGGGSDGGGGAWAVANTRVWSPQLQDAMQASLRFLGFAELDAAAAASQ
ARQAARAAAAAAAAVVAADARSLGGGQGTGGGGGDAAAARSVCELEALLGYRFRNPDLCV
QALTHVSHPRAAGCGGGYQRLEFLGDAVLGLVVSFWVFSAGGS--AEAMSTRRRDLVSNG
PLAEAAARLGLQRHLRAGSGALVAAVARFVDIYLAAAPGSVVVTTAERHAKNKRAEHRAQ
LRQQRQQQDREREREKEKEKEGAAAAGDEAADRADKEEAARLAGTEQSAGAGLAGSVEVV
SGREGAGAAGTALTAGPAAGMGLEDAAAAAALGRERPQKRRRRDEGNGQQQEQVQETLQE
APDTQQEVQQQGMGMEVDGATKQQQQQQPQKQQQPQQDGESDQQQQDGDKHSLQVQQRQV
DTPAGSGRKRARDGGGTAAGGTGGSGGGKKAEPRILLAPKALADLVEALVGAVYLDSGGH
LGVSERAVAVLLRLQPPPASQEAAVATVAATHVQRAVAMELYEQVISPLVVYWCRTTVYL
GIILAGPNTAPSMVAQAYLQQSQAVVAYHIIMFASLLGAWDVDARVELAAVLLEAACALT
CLSWHGAKVPGGSPHPAALLAGRALVPLVNLSLAYAAGRWRPGGLGTGRRGGGSALALDK
CISMRASGAAGGSSSKGKGKGKDMGAGPVEECAGDGDKSLGLSLMPPAEQAVRQGAEPSG
SGCGGSSSSSRTEPQPLPALPLPAPMLERSSGTVTVAAPQAAGHQSQPSAPAGSSVLRPH
KPYRSYLRGRTKHYFKFKERRVLLSQIPWAELEDITPGFEERLAALCAARGRVLTGVYVR
AGCIELTIHLAEYGAGTTRAGVHAGPAAAAGALSHSDAAVLSVEEVIAALGLSLPSGPAD
AALQAVSVQQELVDRAASEPEASGDGARSQARVVRVSPRVLLLTPADAAASSAAALRVRV
AFGGSTGPATAFNVAMLGAAGVLATRVTAKELQAASKEGAEAGSEEWEYAIELLEAPPHP
QLLTVQLAFPAAAAGAAAEDSAAAATPSPYVQWLSVPVLAVDDAAVAAELSAALQLLQTS
EAGAEVGAAAEWAHPLLCDLGLWLASAAASGAAGCSEAVRRLPAEWVSEVGACLLAYTEG
AGLVATAARIRADTQRLAALPAVAEAIAAKESPRVADDDAAVGTCQHAAASTSGSATAAM
AWQKPNTNALRQRCKGSSRGKCASKGAECGGKEEESSSSAGGGSDCSPDGLQQYTQAWIV
AMCFTCQIVECLVALGFAVRGVRAGQPLWSAESAVVYTGLGVGVVTTLAGLWMTPRAWSR
LALAARLPRFLGFMFGKALVSVGALPPPAGAATYGSGPGILLLEGVILPSGCLLPPRTAL
AMSCLKLPLNIMSGLVVGAFENLLPAVLMALAVEALAMGTTLACHGYCPPWVRHGGGMQW

ARGHLGVSVRAVAVPHAVAMEVELYEQVISPLVVYWCRTTVYLGIILAGPNTAPSMVAQA
YLQQSQAVVAYHIIMFASLLGAWDVDARVELAAVLLEAACALTCLSWHGAKVPGGSPHPA
ALLAGRALVPVVNLSLAYAAGRWRPGGLGTGRRGGGSALALDKCISMRASGAAGGSSSKG
KGKGEDMGAGPMEECADYSDMSLGLSLMPPAEQAVRQGAEPSGSGGGGSSSSCTEPQPLP
ALPLPAPMPERSSGTVTVAVPQAAGHQQSQPSAPAGSSVLRPHKPYRSYLRGRTKHYFKI
PWAEPEDITPGFEERLAALCAARGRVLTGVYVRAAAAGALSHSGPAVLSVEEVLAALGLS
LPSGPANAALQAVSVQQELVDRAASEPEASGDGSRSRVVGVSPRVLLLTPADAAASSAAV
LRVRVAFGGSTGPATASNVAMLGAAGVLATRVTAKELQAASKEGADAGSEEWEYAIELLE
APPHPQLLTVQLAFPAAAAGAAAEDSAAAATPSPYVQWLSVPVLAVDDAAVAAELSAALQ
LLQISEAGAEEGAAAEWAHPLLCDLGLWLASAAASGAAGFSEAVRRLPAEWVSELGACLL
AYTEGAGLVATAARIRADTQRLVTGAAGTVPYIHQQQQQQQQQQQEEGEAAAVGLHRRG
PLSNAAGGSGKGTGCSDDAPGHHGDGTDAKSGAGGLQDYLDSWIVTTCTTGQLTECLMFI
TFTLRGLKAGQLLWSAEAAVVYTALGMGTLVMLAGLWMPARQRSRLTLAARIPRFAAALA
SKVLVSAGVLPPPLLLCCLKLPLNAIGAFRAGAFPTLLSAALQALAVEALAMGTTLAFHS
YLRRQYRVHLQRQAAAPA
>Arabidopsis_thaliana_DCL-3_ARATH

--MHSSLEP-
---------------------------------EKMEEGGGSNSLKRKFSEIDGDQNLDS
VSSPM---MTDSNGSYELKVYE
VAKNRNIIAVLGTGIDKSEITKRLIKAMG------------SS---DTDKRLIIFLAPTV
NL---QCCE---
-----------------------IRALVN-----LKVEEYFGAK-------GVDKWTSQR
WDEEFSKH--------------DVLVMTPQILLDVLRSAFLKLEMVCLLIIDECHHT--T
GNHPYAKLMK--

---IFNPEEREG
V----------------------EKFATTVK-----------------------------
EGPILYNP----SPSCSLELKEKLETS---------HLKFDAS----------LRRLQEL
GKDSFLNMDNKFETYQ------KRLSIDYREILHCLDNLGLI--CAHLAAEVCLEKISDT
KEESETYKECSM--
----------------------VCKEF------------------------------LED
ILSTIGVYLPQ-----DDKSLV--------------------------------------
----------------------------DLQQNHLSAVISGHVSPKLKELFHLLDSFRGD
KQKQ----------------------CLILVERIITAKVIERFVKKEASLAY--------

----------------------LNVLYLTENNPSTNVSAQ--------KMQIEIPDLFQH
GKVNLLFITDVVEEGFQVPDCSCMVCFDLPKTMCSYSQSQKHAKQSNSKSIMFLERGNPK
QRDHLH------DLMRREVL--

---IQDPE
A-----PNLKSCPPPVK---

---NGHGVKEIGSMVIPD
SNITV---
----------SEEAASTQTMSDPPSRNEQLPP-------CKKLR----LDNN--------
------------------------------------LLQ---------------------
--SN
GKEKVASSKSKSSSSAAGSKKRKELHGTTCANALS-----------GTWGEN--------
-----IDGATFQAYKFD---
----------------FCCNISGEV-----------------------------------

-YSSFSLLLESTLAEDVG-KVEMDL-YLVRKLVK-ASVSPCGQIRLSQEELVKAK-----
---YFQQFFF
NGM---

-----FGKLFVGSKSQGTKREFLLQTDTSSLWHPAFMFLLLPVETNDLASSAT-------

-----IDWSAIN---------SCASIVEFLKKNSLLDLRDSD------------------
-------------GNQCNTSS----GQEVLLDDKMEET--NLIHFA-------------N
ASSDKNSLEELVVIAIHTGRIYSIVEA---------------------------------
------------------------------------VSDSSAMSPFEVDASS----GYAT
YAEYFNK------------KYGIVLAHPNQPLMKLKQSHHAHNLLVDFNEEMVVKTEPKA
GNVRKRKPNIHAHLPPEL--LARIDVPRAVLKSIYLLPSVMHRLESLMLASQ--------
---LREEIDCS-IDNFSISS

---TSILEAVTTLT
CPES--FSMERLELLGDSVLKYVASCHLFLKY----PDKDEGQLSRQRQSIISNSNLHRL
TTSRKLQGYIRNGAFEPRRWT---------------------------------------
------------------------------------APG-----------QFSLFPVPCK
CGIDTREVPLDPKFFTENMTIKIGKSC--------------DMGHRWV---------VSK
SVSDCAEALIGAYY-------------------------VSGG-----------------
---------------------------------------LSASLHMMKWLGIDVDFDPNL
VVEAINRVSLRCYIPKEDE----------------------LIELERKIQHEFSAKFLLK
EAITHSSLRE----SYSYERLEFLGDSVLDFLITRHLFNTYEQTGPGEMTDLRSACVNNE
NFAQVAVKNNLHTHLQRCATVLETQINDYLMSF---------------------------

---------------QKPDET-----GRSIPSIQ---GPKALGDVVESIAGALLIDTR--
--------------------------------LDLDQVWRVFEPLLSPLV----------

------------TPDKLQLPPY--------------------------------------
--RELNELCDSLG---------

--YFFRVK---CSNDG
VKAQATI---
---QLQLDDVLLTG----
----DGSEQTNKLALGKAASHLLTQL----------------------------------
------------------------E-------KRNISRKTSLGDNQSSMDVNLACN----

--------------------HSDRETLTSETTEIQSIV----------------------

---IPFIGPI
NMKKGGPR--

-------------GTLHEFCKKH-----------------LWPMPTFDTSEEKSRTPFEF
IDGGEKRTSFSSFTSTITLRIPNREAVMYAGEARPDK-----------------------

-------------------------KSSFDSAVVELLYELERRKIVIIQK----------

>Populus_trichocarpa_DCL-3_POPTR

---YQSKVFE
VAVKRNTIAVLETGAGKTMIAVMLIKQIG------------QAVFYSGVKRLILFLAPTV
HLVNQAC-F---
-----------------------IKSQTN-----FRVGEYYGAK-------GIDEWSLKS
WEKEIDEH--------------DVLVMTPQILLDALRKAFLNLKMVSLLILDECHRS--T
GNHPYKKIMK--

---IYTIEDRAE
V----------------------HVYVPSAK-----------------------------

ELCRFYDK----AWCSYVELKDKIEAS---------WSKFDAS----------MLALQGS
TQSCYKDMDDKLKATR------KQLSKDHAKILNCLEDLGLI--CAYEAIKVCLENAGNP
TGECKLYQEISL--
----------------------QCRYF------------------------------LED
VLHIIGESLLH-----GDNFSL--------------------------------------
--------------------------DHGFDCSAALGF--GYISPKLHELLQLFLSFGEA
REVL----------------------CLIFVERIITAKVVERFMKKV-EVLA-H------

----------------------FTVSYLTGTNASADALAP--------KMQMETLESFRS
GKVNLLFATDVVEEGIHVPNCSCVIRFDLPKTVRSYVQSRGRARQNNSHFITMLERGNTK
QRDQLFEIIRSEWSMTDTAI--

---NRDPN
V-----WNLKACASEAA---

---KAYVVDVTGASVTAD
SS--
----------LPPNAAFQTLVGPTCRNQQLAKQLVCLEACKKLHQMGALDDH--------
------------------------------------LLPSVE------------------
--EP
SEIAVVKS--KSTSAGAGTTKRKELHGTACIHALS-----------GSWGEK--------
-----LDGATFHAYKFD---
----------------FSCSIVSQI-----------------------------------

-YSGFILLIESKLDDDVG-NIELDL-YLVAKIVK-SSISSCGVVHLDAAQMTKAK-----
---RFQEFFF
NGL---

-----FGKLFT---GSKSSREFLLQKETTLLWSPSNMYLLLPLEPWSISSNDWCK-----

-----IDWKGIE---------ACSSVVEYL-KNSFLA-----------------------
-------------ARSYSGGG--NPLPDNVQSSTIECNGTNLIHFA-------------N
ALVNVENIKDMVVLAIHTGRIYSIVKV---------------------------------
------------------------------------VNDSSAESAFEGNADN--VTEFST
YTEYFNK------------RYGIVLMHPGQPLLRLKQSHNPHNHLVNFNDEGLLVCDSKD
-GMVGRKQQQHVHMPPEL--LIKIDVPISVVKSIYLMPSLMHRLECLMLASQ--------
---LRQEIDCH-APNFYIPS

---SLILEAITTLR
CCES--FSMERLELLGDSVLKYAVSCHLFLKY----PNKHEGQLSSWRSGAVCNSTLHKL
GTDCKVQGYILDSAFDPRRWA---------------------------------------
------------------------------------APG----------QKSVRTPAPCK
CGVDTLEVPLDRKFQTESAIVKVGKPC--------------DSGHRWM---------GSK
TISDCVESVIGAYY-------------------------VSGG-----------------
---------------------------------------LIAAIHVMKWFGINAELDPSL
ISEAITSASLRSYIPKEDE----------------------IKSLESKLGYTFGVKFVLQ
EAMTHASIQE-QGVTYCYQRLEFLGDSVLDLLITWHLYQSHTDVDPGELTDLRSASVNND
NFAQVAVKQNLYTHLLHCSTLLQSQITEYVNSF---------------------------

--------------------------HESDQGTK---APKALGDLIESIAGALLIDTK--
--------------------------------FNLDGVWRIFKPLLSPIV----------

------------TPEKLELPPL--------------------------------------
--RELVELCDSIG---------

--VFVKEK---CTKKA
EMVHAQL---
---WVQLDNELLSG----
----EGYEKNRKAAKGKAASCLLKKL----------------------------------
------------------------Q-------VCNSGNCNHAFFCFLMNSIVPSNI----

--------------------VEYLAKSPPFAV----------------------------

--IESI
NKKKGGPR--

-------------TSLYDLCKKV-----------------QWTMPTFDTTETKSRTAIEF
GEGPDKRTGFNSYVSKIIMNIPSYGVVECAGEASADK-----------------------

-------------------------KTSYDSAALAMLNELEKRGQLIIDE----------

>HbDCL3

---MKAEKEINVRMCRGHVN
KGIHY---------------------------------------LISSVVNGYYQLKVFE
VAMKRNTIAVLETGAGKTMIAVMLIKEIG------------QAIKFTDHKKLILFLAPTV
HLVNQQYEV---
-----------------------IKANTS-----FDAGAYYGAK-------GVDGWTLKL
WEKEIDEH--------------DVLVMTPQILLDALRKAFLNLDMVSLMIIDECHRA--T
GNHPYTKIMK--

---VYTIEDRTE
M----------------------DIHVPSAR-----------------------------
ETCRFYDK----AQCYSSELKSKIEAS---------WSKFDAS----------MLSLQGS
IQSGYKDIDDKIRTLR------QRLSNDHAKILNCLEDLGLI--CAYEAVKVCLQSAPNS
TEDCVIYREISL--
----------------------QHRYF------------------------------LEE
VLSLIGESLPH-----CDNFLL--------------------------------------
--------------------------DLDFDFLKAVVF--GYISPKLYELLQLFLSFGGA
RQIL----------------------CLIFVDRIVTAKVIERFVKKV-AVLA-H------

----------------------FTVSYVTGTNTSVDAVTP--------KTQKETLESFRS
GKVNLLFSTDVLREGIHVPNCSCVICFDLPKTVCCYVQSRGRARQNDSEYIIMLERGNVK
QRDQLFDFIRSEWLMTNTTI--

---NRDPD
V-----QSLKTCAAEET---

---KAYIVNATGASVTAD
SSVNLIYRYCEKLPG-D---
-------------SAAFQTLVGPICRSQQLAKQLVCLEACKKLHQMGALDDH--------
------------------------------------LRPSFD------------------
--DP
MEDNCIMRSKETLAGAGTT-KRKELHGTTGIHALS-----------GSWGEN--------
-----LDGTTFYAYKFD---
----------------FSCSIATEM-----------------------------------

```
------------------------------------------------------------
-YSGFILLIESKLDDDVG-NVELDL-YLVRKIVR-ASVSSCGQVHLDAEQMMKAK-----
-----------------------------------------------------CFHELFF
NAL---------------------------------------------------------
------------------------------------------------------------
-----FGKLFTGS-KSSKTRELLLQKEKSLFWIPSNMYLLLPLETFSASNDESWK-----
------------------------------------------------------------
-----INWSGVD---------SCTYVVEFL-KNSFLG-----------------------
-------------AEQCNGES-RRSSSCRVGSSVTECSGVNTIHFA-------------N
FSVDADKLKDVVVLAIHTGKIYSVIEV---------------------------------
------------------------------------VSDMSAESPFEQNAGD-APSNYSS
FTEYFNK------------KYGIVLMHPGQPLLLLKQSHKPHNLLPNPNDE-----VTSK
DGMAVVKQRQFVHMPPEL--LVSIDIPVQTLKSFYLLPSLMYRLESLMLASQ--------
-------------------------------------------LRQEIDCR-IPDCYIPS
------------------------------------------------------------
-------------------------------------------------SLMLEAITTLR
CSET--FSMERLELLGDSVLKYAISSDLFLRY----PTKHEGQLSARRIQAVCNSALHKL
GISLKLQGYIRDSAFNPRCWV---------------------------------------
-----------------------------------APG----------QRPAYY-IPCK
CGVDTLEVPLDAKFQTEDPKIKIAICC--------------SMGHRWI---------CSK
TISDCVEAILGAYY-------------------------VSGG-----------------
---------------------------------------LIAAVHVMKWLGMDIEYDPSL
VDEAISSASLRSYMPKENE----------------------VTSLESKLGYTFSVKFLIQ
EAMTHASMQE-QGVGYCYQRLEFLGDSLLDLLITWHLYQSHTNIDPGELTDLRSACVSNE
NFAQIVVRRELYKHLQHCSTLLLSQITEYLESF---------------------------
------------------------------------------------------------
------------------------------------------------------------
------------------------------------------------------------
---------------CESDEA-----TKLSTVPK---GPKALGDLLESIAGAMLVDTK--
--------------------------------FDLDEVWRIFKPLLSPIA----------
------------------------------------------------------------
------------------------------------------------------------
------------------------------------------------------------
------------TPESLELPPL--------------------------------------
----------------------------------------RELIELCDSHG---------
------------------------------------------------------------
------------------------------------------------------------
----------------------------------------------YFIKER---CTIKN
DIVHAHL-----------------------------------------------------
---------------------------------------------RLQLNDVLLVG----
----DGYDRNRKTAKGKAASHLLKEL----------------------------------
------------------------E-------NRGIRYSRGDSKRKQDPDHVVDSS----
------------------------------------------------------------
--------------------SLDLTNAHTWSSGVPSLVGSPKEGLPVIES----------
```


---I
NMKKGGPR--

-------------TTLFELCKKG-----------------EWTRPTFQTTETKSRTPIIF
GEGSERRESFISFISKITLNIPFYGIIECAGDPRPDK-----------------------

-------------------------KSSFDSAALAMLYELKELGRLIIVDAQ--------

>Ricinus_communis_DCL-3_RICCO

---MDTPMEQDRNP
LKRSFNEMNNLQDFVDMGLNGT-----------NHHHDHDAASQKPDSTITTSTTTGKGF
NP---RSYQVKVFE
VAMKRNTIAVLETGAGKTMIAVMLIREIG------------QAIKSNDFKKLIIFLAPTV
HLVNQQYEV---
-----------------------IKDNTN-----FKVGEYYGAK-------GIDGWSMKV
WEKEINEH--------------DVLVMTPQILLDALRKAFLNLEMVSLMIIDECHRT--T
GNHPCTKIMKEFYHKLS---
----------NKPKIFGMTASPVVQKGVSSA--------------VDCEDQISELETILD
SQ---IYTIEDRTE
M-----------------------DVHPSAR-----------------------------
EICKFYDIAQL----YSSDIKSKIEAS---------WSKFDAS----------LLSLQGS
IQSSYKDADDRYKTLQ------QRLYNDYSKILNCLEELGLI--CAYEAVKICLENSP-S
TAAFDIYREISL--
---------------------KCKYF-------------------------------LEE
VLSIIGTYLPH-----GDSYLL--------------------------------------
--------------------------DLGFDYLKAVRL--SYISPKLYELLQVFLSFGVD
KEVL----------------------CLIFVDRIITAKVIERFVKKVAALVH--------

----------------------FTVSYVTGSNTSVDALTP--------KTQRETMYAFRS
GKVNLLFSTDVLREGIHVPNCSCVICFDLPKTVCCYAQSRGRARQNDSQYIIMLQRGNVK
QRDQLFDFIRSEWLVTNTAI--

---NRDPD
V-----WTLKTCVPEET---

---EAYIVDVTGASVTAD
SSVTLIQRYCSKLPS-DRYFTPRPTFEFECFEQSF-----Q-------------------
------CKMMLPASAAFQTIVGPVCRSQQLAKQLACLEACKQLHQMGALDDH--------
------------------------------------LLPSVE------------------
--EP
TEDDHTVRRKGSLAGAGTT-KRKELHGTTPIHALS-----------GSWGEN--------
-----LDGATFHAYKFE---
----------------FSCSIVSEK-----------------------------------

-YSGYILLIESKLDDDVG-NIELDL-FLVRKTVR-ASVSSCGQVHLDAEQMMKAK-----
---CFHELFF
NAL---

-----FGKLFSGSKSSRSPREFLLQKETSLLWILSNMYLLLPLEAFSASSDESWK-----

-----INWRGVN---------GCSNVVEFLKKNCFLG-----------------------
-------------AEHYNGER--------VRSSVTECSGGNAVHFA-------------N
ISVDVERVKDMVVLAIHTGKIYSVVEV---------------------------------
------------------------------------VGDASAESPFEQNVGG-APSKFST
FSEYFNK------------KYGIVLLRPRQPLLLLKQSHKPHNLLNPPDEA---------
------KQRQFARMPPEL--LVSIDVPIQTLKSSYLLPSLMYRLESLMLASQ--------
---LRQEIA-----------

---CQILEAITTPR
SCEK--VSMERLELLGDSVLKYSVSCDLFLRY----PTKHEGQLSAQRTRAVCNSTLHQL
GIGCKLQGYIRDSAFIPRYWV---------------------------------------
------------------------------------APG----------QQPAFY-VSCT
CGVDTLEVPLDAKFQTEDPKVQIAICC--------------SKGHRWM---------CSK
TISDCVEAVIGAYY-------------------------VSGG-----------------
---------------------------------------LMAALHVMQWLGMDVEFDPSL
VDKIIKTASVRSYTPKEDE----------------------IKGIESKLGYTFSVKFLLQ
EAMTHASMQE-QGIGYCYQRLEFLGDSVLDLLITWHLYQNHTSMDPGELTDLRSACVSNE
NFAQVVVRRDLYKHLQHCSTLLLSQIKEYLESF---------------------------

---------------HESDEV------AKATGPK---GPKALGDLLESIAGAMLIDAK--

--------------------------------LNLDEVWRIFMPLLSPLA----------

------------TPQNLELPPL--------------------------------------
--RELMQLCNSLG---------

--YFIKEK---CINNN
DIVHAEL---
---RLQLNDVLLVG----
----NGCDRSRKAAKGKAATHLLKKL----------------------------------
------------------------E-------NRGITYSWGHSKRRKQDSNHVVDS----

--------------------------SPLDIT----------------------------

NDKAKDHKKPKKLENQSPAGSGGDPSFAGSSGENPPG-----------------------

-------------------------------------MYLNSSSPL--------------

>Arabidopsis_thaliana_DCL-4_ARATH

-MRDEVDLSLTIPSKLLGKRDREQKNCEEEKNKNKKAKKQQKDPILLHTSAATHKFLPPP
LTMPYSEIGDDLRSLDFDHADVS----------SDLHLTSSSSVSSFSSSSSSLFSAAGT
DDPSP-----------------------------KMEKD-------PRKIARRYQVELCK
KATEENVIVYLGTGCGKTHIAVMLIYE-L------------GHLVLSPKKSVCIFLAPTV
ALVEQQAKV---
-----------------------IADSVN-----FKVAIHCGGK-------RI-VKSHSE
WEREIAAN--------------EVLVMTPQILLHNLQHCFIKMECISLLIFDECHHAQQQ
SNHPYAEIMKVFY---
-----KSESLQRPRIFGMTASPVVGKGSFQS--------------ENLSKSINSLENLLN
AK---VYSVESNVQ

L----------------------DGFVSSPL-----------------------------
VKVYYYRSALSDASQSTIRYENMLEDI-----------KQRCL--------ASLKLLIDT
HQT------QTLLSMK------RLLKRSHDNLIYTLLNLGLW--GAIQAAKIQLNSDH--
--NVQDEPVGKN-PKS--
---------------------KICDTYL-----------------------------SMA
AEALSSGVAKD---
--------------------------ENASDLLSLAALKEPLFSRKLVQLIKILSVFRLE
PHMK----------------------CIIFVNRIVTARTLSCILNNL-ELLR-S------

----------------------WKSDFLVGLSSGLKSMSR--------RSMETILKRFQS
KELNLLVATKVGEEGLDIQTCCLVIRYDLPETVTSFIQSRGRARMPQSEYAFLVDSGNEK
EMDLIENFKVNEDRMNLEIT--

---YRSSE
E---------TCPRLDE---

---ELYKVHETGACISGG
SSISLLYKYCSRLPH-DEFFQPKPEFQFKPVDEFGGT---I-------------------
------CRITLPANAPISEIESSLLPSTEAAKKDACLKAVHELHNLGVLNDF--------
------------------------------------LLPDSK------------------
--DE
IEDELSDDEFDFDNIKGEGCSRGDLYEMRVPVLFK-----------QKWDPS--------
-----TSCVNLHSYYIM---
----------------FVPHPADRI-----------------------------------

-YKKFGFFMKSPLPVEAE-TMDIDL-HLAHQRSVSVKIFPSGVTEFDNDEIRLAE-----
---LFQEIAL
KVL---

-----FER------GELIPDFVPLELQDSSRTSKSTFYLLLPLCLHDGESVIS-------

-----VDWVTIR---------NCLSSPIFK------------------------------
-------------TPSVLVED------------IFPPSG-SHLKLA-------------N
GCWNIDDVKNSLVFTTYSKQFYFVADI---------------------------------
------------------------------------CHGRNGFS--PVKESS-----TKS
HVESIYK------------LYGVELKHPAQPLLRVKPLCHVRNLLHNRMQT---NLEPQE
------LDEYFIEIPPELSHLKIKGLSKDIGSSLSLLPSIMHRMENLLVAIE--------
---LKHVLSASIPEIAEVSG

---HRVLEALTTEK
CHER--LSLERLEVLGDAFLKFAVSRHLFLHH----DSLDEGELTRRRSNVVNNSNLCRL
AIKKNLQVYIRDQALDPTQFF---------------------------------------
------------------------------------AFG-----------HPCRVTCDEV
ASKEVHS--LNRDLGILESNTGEIRC---------------SKGHHWL---------YKK
TIADVVEALVGAFL------------------------VDSG------------------
---------------------------------------FKGAVKFLKWIGVNVDFESLQ
VQDACIASRRYLPLTTRNN----------------------LETLENQLDYKFLHKGLLV
QAFIHPSYNR-HGG-GCYQRLEFLGDAVLDYLMTSYFFTVFPKLKPGQLTDLRSLSVNNE
ALANVAVSFSLKRFLFCESIYLHEVIEDYTNFL---------------------------

---------------ASSPLA-----SGQSEGPR---CPKVLGDLVESCLGALFLDCG--
--------------------------------FNLNHVWTMMLSFLDPVK----------

------------NLSNLQISPI--------------------------------------
--KELIELCQSYK---------

--WDREIS---ATKKD
GAFTVEL---
---KVTKNGCCLTV----
----SATGRNKREGTKKAAQLMITNL----------------------------------
------------------------K-------AHENITTSHPLEDVLKNGIRNEAK----

--------------------LIGYNEDPIDVV---DLVGLDVENLNILET----------

----------------------FGGNSERSSSYVIR------RGLPQAPSKTEDRLPQKA
IIKAGGPSSK-------------------TAK----------------------------

-------------SLLHETCVAN-----------------CWKPPHFECC----------
EEEGPGHLKSFVYKVILEVEDAPNMTLECYGEARATK-----------------------

-------------------------KGAAEHAAQAAIWCLKHSGFLC-------------

>Populus_trichocarpa_A_DCL-4_POPTR

--MNVILERFRT
GKLNLLLATKVGEEGLDIQTCCLVIRFDLPETVASFIQSRGRARMPQSEYVFLVDSGNQK
ERDLIEKFKIDEARMNIEIC--

---DRTSR
E---------TFDSIEE---

---KIYKVHATGASITSG
LSISLLQQYCSKLPH-DEYFDPKPKFFY--FDDSEGT---V-------------------
------CHIILPSNAPTHKIVGTPQSSIEVAKKDACLKAIEQLHKLGALSEF--------
------------------------------------LLPQQE------------------
--DT
NELELVSS--DSDNCEDKD-SRGELREMLVPAVLK-----------ESWTEL--------
-----EKPIHLNSYYIE---
----------------FCPVPEDRI-----------------------------------

-YKQFGLFLKAPLPLEAD-KMSLEL-HLARGRSVMTKLVPSGLSKFSTDEITHAT-----
---NFQELFL
KAI---

-----LDR------SEFVHEYVPLGKDALSKSCPT-FYLLLPVIFHVSERRVT-------

-----VDWEIIR---------R---------------------------------------
--------------SNAVDKG------------ILPSN--DCLQLA-------------N
GCSSIRDVENSLVYTPHQKKFYFITNI---------------------------------
------------------------------------VPEKNGDS--PCKGSN-----TRS
HKDHLTT------------TFGIHLRYPEQPLLRAKQLFCLRNLLCNRKKE---DSELQE
------LDEHFVDLAPELCELKIIGFSKDIGSSISLLPSV--------------------

--------FIRDQPFDPYQFF---------------------------------------
------------------------------------ALG-----------HPCPRICTKE
SEGTIHS--QCGSHVTGQAKGSEVRC---------------SKGHHWL---------HNK
TVSDVVEALIGAFL-------------------------VDSG-----------------
---------------------------------------FKAAIAFLRWIGIKVDFDDSQ
VINICQASRTYAMLNPSMD----------------------LATLENLLGHQFLYKGLLL
QAFVHPSH-K-NGG-GCYQRLEFLGDAVLDYLITSYLFSVYPKMKPGHLTDLRSVLVNNR
AFASVAVDRSFHEYLICDSDALSAATKKFVDFV---------------------------

---------------RTPKSE-----RRLLEGPK---CPKVLGDLVESSVGAILLDTG--
--------------------------------FDLNHIWKIMLSFLNPIS----------

------------SFSNLQINPV--------------------------------------
--RELKELCQSHN---------

--WDFEVP---ASKKG
RTFSVDV---
---TLSGKDMNISA----
----SASNSNKKEAIRMASEKIYARL----------------------------------
------------------------K-------DQGLIPMTNSLEEVLRNSQKMEAK----

--------------------LIGYDETPIDVAL--DAHGF--ENSKIQEP----------

----------------------FGIN----CSYEVR------DSCPPRFEAVDAWSLSPL
DFTGGQPSEATGDLRCDRDVLITGKVDLGTAR----------------------------

-------------SRLREICAAN-----------------SWKPPSFECC----------
TEEGPSHLKSFTYKVVVEIEEAPEMSFECVGSPQMKK-----------------------

-------------------------KAAAEDAAEGALWYLKHQRHLS-------------

>Populus_trichocarpa_B_DCL-4_POPTR

----------------MSGGHVT----------GEHSSLSVGGTNARVVSSSIVGDGEES
GSGLQ-----------------------------KTEKD-------PRKMARKYQLELCK
KALEENIIVYLGTGCGKTHIAVLLIYE-M------------GHLIRQPQKSACVFLAPTV
ALVHQQAKV---
-----------------------IEDSTD-----FKVGIYCGKS-------NR-LKTHSS
WEKEIEQN--------------EVLVMTPQILLYNLSHSFIKMDLIALLIFDECHHAQVK
SGHPYAQIMKVFY---
-----KNNDGKLPRIFGMTASPVVGKEKYRERVTSLEILLHHLIRENLPRSINSLENLLD
AK---VYSVEDKEE
L----------------------ECFVASPV-----------------------------
IRVYLYGPVANGTSSSYEAYYNILEGV-----------KRQCI--------VEIGKKTDG
NQSL-----ESLRSTK------RMLIRMHENIIFCLENLGLW--GALQACRILLSGDH-S
EWNALIEAEGNT-SDV--
---------------------SMCDRYL-----------------------------NQA
TNVFAADCTRD---
--------------------------GVTSNVSQVEVLKEPFFSRKLLRLIEILSNFRLQ
PDMK----------------------CIVFVNRIVTARSLSHILQNL-KFLT-S------

----------------------WKCDFLVGVHSGLKSMSR--------KTMNVILERFRT
GKLNLLLATKVGEEGLDIQTCCLVIRFDLPETVASFIQSRGRARMPQSEYVFLVDSGNQK
ERDLIEKFKIDEARMNIEIC--


```
------------------------------------------------------------
------------------------------------------------------------
-------------------------------------------------------DRTSR
E---------TFDSIEE-------------------------------------------
------------------------------------------------------------
------------------------------------------------------------
------------------------------------------------------------
---------------------------------------------KIYKVHATGASITSG
LSISLLQQYCSKLPH-DEYFDPKPKFFY--FDDSEGT---V-------------------
------CHIILPSNAPTHKIVGTPQSSIEVAKKDACLKAIEQLHKLGALSEF--------
------------------------------------LLPQQE------------------
----------------------------------------------------------DT
NELELVSS--DSDNCEDKD-SRGELREMLVPAVLK-----------ESWTEL--------
-----EKPIHLNSYYIE-------------------------------------------
----------------FCPVPEDRI-----------------------------------
------------------------------------------------------------
------------------------------------------------------------
-YKQFGLFLKAPLPLEAD-KMSLEL-HLARGRSVMTKLVPSGLSKFSTDEITHAT-----
-----------------------------------------------------NFQELFL
KAI---------------------------------------------------------
------------------------------------------------------------
-----LDR------SEFVHEYVPLGKDALSKSCPT-FYLLLPVIFHVSERRVT-------
------------------------------------------------------------
-----VDWEIIR---------RCLSSPVF--KN---------------------------
-------------PANAVDKG------------ILPSN--DCLQLA-------------N
GCSSIRDVENSLVYTPHQKKFYFITNI---------------------------------
------------------------------------VPEKNGDS--PCKGSN-----TRS
HKDHLTT------------T------------------------------------ELQE
------LDEHFVDLAPELCELKIIGFSKDIGSSISLLPSVMHRLENLLVAIE--------
-------------------------------------------LKCILSASFSEGDKVTA
------------------------------------------------------------
-------------------------------------------------HRVLEALTTEK
CQER--LSLERLETLGDAFLKFAVGRHFFLLH----DTLDEGELTRKRSNAV--------
--------FIRDQPFDPYQFF---------------------------------------
------------------------------------ALG-----------HPCPRICTKE
SEGTIHS--QCGSHVTGQAKGSEVRC---------------SKGHHWL---------HNK
TVSDVVEALIGAFL-------------------------VDSG-----------------
---------------------------------------FKAAIAFLRWIGIKVDFDDSQ
VINICQASRTYAMLNPSMD----------------------LATLENLLGHQFLYKGLLL
QAFVHPSHK--NGGEFGVMILQFAMTLMFPPEIGVPWRCFYPKMKPGHLTDLRSVLVNNR
AFASVAVDRSFHEYLICDSDALSAATKKFVDFV---------------------------
------------------------------------------------------------
------------------------------------------------------------
------------------------------------------------------------
```

---------------RTPKSE-----RRLLEGPK---CPKVLGDLVESSVGAILLDTG--
--------------------------------FDLNHIWKIMLSFLDPIS----------

------------SFSNLQINPV--------------------------------------
--RELKELCQSHN---------

--WDFEVP---ASKKG
RTFSVDV---
---TLSGKDMNISA----
----SASNSNKKEAIRMASEKIYARL----------------------------------
------------------------K-------DQGLIPMTNSLEEVLRNSQKMEAK----

--------------------LIGYDETPIDVAL--DAHGF--ENSKIQEP----------

----------------------FGIN----CSYEVR------DSCPPRFEAVDAWSLSPL
DFTGGQPS----------------KVDLGTAR----------------------------

-------------SRLREICAAN-----------------SWKPPSFECC----------
TEEGPSHLKSFTYKVVVEIEEAPEMSFECVGSPQMKK-----------------------

-------------------------KAAAEDAAEGALWYLKHQRHLS-------------

>HbDCL4

---------------------------------------MSKKLPYRREVNYEIELELGA
YLQPW--YQLELCK
KAMEENIIVYLGTGCGKTHIAVLLIYE-L------------GHLIRKPQKNVCVFLAPTV
ALIQQQAKV---
-----------------------IEESTD-----FKVGIFYGS--------SKHLKNHYD
WEKEIEQY--------------EVLVMTPQILLHTLSHSFIKMELISLLIFDECHHAQVK
SNHPYAEIMRVFY---
-----KANDGKLPRIFGMTASPVVGQGASSQ--------------ANLPRSINSLENLLD

AK---VYSVEDKEE

L----------------------EHCVASPV-----------------------------

VKIYEYGPVANGTSSYYMTYCSKLEEI-----------KHTCI--------SELHKKVDE

CQSV-----QGLRNTK------KVLSRMHDNVVFCLENLGLW--GALQACQILLSGDP-S

EWNALIEEEGNI-SND--

---------------------SFCDRYL-----------------------------AQA

ANFLAAVCTTD---

--------------------------GVESDISLVEVLNEPFFSRKLLRLIGILCNFRLQ

PNMK----------------------CIIFVNRIVTARSLSYILQNL-KFLT-S------

----------------------WKCDFLVGVHSGLRSVSR--------KTMNNILEKFRT

GKLNLLIATKVGEEGLDIQTCCLVIRFDLPETVASFIQSRGRARMPQSEYAFLVDRGKQK

ELDLIESFKYNEDQMNREIA--

---SRTSN

E---------TFVGLEE---

---KVYKVDISGASVSSA

YSISLLHRYCSKLPH-DEYFDPMPKFFY--FDDLGGT---I-------------------

------CHIILPSNAPVHQIVGTPQPSQEAAKKDACLLAIEQLHQLGALNNF--------

------------------------------------LLPHQE------------------

--DA

DEESVLLSS-DSDNCEDEG-SRGELHEMLVPALLK-----------ESWTAS--------

-----ENWIHLHSYYIE---

----------------FCPFPKDRT-----------------------------------

-YEKFGLFIKAPLPQEAE-QMQLDL-HLARGRIVTTKLVLSGVMKFNTDEITMAM-----

---NFQEMFL

KVI---

-----LDR------KELISEFVPLGKDSFCKSSHT-FYLLLPVILRGCGSGVT-------

-----VDWEVIR---------RCLLSPVF-------------------------------

---------------RSVQKE------------NFPSV--AHLQLA-------------N

GCRSMRDIENSLVYIAHKKQFYFVSNI---------------------------------

------------------------------------FHGKNGFS--PHKGSS-----TPS

HLEHLIQ------------TFGIQLKYPEQPLLQAKPLFCLHNLLHNRRQE---DSETQE

------LDEYFIDVPAELCELKIIGFSKDIGSSVSLLPSIMHRLENLLVAIE--------

---LKSLLSGSFSEGAKVTA

---FRVLEALTTEK
CQER--LSLERLEVLGDAFLKFAVGRHLFLLH----DTLDEGELTRKRSNAVNNSNLLKL
ARRKNLQVYIRDQPFDPCQFF---------------------------------------
------------------------------------ALG-----------RPCHIICTKE
SEGNIHS-----------QNANGVRC---------------SKGHHWL---------HRK
TIADVVEALVGAFI-------------------------VDSG-----------------
---------------------------------------FKAATAFLKWLGIRVDFEASE
VIKVCLASRSFMPLAPSID----------------------NVDLEKLLGHRFLHKGLLL
QAFVHPSYNK-HGG-GCYQRLEFLGDAVLDYLITSYLFSVYPKLKPGHLTDLRSALVNNR
AFANVAIDRSLHKFLLCDSSNLSEAIKNYVDSV---------------------------

---------------KTPASG-----RGLLEGPK---CPKDRGYSRKS------------
--------------------------------NYLEEVLRSSQKMEAKLI----------

---------------GYDETPI--------------------------------------
---DIAASCATTT---------

--LENLKV---QEPVG
SNFNPKI---

----YSMSASKQPASHLAVGGQ--------------------------------------
------------------------P-------SETVAYSRSDTDSLAKDGSHVRSA----

------------KSRLHEICAAN-----------------CWKPPSFECC----------
FEEGPSHLK---

>Ricinus_communis_DCL-4_RICCO

----------------MPDGEAD----------ISPDDSSSICTVNRIAGDD---DGDEC
NSMPQ-----------------------------QPEKD-------PRKIARKYQLELCK
KALEENIIVYLGTGCGKTHIAVLLIYE-L------------GHLIRKPLKNVCVFLAPTV
ALV-QQVRV---
-----------------------IEQSID-----FKVGVYCGNS-------NH-LKSHRD
WEKEIEQN--------------EVLVMTPQILLHTLGHSFIKMELISLLIFDECHHAQVQ
SSHPYAEIMKVFY---
-----KTGDGKFPRIFGMTASPVVGKGASNQ--------------ANLPKSINSLENLLD
AK---VYSVEDNEE
L----------------------ELFVASPV-----------------------------
VRIYLYAPVANEKSSSYMTYFSKLEEI-----------KRKCL--------LELHKKADS
CQSL-----HGLQNAK------KVFIRMHDNVVFCLENLGFW--GALQACKILLSDDH-F
EWNALIEAEGNI--DA--
---------------------SVCDKYL-----------------------------AQA
ANMFASVCTKD---
--------------------------CIAFDLSSVEVLTEPFFSRKLLRLIGILSTFRLQ
PNMK----------------------GIVFVNRIVTARSLSYVLQNL-KFLI-S------

----------------------WKCDFLVGVHSGLKSMSR--------KTMNSILEKFKT
GKLNLLIATKVGEEGLDIQTCCLVVRFDLPETVASFIQSRGRARMPQSEYAFLVDSGNQK
ELDLIERFRRDEDRMNMEIS--

---SRTSN
E---------TFVSIEE---

---KVYKVDESGACISSA
YSISLLHHYCSKLPH-DEYFDPKPQFFF--FDDLGGT---I-------------------
------CHIILPANAPVHQIVGTPQSSREAAKKDACLKAIEQLHKLGSLSNF--------
------------------------------------LLPHEK------------------
--DV
NEESMLASS-EPENNEGEG-VRGELHEMLVPAVFK-----------ESLTSS--------
-----ENWINLHSYFIK---

----------------FCPVPEDRI-----------------------------------

-YKKFGLFIRAPLPLEAE-QMELNL-HLACGRYVATKLVPLGCLAFHRDEITQAI-----

---YFQEMFL

KVI---

-----LDR------SDFVPEFVTLGKNSFFESSPS-FYLLLPVLLCDHGNRVT-------

-----VDWETVG---------RCLSSPVF-------------------------------

---------------RCVEKE------------CLPSD--DCLQLA-------------N

GCRSIRDIENSLVYIPHKKHFYFITNI---------------------------------

------------------------------------DRGKNARS--PHKCSS-----TSS

YMEFLIQ------------RFGIQLKYPEQPLLQAKPLFSLHNLLHNRRKE---DSVTQE

------LDEYLIDFPPELCELKIIGFSKDIGSSISLLPSIMHRLENLLVAIE--------

---LKSLLSASFSEGAEVTA

---YRILEALTTER

CQER--LSLERLEILGDAFLKFAVGRHLFLLH----DTLDEGELTRKRSNAVNNSNLLKL

ASRRNLQVYIRDQPFDPRQFF---------------------------------------

------------------------------------ALG-----------HPCPVICTKE

SEGSIHS--SNRSNAKGQENTIEVRC---------------SRGHHWL---------YKK

TIADVVEALVGAFI-------------------------VDSG-----------------

---------------------------------------FRAATAFLKWLGIRVNIEASD

VTKVCLASRTFMPLAPSID----------------------VSSLEDSLDHQFVNRGLVL

QAFVHPSYNK-HGG-GCYQRLEFLGDAVLDYLITSYLFSVYPKLKPGLLTDLRSALVNNR

AFAIVAVDRSFNEFLICDSGNLSEAIETYVNFV---------------------------

---------------KRPAVE-----KDSLEGPK---CPKVLGDLVESCIGAIFLDTG--

--------------------------------FDLNCIWKLMLSFLDPIL----------

------------NSSNVLLNPF--------------------------------------

--RELHEFCESHK---------

--WKLQFP---TLKRD

MNFLVEA---

---KVTGKDICLDA----

----SANNSNKKEAIRIASEQIIVKL----------------------------------

------------------------K-------DQGYIRKSNYLEEVLRSGQKTDAK----

--------------------LIGYDETPIDITAH-DPIGL--QNLKIQDP----------

----------------------SCSD----FNPKIRSMSKLTNTCSPCFIAANIQPPSPS
VMVGGQPSATVAYPTSDMDKPT-------SAK----------------------------

-------------SRLHDICAAN-----------------CWKPPLFECC----------
YEEGPSHLKSFSYKVIVEIEAAPDMILECFGAPREKK-----------------------

-------------------------KAAAEHAAEGALWYLQHVGYLTVK-----------

>Arabidopsis_thaliana_DCL-1_ARATH
-------------------------------MVMEDEPREATIKPSYWLDACEDISCDLI
DDLVSEFDPSSVAVNESTDENGVINDFFGGIDHILDSIKNGGGLPNNGVSDTNSQINEVT
VTPQVIAKETVKENGLQKNGGKRDEFSKEEGDKDRKRARVCSYQSERSNLSGRGHVNNSR
EGDRFMNRKRTRNWDEAGNNKK-----------KRECNNYRRDGRDREVRGYWERDKVGS
NELVYRSGTWEADHERDVKKVSGGNRECDVKAEENKSKPEERKEKVVEEQARRYQLDVLE
QAKAKNTIAFLETGAGKTLIAILLIKSVH------------KDLMSQNRKMLSVFLVPKV
PLVYQQAEV---
-----------------------IRNQTC-----FQVGHYCGEM-------GQDFWDSRR
WQREFESK--------------QVLVMTAQILLNILRHSIIRMETIDLLILDECHHA--V
KKHPYSLVMSEFYHTTP---
--------KDKRPAIFGMTASPVNLKGVSSQ--------------VDCAIKIRNLETKLD
ST---VCTIKDRKE
L---------------------EKHVPMPS-----------------------------
EIVVEYDKAAT-MWSLHETIKQMIAAVEEAAQASSRKSKWQFMGARDAGAKDELRQVYGV
SE-------RTESDGA------ANLIHKLRAINYTLAELGQW--CAYKVGQSFLSA---L
QSDERVNFQVDVKFQE--
---------------------SYLSEVVSLLQCELLEGAAAEKVAAEVGKPEN----GNA
HDEMEEGELPD-----DPVVSG--------------------------------------
-------------------------GEHVDEVIGAAVADGKVTPKVQSLIKLLLKYQHT
ADFR---------------------AIVFVERVVAALVLPKVFAEL-PSLS-F------

----------------------IRCASMIGHNNSQEMKSS--------QMQDTISK-FRD
GHVTLLVATSVAEEGLDIRQCNVVMRFDLAKTVLAYIQSRGRARKPGSDYILMVERGNVS
HAAFLRNARNSEETLRKEAI--

---ERTDL
SHLKDTSRLISIDAVPG---

---TVYKVEATGAMVSLN
SAVGLVHFYCSQLPG-DRYAILRPEFSMEKHEKPGGHTEYS-------------------
------CRLQLPCNAPFEILEGPVCSSMRLAQQAVCLAACKKLHEMGAFTDM--------
------------------------------------LLPDKG------------------
--SG
QDAEKADQDDEGEPVPGTARHR----EFYPEGVADVLKGEWVSSGKEVCESS--------
------KLFHLYMYNVR---
-----------CVDFGSSKDPFLSE-----------------------------------

-VSEFAILFGNELDAEVL-SMSMDL-YVARAMITKASLAFKGSLDITENQLSSLK-----
---KFHVRLM
SIV---

-----LD-------VDVEPSTTP--------WDPAKAYLFVPVTDNTSMEPIKG------

-----INWELVE---------KITKTTAW--DNPLQRARPDVYLGTNERTLGGDRREYGF
GKLRHNIVFGQKSHPTYGIRGAVASFDVVRASGLLPVR--DAFEKEVEEDLSKGKLMMAD
GCMVAEDLIGKIVTAAHSGKRFYVDSI---------------------------------
------------------------------------CYDMSAETSFPRKEGYLGPLEYNT
YADYYKQ------------KYGVDLNCKQQPLIKGRGVSYCKNLLSPRFEQ----SGESE
---TVLDKTYYVFLPPELC--VVHPLSGSLIRGAQRLPSIMRRVESMLLAVQ--------
---LKNLI------SYPIPT

---SKILEALTAAS
CQET--FCYERAELLGDAYLKWVVSRFLFLKY----PQKHEGQLTRMRQQMVSNMVLYQF
ALVKGLQSYIQADRFAPSRWS---------------------------------------
------------------------------------APG----------VPPVFDEDTKD
GGSSFFD-------EEQKPVSEENSDVFEDGEMEDGELEGDLSSYRVL---------SSK
TLADVVEALIGVYY------------------------VEGG------------------
---------------------------------------KIAANHLMKWIGIHVEDDPDE
VDGTLKNVNVPESVLKSID----------------------FVGLERALKYEFKEKGLLV
EAITHASRPS-SGV-SCYQRLEFVGDAVLDHLITRHLFFTYTSLPPGRLTDLRAAAVNNE
NFARVAVKHKLHLYLRHGSSALEKQIREFVKEV---------------------------

---------------QTESSKPGFNSFGL-GDCK---APKVLGDIVESIAGAIFLDSG--
--------------------------------KDTTAAWKVFQPLLQPMV----------

------------TPETLPMHPV--------------------------------------
--RELQERCQQQA---------

--EGLEYK---ASRSG
NTATVEV---
---FIDGVQVGVAQ----
----NP---QKKMAQKLAARNALAAL----------------------------------
------------------------K-------EKEIAESKEKHINNGNAGEDQGEN----

--------------------ENGNKKNGHQP-----------------------------

-----------------------------FTR----------------------------

-------------QTLNDICLRK-----------------NWPMPSYRCV----------
KEGGPAHAKRFTFGVRVNTSDRGWTD-ECIGEPMPSV-----------------------

-------------------------KKAKDSAAVLLLELLNKTFS---------------

>Populus_trichocarpa_DCL-1_POPTR
MESESNGRVSGIGGGGGPSYWLDACEDISCDIIDDFVDFDTSIVPELSVDNNSNVNNDFF
GGIDHILDSIKNGSGLPPLHNASTTANVSNGNRDCIVGDGWFINVENGVCHGSSVSQSNG
GDKDNIDRKGQVENGGNGLNLSNGKREERFSNNFVKENGKKDEQSTEQGIDGDERAAEHR
DRERCSSRKRSRDWDESDRRDRDISRRRDRYSGSNRRDGRDRDWRERELRGYWERDRSGS
KDMVFRLGTWEADHNKEGREANDKIQECKGELEKKSEES---KEKVPEEQARQYQLDVLD
QAKKKNTIAFLETGAGKTLIAVLLIRSIC------------NDLQRQNKKILAVFLVPKV
PLVYQQAEV---
-----------------------IRER-G-----YQVGHYCGEM-------GQDFWDTRR
WQREFETK--------------QVLVMTAQILLNILRHSIIKMEAINLLILDECHHA--V
KKHPYSLVMSEFYHTTP---

--------KEKRPSVFGMTASPVNLKGVSSQ-------------VDCAIKIRNLESKLD
SI---VCTIKDRKE
L----------------------EKHVPMPA-----------------------------
EVVVEYDKAAS-LWSLHEQIKQIEAAVEEAAQSSSRRSKWQFMGARDAGAKEELRQVYGV
SE-------RTESDGA------ANLIQKLRAINYALGDLGQW--CAYKVAQSFLTA---L
QNDERANYQLDVKFQE--
---------------------SYLERVVLLLQCQLTEGAVTDKDTKVSDNGNDNIQDGPG
FDEIEEGELPD-----SHVVSG--------------------------------------
--------------------------GEHVDVIIGAAVADGKVTPKVQSLIKVLLRYQHT
EDFR----------------------AIIFVERVVAALVLP-------------------

-------------------------------------KSRGRARKPGSDYILMVERGNLS
HGAFLRNARNSEETLRKEAI--

---ERTDL
SHLKDTSRLIAVDSIPG---

---TVYQVESTGAVVSLN
SAVGLVHFYCSQLPS-DRYSILRPGFIMEKHEKPGGPTEYS-------------------
------CKLQLPCNAPFEELEGPA----------VCLAACKKLHEMGAFTDM--------
------------------------------------LLPDKG------------------
--SE
EEKDKVDQNDEGEPLPGTARHR----EFYPEGVAKTLQGEWILCGRDGCNNS--------
------KVLHLYLYGVR---
-----------CLNIGTSNDPFLTQ-----------------------------------

-VSNFAVLFGNELDAEVL-SMSMDL-FIARTMITKASLVFRGRIPITESQLASLK-----
---NFHVRLM
SIV---

-----LD-------VDVEPSTTP--------WDPAKAYLFVPMVSDKSVDPIKE------

-----IDWDLVE---------NIIGTDAW--SNRLQRARPDVYLGTNERTLGGDRREYGF
GKLRHGIAFGQKPHPTYGIRGAVAQFDVVKASGLIPKRGWDATETQ-KLELTKGKLMMAD
TCVNADALMGRIVTAAHSGKRFYVDSI---------------------------------
------------------------------------CYDMTAEISFPRKEGYLGPLEYSS
YADYYKQ------------KYGVELKFKQQPLLRGRGVSYCKNLLSPRFEHSDSNEGDAE

---ENLDKTYYVFLPPELC--LVHPLPGSLVRGAQRLPSIMRRVESMLLAVE--------
---LKDII------NYPVPA

---SKILEALTAAS
CQET--FCYERAELLGDAYLKWVVSRFLFLKY----PQKHEGQLTRMRQQMVSNMVLYQY
ALNKGLQSYIQADRFAPSRWA---------------------------------------
------------------------------------APG----------VLPVFDEETKD
GDSYIFD--QEKSLAEDRTGMNHLDDGY-ENEIEDGELESDASSYRVL---------SSK
TLADVVEALIGVYY------------------------VEGG------------------
---------------------------------------KNAVNHLMKWIGIQVEFDHEE
IDGASRPFNVPESVLRSVD----------------------FDTLEGALDIKFNDRGLLI
EAITHASRPS-SGV-SCYQRLEFVGDAVLDHLITRHLFFTYTNLPPGRLTDLRAAAVNNE
NFARVAVKHKLHVHLRHGSSALEKQIRDFVREV---------------------------

---------------QDELLKPVFNSFGL-GDCK---APKVLGDIVESIAGAIFLDSG--
--------------------------------RDTAVVWKVFQPLLHPMV----------

------------TPETLPMHPV--------------------------------------
--RELQERCQQQA---------

--EGLEYK---ATRSG
NLATVEV---
---FIDGVQVGVAQ----
-------NPQKKMAQKLAARNALVVL----------------------------------
------------------------K-------EKETAEAKEKSD---ENGKKKRNG----

--------------------NQTFTR----------------------------------

-------------QTLNDICLRR-----------------NWPMPSY-------------
---------------RMDRRMCGRADAEC-------------------------------

>HbDCL1

--MYPIGGRVEGYRRA
RRDKYHNRRRDRY--------------------NGRRDGRDRDWRDREPRGYWERDRSGS
NEMIFHSGSWEADHNKEGKESNDKDEECNGKPEKKSEET---KEKFPEEQARQYQLDVLE
QAKKRNTIAFLETGAGKTLIAVLLIKSLY------------NDLQRQNKKMLAVFLVPKV
PLVYQQAEV---
-----------------------IREHTS-----YQVGHYCGEM-------GQDFWDARR
WQPEFESK--------------QVLVMTAQILLNILRHSIIKMEAINLLILDECHHA--V
KKHPYSLVMSEFYHTTP---
--------KEKRPSVFGMTASPVNLKGVSSQ--------------VDCAIKIRNLESKLD
SI---VCTIKDRKE
L----------------------EKHVPMPA-----------------------------
EIVVEYDKAAS-LWSLHEQIKQMEVAVEEAAQSSSRRSKWQFMGARDAGAKEELRQVYGV
SE-------RTESDGA------ANLIQKLRAINYALGELGQW--CAYKVAQSFLMA---L
QNDERANYQLDVKFQE--
---------------------SYLEKVVLLLQCQLTEGAVTDKDTKSNDNENGVAQDGTD
PDDIEEGELPD-----SHVVSG--------------------------------------
--------------------------GEHVDVIIGAAVADGKVTPKVQSLIKILLKYQYT
EDFR----------------------AIIFVERVVAALVLPKVFAEL-PSLS-F------

----------------------VRCASLIGHNNSQEMRTS--------QMQDTIAK-FRD
GRVTLLVATSVAEEGLDIRQCNVVIRFDLAKTVLAYIQSRGRARKPGSDYILMVERGNLS
HGAFLRNARNSEETLRKEAI--

---ERTDL
SHLKDTSRLISVDTIPG---

--TVYQVESTGAIVSLN
SAVGLIHFYCSQLPS-DRYSILRPEFIMERHEKPGGPTEYS-------------------
------CKLQLPCNAPFEKLEGPVCSSMRLAQQAVCLAACKKLHEMGAFTDM--------
------------------------------------LLPDKG------------------
--SG
EEREKTDQNDEGEPLPGTARHR----EFYPEGVANILQGEWILCGRDGCNNS--------

------KLLKLYMYAVR---
-----------CVNSGTSKDPFLTQ-----------------------------------

-VSDFAVLFGNELDAEVL-SMSMDL-FIARTIITKASLVFRGPINVTETQLASLK-----
---SFHVRLM
SIV---

-----LD-------VDVEPSTTP--------WDPAKAYLFVPMVGDKFVDPIKE------

-----IDWDLVE---------KIIRTDVW--SNPLQRARPDVYLGTNERTLGGDRREYGF
GKLRHGMAFGQKSHPTYGIRGAVAQFDVVKASGLVPNR--DGTETE-KVGLPKGKLMMAD
TCVDAEDLVGRIVTAAHSGKRFYVDSI---------------------------------
------------------------------------RYDMTAENSFPRKEGYLGPLEYSS
YADYYKQ------------KYGVELIFKQQPLMRGRGVSYCKNLLSPRFEHSESNEGESE
---EILDKTYYVFLPPELC--LVHPLPGSLVRGAQRLPSIMRRVESMLLAVQ--------
---LKDMI------DYSVPA

---SKILEALTAAS
CQET--FCYERAELLGDAYLKWVVSRFLFLKY----PQKHEGQLTRMRQQMVSNMVLYQY
ALNKGLQSYIQADRFAPSRWA---------------------------------------
------------------------------------APG----------VLPVFDEDTKD
GESSLFD--QERLLSEDKPGVDHADDGYQDDEIEDGELESDSSSYRVL---------SSK
TLADVVEALIGVYY-------------------------VEGG-----------------
---------------------------------------KNAANHLMKWIGIQVEFDHEE
VDSAIKPANVPESILRSVN----------------------FDALEGSLNIKFNDRGLLV
EAITHASRPS-SGV-SCYQRLEFVGDAVLDHLITRHLFFTYTNLPPGRLTDLRAAAVNNE
NFARVAVKHKLHVHLRHGSSALEKQIRDFVKEV---------------------------

---------------QDELSKPGFNSFGL-GDCK---APKVLGDIVESIAGSIFLDSG--
--------------------------------RDTAVVWKVFQPLLHPMV----------

------------TPETLPMHPV--------------------------------------
--RELQERCQQQA---------

--EGLEYK---ASRSG
NLATVEV---
---FIDGIQVGLAQ----
----NP---QKKMAQKLAARNALAVL----------------------------------

------------------------K-------EKETAEAKEKGD---ENGTKKKNG----

--------------------NQTFTR----------------------------------

-------------QTLNDICLRR-----------------NWPMPLYRCV----------
NEGGPAHAKRFTFAVRVNTNDRGWTD-ECVGEPMPSV-----------------------

-------------------------KKAKDSAAVLLLELLNKWYS---------------

>Ricinus_communis_DCL-1_RICCO

-------------------------MTEGESRVSGTVGGGGDTSYWLDACEDISCDLIDN
LVDFDTSIVQDSVVVDDTSNQDHLVNDFFGGIDHILDSIKNGSGLPPLSDTINNYSNSNS
LTTAVAVVGNNGGIQDCNVGEGWFTNEAADVCTNGVQSSSLHLNGSDKNIESKGQLEIFD
NGLS----LLVGEGEERLNLLDNSVRENGSRGEGQRERDIDSDVRSCKRARITSYKNERQ
YSSRGQYDA-------------------RDRDRSSNRKRLRDWDDIDRRDRDYLRRRDRY
GGGGGRRDGKDRDLRDREPRGYWERDRSGSSEMVFRVGTWEADRNKGKEVNGKDQESNGK
AEKKVEETKEKIPEEQARQYQLDVLVMTAQILLNILRHSIIKMEAINLLILDECHHA--V
KKHPYSLVMSEFYHTTL---
--------KEKRPSVFGMTASPVNLKGVSSQ--------------VDCAIKIRNLESKLD
SI---VCTIKDRKE
L----------------------EKHVPMPS-----------------------------
EIVVEYDKAAT-LWSLHEKIKQMEVAVEEAAQSSSRRSKWQFMGARDAGAKEELRQVYGV
SE-------RTESDGA------ANLIQKLRAINYALGELGQW--CAYKVAQSFLMA---L
QSDERANYQLDVKFQE--
---------------------SYLEKVVSLLQCQLTEGAVTDKDTKSPENENGVAQAGSD
PDDIEEGELPD-----SHVVSG--------------------------------------
--------------------------GEHVDVIIGAAVADGKVTPKVQSLVKILLKYQHT
EDFR----------------------AIIFVERVVAALVLPKVFAEL-PSLS-F------

----------------------VRCASLIGHNNSQEMRTS--------QMQDTIAK-FRD
GRVTLLVATSVAEEGLDIRQCNVVIRFDLAKTVLAYIQSRGRARKPGSDYILMVERGNLS
HGAFLRNARNSEETLRREAI--

---ERTDL
SHLKDTSRLISVDSVPG---

---TVYQVESTGAIVSLN
SAVGLIHFYCSQLPS-DRYSILRPEFIMERHEKPGGPTEYS-------------------
------CKLQLPSNVPFEKLEGPLCSSMRLAQQAVCLAACKKLHEMGAFTDM--------
------------------------------------LLPDKG------------------
--SG
EEREQVDQNDEGEPLPGTARHR----EFYPEGVANILQGEWILCGRDGWNNS--------
-----NKLLHLYMYAVK---
-----------CVNSGASKDPFLTQ-----------------------------------

-VSEFAVLFGNELDAEVL-SMSMDL-FIARTIITKASLVFRGPIDITENQLASLK-----
---SFHVRLM
SIV---

-----LD-------VDVEPSTTP--------WDPAKAYLFVPMVGDKFVNPIKE------

-----IDWDLVE---------NVIRTDVW--SNPLQRARPDVYLGTNERTLGGDRREYGF
GKLRHGMAFGQKSHPTYGIRGAVAQFDVVKASGLVPNR--AGVEIQ-KVELPKGKLMMAD
SYVGAEELVGRIVTAAHSGKRFYVDSI---------------------------------
------------------------------------RYDMTAENSFPRKEGYLGPLEYSS
YADYYKQ------------KYGVELMFKQQPLIRGRGVSYCKNLLSPRFEHSDSNEGESE
---EILDKTYYVFLPPELC--LVHPLPGSLVRGAQRLPSIMRRVESMLLAIQ--------
---LKDII------HYSVPA

---LKILEALTAAS
CQET--FCYERAELLGDAYLKWVVSRFLFLKY----PQKHEGQLTRMRQQMVSNMVLYQY
ALIKGLQSYIQADRFAPSRWA---------------------------------------
------------------------------------APG----------VLPVFDEDTKD
GDSSLFD--QEKSLIENKPKVDHADDGYEDDEIEDGELESDSSSYRVL---------SSK
TLADVVEALIGIYY------------------------VEGG------------------
---------------------------------------KTAANHLMRWIGIKVEFDHEE
IDSAIRPSNVPESILRSID----------------------FDALEGALNIKFQDRGLLV
EAITHASRPS-SGV-SCYQRLEFVGDAVLDHLITRHLFFTYTNLPPGRLTDLRAAAVNNE
NFARVAVNHKLHVHLRHGSSALEKQIRDFVREV---------------------------

```
------------------------------------------------------------
------------------------------------------------------------
---------------QDELSKPGFNSFGL-GDCK---APKVLGDIVESIAGAIFLDSG--
--------------------------------RDTAVVWKVFQPLLHPMV----------
------------------------------------------------------------
------------------------------------------------------------
------------------------------------------------------------
------------TPETLPMHPV--------------------------------------
----------------------------------------RELQERCQQQA---------
------------------------------------------------------------
------------------------------------------------------------
----------------------------------------------EGLEYK---ATRSG
NLATVEV-----------------------------------------------------
---------------------------------------------FIDGVQVGVAQ----
----NP---QKKMAQKLAARNALVIL----------------------------------
------------------------K-------DKETAEAKEKGN---DNGKKKKNG----
------------------------------------------------------------
--------------------NQTFTR----------------------------------
------------------------------------------------------------
------------------------------------------------------------
------------------------------------------------------------
------------------------------------------------------------
------------------------------------------------------------
------------------------------------------------------------
-------------QTLNDICLRR-----------------NWPMPFYRCV----------
NEGGPAHAKRFTFAVRVNTTDRGWTD-ECVGEPMPSV-----------------------
------------------------------------------------------------
------------------------------------------------------------
------------------------------------------------------------
------------------------------------------------------------
-------------------------KKAKDSAAVLLLELLNKRYS---------------
------------------------------------------------------------
------------------------------------------------------------
------------------
>Arabidopsis_thaliana_DCL-2_ARATH
------------------------------------------------------------
------------------------------------------------------------
------------------------------------------------------------
------------------------------------------MTMDADAMETETTDQVSA
SPLHF---------------------------------------------ARSYQVEALE
KAIKQNTIVFLETGSGKTLIAIMLLRS-Y------------AYLFRKPSPCFCVFLVPQV
VLVTQQAEA---------------------------------------------------
-----------------------LKMHTD-----LKVGMYWGDM-------GVDFWDSST
WKQEVDKY--------------EVLVMTPAILLDALRHSFLSLSMIKVLIVDECHHA--G
```

GKHPYACIMREFYHKEL---
-----NSGTSNVPRIFGMTASLVKTKGENLD--------------S-YWKKIHELETLMN
SK---VYTCENESV
L----------------------AGFVPFST-----------------------------
PSFKYYQHIKI-PSPKRASLVEKLERL---------TIKHRLS-----------LGTLDL
NS-------STVDSVE------KRLLRISSTLTYCLDDLGIL--LAQKAAQ-SLSA---S
QNDSFLWGELNM-FSV--
---------------------ALVKKF------------------------------CSD
ASQEFLAEIPQ-----GLNWSV--------------------------------------
--------------------------A-NINGNAEA----GLLTLKTVCLIETLLGYSSL
ENIR----------------------CIIFVDRVITAIVLESLLAEILPNCN-N------

----------------------WKTKYVAGNNSGLQNQTR--------KKQNEIVEDFRR
GLVNIIVATSILEEGLDVQSCNLVIRFDPASNICSFIQSRGRARMQNSDYLMMVESGDLL
TQSRLMKYLSGGKRMREESL--

---DHSLV
P-----CPPLPDDS-DE---

---PLFRVESTGATVTLS
SSVSLIYHYCSRLPS-DEYFKPAPRFDVNKDQG-------S-------------------
------CTLYLPKSCPVKEVK--AEANNKVLKQAVCLKACIQLHKVGALSDH--------
------------------------------------LVP---------------------
--DM
VVAETVSQKLEKIQYNTEQPC------YFPPELVS-----------QFSAQP--------
-----ETTYHFYLIRMK---
-----------------PNSPRNFH-----------------------------------

-LNDVLLGTRVVLEDDIG-NTSFRL-EDHRGTIA-VTLSYVGAFHLTQEEVLFCR-----
---RFQITLF
RVL---

-----LDH------SVENLMEALNGLH--LRDGVALDYLLVP-STHSHETSL--------

-----IDWEVIR---------SVNLTSHEVLEK---------------------------
-------------HENCSTNG---------ASRILHTK--DGLFCT--------------
-CV----VQNALVYTPHNGYVYCTKGV---------------------------------
------------------------------------LNNLNGNSLLTKRNSG-----DQT

YIEYYEE------------RHGIQLNFVDEPLLNGRHIFTLHSYLHMAKKK-----KEKE
------HDREFVELPPELCHVILSPISVDMIYSYTFIPSVMQRIESLLIAYN--------
---LKKSI-----PKVNIPT

---IKVLEAITTKK
CEDQ--FHLESLETLGDSFLKYAVCQQLFQHC----HTHHEGLLSTKKDGMISNVMLCQF
GCQQKLQGFIRDECFEPKGWM---------------------------------------
------------------------------------VPG------------------QSS
AAYSLVN-----------DTLPESRNIY-------------VASRRNL---------KRK
SVADVVESLIGAYL-------------------------SEGG-----------------
---------------------------------------ELAALMFMNWVGIKVDFTTTK
IQR-----DSPIQAEKLVN----------------------VGYMESLLNYSFEDKSLLV
EALTHGSYMM-PEIPRCYQRLEFLGDSVLDYLITKHLYDKYPCLSPGLLTDMRSASVNNE
CYALVAVKANLHKHILYASHHLHKHISRTVSEF---------------------------

---------------EQSSLQ---STFGWESDIS---FPKVLGDVIESLAGAIFVDSG--
--------------------------------YNKEVVFASIKPLLGCMI----------

------------TPETVKLHPV--------------------------------------
--RELTELCQK-----------

--WQFELS---KAKDF
DSFTVEV---
---KAKEMSFAH----
----TAKASDKKMAKKLAYKEVLNLL----------------------------------
------------------------K-----------------------------------

-------------NSLDY--

>Populus_trichocarpa_A_DCL-2_POPTR

--MEPVSVDIDTTQQLPA
DPLPF---ARSYQLEALE
QALKQNTIVFLETGSGKTLIATMLLRS-Y------------AHLLRKPSRFIAVFLVPEV
FLVRQQAGV---
-----------------------VRMHTD-----LNVGMYWGDM-------GIDFSHAAT
WKQEIDKH--------------EVLVMTHQILLNGLRQGYFKLDFIKVLIFDECHHA--R
GNHPYACIMTEFFHREL---
-----RSGHHDLPRIFGMTASLIKSKGANSE--------------SYYRQQICELENIMN
SK---VYTCASETV
L----------------------AEFIPSPA-----------------------------
AEFLFYEPMKI-PDGIYACLEEELGNL---------KAKHELL-----------LKQLDL
SE-------SAAESVH------SKISKVHSALMFCSGELGVW--LAFQAAR-FLSHSD-T
DSDFIAWGKVDV-SGE--
---------------------TIVKKF------------------------------CWD
ASLVISNCFS-------AECCI--------------------------------------
--------------------------GDNTEADVGA----GLITAKVLCLIKTLLQYRDL
KDIR----------------------CIVFVERVITAVVLESLLRELLPKHS-S------

----------------------WKTKYIAGNNSGLQSQTR--------QMQNEIVEEFRK
GMVNIIVATSILEEGLDVQSCNLVIRFDPPSSVSSFIQSRGRARMQNSDYLLMVKTEDST
THSRLENYLSSSEIMRRESL--

---RRSST
S-----CSAPQSELYED---

---EFYSVEGTGAVVTLS
SSVSLIYFYCSRLPS-DGYFKPAPICIIDKEKE-------T-------------------
------CTLHLPKSSPIQNIC--VQGNNKNLKQKACLEACKQLHLIGALTDN--------
------------------------------------LVP---------------------
--DV

VEEEAVAQEIRNERYDDEQPI------YLPPELAS-----------QGPRNL--------
-----KTKYYCYLIELN---
-----------------QKFDYGVP-----------------------------------

-VHDIVLVMRTELESDVLSSMGFEL-EAERGLLA-VSLRYIGDIYLDQVPVLLCR-----
---RFQITLF
EVL---

-----IHR------EVNKLEEVLKGLE--LGTGVVMDYFLLP-AIRSRSQPS--------

-----IDWEPIS---------SVLFS--YKNED---------------------------
-------------HFNCSSKG---------NAHVVHTK--GGPVCT--------------
-CV----LQNSLVCTPHNGNVYFITGA---------------------------------
------------------------------------SEDLNGRSLLKLRNGS-----AIT
YKEHFAKR-----------RNSIQLLFDQEPLLEGRHIFPVHNFLNRCRTK-----KEKE
------SKNAHVDLPPELCDIILSPVSISTLYSYTFIPSIMHRLESLLIAVN--------
---LKKMHSDHCMQNVDIPA

---MKVLEAITTKK
CQEK--FHLESLETLGDSFLKYAASQQLFKLY----QNHHEGLLSMKKEKIISNAALCRR
GCDHKLPGFIRNESFDPKLWM---------------------------------------
------------------------------------IPG-------------------DK
CGSDLLS----------EEPLSECRKIY-------------VRGRRKV---------KSK
TVADVVEALIGAYL-------------------------STGG-----------------
---------------------------------------EVLALFFMDWIGIKVDFMIVP
YER-----HFQLQAEKFVN----------------------VRYLESLLNYSFRDPSLLV
EALTHGSYML-PEIPSCYQRLEFLGDAVLDYLITMHLYKEYPGMSPGLLTDLRSASVNND
CYAQSAVKGDLHKHILHTSQDLHKHIVETAEIF---------------------------

---------------QKSSLG---STFGWESETS---FPKVLGDVIESLAGAILVDSG--
--------------------------------YNKEIVFQSIRPLLEPLI----------

------------TPATVRLHPA--------------------------------------
--RELSELCQKQH---------

--FDYKKSV--VSYNG
RNASITI---
---VVGANGVTFKH----

----TATAADKKTAKKLASKEVLKSL----------------------------------
------------------------K-------ESNFASSSTP------------------

-------------SKLD---

>Populus_trichocarpa_B_DCL-2_POPTR

--MEPVSMDIDTTQQLPA
DPLPF---ARSYQLEALE
KALKHNTIVFLETGSGKTLIAIMLLRS-Y------------AYLLRKPSRFIAVFLVPQV
VLVRQQAGA---
-----------------------VEMHTD-----LKVGMYWGEM-------GVDFWNAAT
WKKEIEKH--------------EVLVMTPQILLNGLRHSFFKLDLIKVMIVDECHHT--R
GKHPYASIMTEFFHCEL---
-----KSGHHDLPRIFGMTASPIKSKGANSE--------------LYYWQQIRELEDIMN
SKLLSPFAFGSLLQDEDDPHGTFQMHSTNIHVISHSCSKVFENCIPGTRHMIYTCVSESA
L----------------------AEFIPFST-----------------------------
PKFLFYEHMKI-PDGIFASLLKELGNL---------RTKHEHM-----------LEQLDL
NE-------SAAVSIC------NKISKVHSALMFCLEELGVW--LAFQAAQ-FLSHCD-T
DGDFISGGKLDV-SGE--
---------------------TIVKNF------------------------------CQD
ASLAISNCFSD-----GQECSI--------------------------------------
--------------------------GDNIKAHLGA----GLLTSKILCLVESLLQYRDL
KEIR----------------------CIVFVERVITAIVLESLLSKLLPKHG-S------

----------------------WKTKYIAGNNSGLQSQTR--------KIQNEIVEEFRK
GMVNIIVATSILEEGLDVQSCNLVIRFDPSATVSSFIQSRGRARMQNSDYLLMVKRGDFS

THARLENYLASGDIMRRESL--

---RHASI
P-----CSPL-LDELDD---

---EFYRVEGTGAVVSLS
SSVSLIYFYCSRLPS-DGYFKPAPRCIIDKETM-------T-------------------
------CTLHLPKSSPVQTIC--VQGNIKTLKQKACLEACKKLHVSGALTDN--------
------------------------------------LVP---------------------
--DI
VMEEAVAEDVGNERYDDEQPI------YLPPELVS-----------RGPRNL--------
-----KTKYYCYLIELN---
-----------------QNFAYDIP-----------------------------------

-VHDVVLVVRTELESDVIRSMGFDL-EAERGLLT-VNLRYIGDIDLERVLVLLCR-----
---RFQITLF
KVL---

-----LDH------SVNKLKEVLEGLD--LGSGAEIDYFLLP-AFRSCSQPS--------

-----INWAPIS---------SVLFS--YKNEE---------------------------
-------------HFNCSRNG---------NAHVVQTK--CGPVCA--------------
-CV----LQNSLVCTPHNGNIYCITGV---------------------------------
------------------------------------FEDLNGNSLLKMGDGG-----AIT
YKEYFAKRPMSDLKLTLDFRHGIQLLFNREPLLKGKHIFPVHNLLNRCRKQ-----KEKA
------SKNTHVELPPELCEIILSPISISTLYSYTFIPSIMHRLESLLIAVN--------
---LKKMHSDHYLQHVNIPS

---MKVLEAITTNK
CQEN--FNLESLETLGDSFLKYAASQQLFKIY----QNHHEGLLSFKKDKIISNAALCRR
GCNHKLQGFIRNESFDPKLWI---------------------------------------
------------------------------------IPG-------------------GK
LGSDFLS----------EEPLSKGRKIY-------------IRGRRKV---------KSK
TIADVVEALIGAYL-------------------------STGG-----------------
---------------------------------------EVTALLFMDWIGIKVDFMNTP
YER-----HIQLQAEKFVN----------------------VRYLESLLNYSFNDPSLLV
EALTHGSYML-PEIPRCYQRLEFLGDAVLDYLITLHMYKEYPGMSPGLLTDLRSASVNND
CYALSAVKVGLDRHILHASHDLHKHIVATVKKI---------------------------

---------------QEFSLE---STFGWESETA---FPKVLGDVIESLAGAILVDSG--
--------------------------------YNKEVVFESIRPLLEPLI----------

------------TPETLRLQPV--------------------------------------
--RELNELCQRQH---------

--FDYKKPI--VSRNG
RNASVTI---
---EVEANGLIFKH----
----TATVADKTTAKKLASKEVLKAL----------------------------------
------------------------K-------ESNFATSTASKVE---------------

>Ricinus_communis_DCL-2_RICCO

--MEPVYMDVDSDQRLAS
DPLPF---ARSYQLEALE
KAIQQNTIVFLETGSGKTLIAIMLLRS-Y------------AHLLRKPSPFIAVFLVPQV
VLVKQQAEA---
-----------------------VETHTD-----LTVGKYWGEM-------GVDFWDAGS

WKQQIEQY--------------EVLVMTPQILLDGLRHSFFKLDCIKVLIFDECHHA--R
GKHPYACILTEFYHRQL--
-----TYRDSALPRIFGMTASPIKSKGAKSE--------------LAYWTEIRELENIMY
SK---VYTCASESV
L---------------------AEFIPFST----------------------------
PKFKFYKPMDI-PYAIYASLAENLKIL---------KSKYECN-----------LKLLDL
TD-------AVVESTS------KRISKMHSTLMYCLDELGVW--LALKAAQILSCHES--
--EFFSCGKLDI-SGE---
---------------------NIVKEF-----------------------------GLN
ASQALDNCINS-----GPKWSI-------------------------------------
-------------------------GENAEADIEA----GLLTTKIFCLIDSLLDYRDI
RDLR----------------------CIIFVNRVIAAIVLQTLLGELLPRYNSG------

---------------------WKTQYIAGNNFRLQSQSR--------KTQNEIVEEFRE
GKVNIIVATSILEEGLDVQSCNLVVRFDPSTTVSSFIQSRGRARMQNSDYLLMVKSGDVS
THSRLENYLASGDLMRKESI---------------------------------------

--RHGSV
P-----CSPIRSEFHEG--

---EYYFVESTNALVTLT
SSVSLIYFYCSRLPS-DGYFKPTPRCLIDKEME-------R-------------------
------CTLLLPKSCSIHTIS--VEGNVKIIKQKACLEACKQLHKIGALNDN--------
-----------------------------------LVP---------------------
---DI
VVEETVAQQSGNGPYDDEHPM------YFPPELVG-----------QASQKS--------
-----EAKYYCYLIELN--
-----------------QNFVYEIP----------------------------------

-VHNFVLAMRSELESDIL-GLDLDL-EADRGLLM-VKLKYIGEIHLTPETVIMCR-----
--KFLITVL
KVL--

-----VDH------SIDKLEDILKGLK--LRNGPEIDYLLLP-LVGSCQKPS--------

-----IDWDAVT---------SVLFS--Y--ENVLED----------------------
-------------HKNCPLKE---------AACVIQTK--DGVVCK-------------
-CT----LQNSVVYTPHNGKVYFIDGT--------------------------------

------------------------------------LDHLNGHSLLELRNGY-----HKS
YMEYYKD------------QHGIKLHFDQQLLLRGRHIFPLQNYLNRCRQQ-----KEKD
------SQNAYVELPPELCHIFMSPISISSFYSFTFVPSIMHRLESLLIASN--------
---LKKLHLDHCMQSVAIPT

---IKVLEAITTKK
CQEK--FHLESLETLGDSFLKYAVGQQLFKTY----QNHHEGLLSIKKDKLISNATLCRL
GCDSKIPGFIRNESFDPKNWL---------------------------------------
------------------------------------IPD-------------------EI
SGCYSLS----------EEILSNGRKMY-------------IKRRRKL---------KEK
MIADVVEALIGAYL-------------------------STGG-----------------
---------------------------------------EIAGLLFLDWIGIKADFLNMP
YER-----GFEMNPEKYVN----------------------ICHLESLLKYSFRDPCLLV
EALTHGSYML-PEIPRCYQRLEFLGDSVLDYLITVHLYEKYPGMSPGLLTDMRSASVNND
CYAQSAVREGLHKYILHASQKLHKDIVSTVANC---------------------------

---------------KEFSSE---STFGWESEIS---FPKVLGDVIESLAGAIFVDSG--
--------------------------------YNKEVVFNSIRPLLEPLI----------

------------TPETIRLHPT--------------------------------------
--RELTELCQKQH---------

--FDRRKPV--VSRNN
GMSCVTV---
---EVEANGVVFKH----
----TSAAAERKTAKRLASKEVLRAL----------------------------------
------------------------K-------DSLACK----------------------

>HbDCL2.1

---MDIDDAQKLGA
DPLPS---ARSYQLEALE
MALKQNTVLFLETGSGKTLVAIMLLRS-Y------------AYLIRKPSHFIAVFLVPHV
VLVQQQAEA---
-----------------------VEMHTD-----FNVGQLYGEK-------GVDCWDAAI
WKQHLEKH--------------EVLVMTPQILLDGLKHSFFKLDLIKVLIFDECHHA--R
GNHPYACILTEFYHRQL---
-----SCGNFNLPRIFGMTASPIKSKGANSE--------------QAYGQKIFDLENMMH
SK---VFLSF---F
L----------------------LGFAP--------------------------------
-------------------------------------QRFLCS-----------LNSIDF
SG-------LMIYICT------DASRFIVNKIFYSRE-----------AAQIMSSHGS--
--NFPSWDKLDV-FGD--
--------------------NIVKEF-------------------------------GFV
ASQEFDNCIRTAIEHLGPKWSI--------------------------------------
--------------------------GDNAKYDVDA----GLLTTKIFCLIDSLLEYRDL
NDIR----------------------CIVFVERVITAI----------------------

--VNIIVATSILEEGLDVQSCNLVVRFDPSATISSFIQSRGRARMHNSDYLLMVKSGDLS
TCSRLQNYLSSGDIMRKESI--

---RLASV
P-----CSPLKIELRDD---

---EFYCVESTGAVVTLT
SSVRLIYFYCSRLPS-DGYFKPAPRCVIDKERE-------V-------------------
------CTLHLPKSCDIQNIC--VQGNIKTLKQKACLEACKELHKIGALTDN--------
------------------------------------LVP---------------------

--DI
VMEEVVAQEVGNMPYDDEQPM------YFPPELVS-----------QGSKES--------
-----KGTYYCYLIELN---
-----------------QNFHSDIP-----------------------------------

-VHNIILVMGSELESDIL-SLDFEL-EVDRGLLT-VKLKYIGELDLTTEMVLICR-----
---KFLIALS
KVL---

-----VDH------NVNELEEILNGLR--LRKDPEIDYFLLP-SMASCQKPV--------

-----IDWSSIV---------SVLFS--Y--ANAWED-----------------------
-------------HVKCPLKD---------SAHIIWTK--NGQVCK--------------
-CM----LENSLVYTPHNGEVYCIKGI---------------------------------
------------------------------------FNHLDGRSLLRLKNGD-----CKT
YKEYYKD------------KHGINLSFDQQLLLRGRHIFPLQNYLDRCRQH-----KGKD
------SQNAYVQLPPELCCIKMSPISISTFYSFTFVPSIMHRLESLLIAVN--------
---LKKLHLDQ-MRNVIIPT

---IKVLEAITTKK
CQEK--FHLESLETLGDSFLKYAASQQLFKTY----QNYHEGLLSIKKEKLISNATLCKL
GCDRKLPGFIRNESFDPKNWM---------------------------------------
------------------------------------IPG-------------------DT
HGYSLHE-----------EFLSNARSIY-------------IIRRRKL---------KRK
VVADVVEALIGAYL-------------------------SAGG-----------------
---------------------------------------ETAALLFLNWIGIEVDFLNTP
YEP-----QFKVNPEKYIN----------------------IRRLESLLNYSFKNPSLLL
EALTHGSYML-PEIPRCYQRLEFLGDSVLDYLITVYLYEKYPGMSPGLLTDMRSASVNND
CYAFSAVREGLHKHILHLSQILHKDIAVAVENF---------------------------

---------------ENCSSE---STFGWESDVS---FPKVLGDVIESLAGAIFVDSG--
--------------------------------YDKEVVFQSIKPLLEPLI----------

------------TPETLKLHPA--------------------------------------
--RELNELCQVQH---------

--FEERKPV--SSRSN
GMSSVTV---

---EVEANGLIFKH----
----TSSAGDKRTAKKLASKELST------------------------------------
--------------------------------CRGALTQDQVRSVAGSAG----------

--FWPGRQSDT-----------
RAQGMTSLAFSGGLVRRPAVGWCWGGTSARGGERREK-----------------------

-------------------------MGEREEMLGGRERRKKKKKRSV-------------

>HbDCL2.2

--MTFN
IFSGCENLNTEKIKMDETLKGS-----------NLLLFPGSSAVMEPAYMDIDDAQKLGA
DPLPS---ARSYQLEALE
MALKQNTVLFLETGSGKTLVAIMLLRS-Y------------AYLIRKPSHFIAVFLVPHV
VLVQQQAEA---
-----------------------VEMHTD-----FNVGQLYGEK-------GVDCWDAAI
WKQHLEKH--------------EVLVMTPQILLDGLKHSFFKLDLIKVLIFDECHHA--R
GNHPYACILTEFYHRQL---
-----SCGNFNLPRIFGMTASPIKSKGANSE--------------QAYGQKIFDLENMMH
SK---VYTCVSDSV
L----------------------AEFIPFST-----------------------------
PKFKFYNHKDI-PYAIYVSLVEKLKIL---------KAKHECK-----------LKQLDL
TD-------SVVESTS------KNISKMHSTLMYCLDELGVW--LAFKAAQIMSSHGS--
--NFPSWDKLDV-FGD--
---------------------NIVKEF------------------------------GFV
ASQEFDNCIRT-----GPKWSI--------------------------------------
--------------------------GDNAKYDVDA----GLLTTKIFCLIDSLLEYRDL
NDIR----------------------CIVFVERVITAIVLQSLLSELLPRYN-G------

----------------------WKSKYIAGNGSGLQTQTQ--------KTQNEIVEEFRK

GKVNIIVATSILEEGLDVQSCNLVVRFDPSATISSFIQSRGRARMHNSDYLLMVKSGDLS

TCSRLQNYLSSGDIMRKESI--

---RLASV

P-----CSPLKIELRDD---

---EFYCVESTGAVVTLT

SSVRLIYFYCSRLPS-DGYFKPAPRCVIDKERE-------V-------------------

------CTLHLPKSCDIQNIC--VQGNIKTLKQKACLEACKELHKIGALTDN--------

------------------------------------LVP---------------------

--DI

VMEEVVAQEVGNMPYDDEQPM------YFPPELVS-----------QGSKES--------

-----KGTYYCYLIELN---

-----------------QNFHSDIP-----------------------------------

-VHNIILVMGSELESDIL-SLDFEL-EVDRGLLT-VKLKYIGELDLTTEMVLICR-----

---KFLIALS

KVL---

-----VDH------NVNELEEILNGLR--LRKDPEIDYFLLP-SMASCQKPV--------

-----IDWSSIV---------SVLFS--Y--ANAWED-----------------------

-------------HVKCPLKD---------SAHIIWTK--NGQVCK--------------

-CM----LENSLVYTPHNGEVYCIKGI---------------------------------

------------------------------------FNHLDGRSLLRLKNGD-----CKT

YKEYYKD------------KHGINLSFDQQLLLRGRHIFPLQNYLDRCRQH-----KGKD

------SQNAYVQLPPELCCIKMSPISISTFYSFTFVPSIMHRLESLLIAVN--------

---LKKLHLDQ-MRNVIIPT

---IKVLEAITTKK

CQEK--FHLESLETLGDSFLKYAASQQLFKTY----QNYHEGLLSIKKEKLISNATLCKL

GCDRKLPGFIRNESFDPKNWM---------------------------------------

------------------------------------IPG-------------------DT

HGYSLHE-----------EFLSNARSIY-------------IIRRRKL---------KRK

VVADVVEALIGAYL-------------------------SAGG-----------------

---------------------------------------ETAALLFLNWIGIEVDFLNTP

YEP-----QFKVNPEKYIN----------------------IRRLESLLNYSFKNPSLLL

EALTHGSYML-PEIPRCYQRLEFLGDSVLDYLITVYLYEKYPGMSPGLLTDMRSASVNND

CYAFSAVREGLHKHILHLSQILHKDIAVAVENF---------------------------

---------------ENCSSE---STFGWESDVS---FPKVLGDVIESLAGAIFVDSG--
--------------------------------YDKEVVFQSIKPLLEPLI----------

------------TPETLKLHPA--------------------------------------
--RELNELCQVQH---------

--FEERKPV--SSRSN
GMSSVTV---
---EVEANGLIFKH----
----TSSAGDKRTAKKLASKEVLKAL----------------------------------
------------------------K-------DAFNI-----------------------

